# GENCODE: massively expanding the lncRNA catalog through capture long-read RNA sequencing

**DOI:** 10.1101/2024.10.29.620654

**Published:** 2024-10-31

**Authors:** Gazaldeep Kaur, Tamara Perteghella, Sílvia Carbonell-Sala, Jose Gonzalez-Martinez, Toby Hunt, Tomasz Mądry, Irwin Jungreis, Carme Arnan, Julien Lagarde, Beatrice Borsari, Cristina Sisu, Yunzhe Jiang, Ruth Bennett, Andrew Berry, Daniel Cerdán-Vélez, Kelly Cochran, Covadonga Vara, Claire Davidson, Sarah Donaldson, Cagatay Dursun, Silvia González-López, Sasti Gopal Das, Matthew Hardy, Zoe Hollis, Mike Kay, José Carlos Montañés, Pengyu Ni, Ramil Nurtdinov, Emilio Palumbo, Carlos Pulido-Quetglas, Marie-Marthe Suner, Xuezhu Yu, Dingyao Zhang, Jane E. Loveland, M. Mar Albà, Mark Diekhans, Andrea Tanzer, Jonathan M. Mudge, Paul Flicek, Fergal J Martin, Mark Gerstein, Manolis Kellis, Anshul Kundaje, Benedict Paten, Michael L. Tress, Rory Johnson, Barbara Uszczynska-Ratajczak, Adam Frankish, Roderic Guigó

**Affiliations:** 1. Centre for Genomic Regulation (CRG), The Barcelona Institute of Science and Technology, Dr. Aiguader 88, Barcelona 08003, Catalonia, Spain.; 2. Departament de Ciències Experimentals i de la Salut, Universitat Pompeu Fabra (UPF).; 3. European Molecular Biology Laboratory, European Bioinformatics Institute, Wellcome Genome Campus, Hinxton, Cambridge CB10 1SD, UK.; 4. Department of Computational Biology of Noncoding RNA, Institute of Bioorganic Chemistry, Polish Academy of Sciences, Noskowskiego 12/14, 61-704 Poznan, Poland.; 5. Computer Science and Artificial Intelligence Lab, Massachusetts Institute of Technology, 32 Vassar St, Cambridge, MA 02139, USA.; 6. The Broad Institute of MIT and Harvard, 415 Main Street, Cambridge, MA 02142, USA.; 7. Flomics Biotech, SL, Carrer de Roc Boronat 31, 08005 Barcelona, Catalonia, Spain.; 8. Program in Computational Biology and Bioinformatics, Yale University, New Haven, Connecticut 06520, USA.; 9. Department of Molecular Biophysics and Biochemistry, Yale University, New Haven, Connecticut 06520, USA.; 10. Department of Life Sciences, Brunel University London, Uxbridge, London, UB8 3PH, UK.; 11. Bioinformatics Unit, Spanish National Cancer Research Centre (CNIO), Calle Melchor Fernandez Almagro, 3, 28029 Madrid, Spain.; 12. Department of Computer Science, Stanford University, Stanford, CA, USA.; 13. Hospital del Mar Research Institute, Dr. Aiguader 88, Barcelona 08003, Spain.; 14. Department of Medical Oncology, Bern University Hospital, Murtenstrasse 35, 3008 Bern, Switzerland.; 15. School of Biology and Environmental Science, University College Dublin, University College Dublin, Belfield, Dublin 4, D04 V1W8, Ireland.; 16. Catalan Institute for Research and Advanced Studies (ICREA), Barcelona, Spain.; 17. UC Santa Cruz Genomics Institute, 2300 Delaware Avenue, University of California, Santa Cruz, CA 95060, USA.; 18. University of Vienna, Research Network Data Science, Kolingasse 14-16, 1090 Vienna, Austria.; 19. University of Vienna, Faculty of Computer Science, Research Group Visualization and Data Analysis, Waehringerstrasse 29, 1090 Vienna, Austria.; 20. Department of Genetics, Stanford University, Stanford, CA, USA.

## Abstract

Accurate and complete gene annotations are indispensable for understanding how genome sequences encode biological functions. For twenty years, the GENCODE consortium has developed reference annotations for the human and mouse genomes, becoming a foundation for biomedical and genomics communities worldwide. Nevertheless, collections of important yet poorly-understood gene classes like long non-coding RNAs (lncRNAs) remain incomplete and scattered across multiple, uncoordinated catalogs, slowing down progress in the field. To address these issues, GENCODE has undertaken the most comprehensive lncRNAs annotation effort to date. This is founded on the manual annotation of full-length targeted long-read sequencing, on matched embryonic and adult tissues, of orthologous regions in human and mouse. Altogether 17,931 novel human genes (140,268 novel transcripts) and 22,784 novel mouse genes (136,169 novel transcripts) have been added to the GENCODE catalog representing a 2-fold and 6-fold increase in transcripts, respectively - the greatest increase since the sequencing of the human genome. Novel gene annotations display evolutionary constraints, have well-formed promoter regions, and link to phenotype-associated genetic variants. They greatly enhance the functional interpretability of the human genome, as they help explain millions of previously-mapped “orphan” omics measurements corresponding to transcription start sites, chromatin modifications and transcription factor binding sites. Crucially, our targeted design assigned human-mouse orthologs at a rate beyond previous studies, tripling the number of human disease-associated lncRNAs with mouse orthologs. The expanded and enhanced GENCODE lncRNA annotations mark a critical step towards deciphering the human and mouse genomes.

## Introduction

In 2001, the first drafts of the human genome were published^1,2^. Twenty years later, the complete reference sequence has been finalized, and millions of individual genomes have been sequenced. This data has had an enormous impact on our understanding of human biology and on our capacity to understand and treat disease. However, this data would have been of little value without a reliable map of genes and transcripts, making it nearly impossible to interpret the effect of genetic variation on phenotypes. Early estimates of the number of human genes were highly imprecise: between 30,000 and 40,000, according to the Human Genome Project consortium^2^, and between 27,000 and 39,000, according to Celera Genomics^1^. Shortly after the publication of the first human genome drafts, the ENCyclopedia Of DNA Elements (ENCODE) project^3^ was initiated to identify all elements in the human genome that confer biological function. As part of this broader effort, GENCODE, the encyclopedia of genes and transcripts, was launched to produce a definitive catalog of all human genes and transcripts^4^. Over the years^4–8^, GENCODE, an international partnership of manual annotation, computational biology, and experimental groups has established itself, together with RefSeq^9–12^, as the primary reference gene annotation catalogs.

For the past fifteen years, the number of annotated human protein-coding genes in GENCODE has remained quite stable. The number of long non-coding RNA (lncRNA) genes, in contrast, has been steadily growing since 2007, when they started being systematically annotated^13^, although it appears to be reaching a plateau (Figure S1). Many of these lncRNAs have been shown to participate in important biological functions and to be implicated in diseases. However, while the GENCODE/RefSeq protein-coding gene set has been driven towards convergence by collaborations such as MANE (Matched Annotation from NCBI and EMBL-EBI)^14^ and is believed to be reasonably complete, with no alternative catalogs with the same broad use, this is not the case for lncRNAs^15^. A number of catalogs have been developed over the years, using different approaches and based on different data sets. For instance, FANTOM CAT^16,17^ and CHESS^18,19^, which are based on short-read RNAseq data, annotate a larger number of lncRNA transcripts than GENCODE. GENCODE does not utilize this data due to concerns that short-read connectivity may compromise the fidelity needed to produce reference-quality models^13,20,21^. As a result, despite efforts such as RNA Central^22^, there is a fragmented landscape of non-interoperable and partially redundant lncRNA annotations, which slows down progress in the field^21^. Moreover, in GENCODE and elsewhere, lncRNA transcript annotations are often partial, lacking the correct 5’ or 3’ ends^23^. This hampers their biological characterization; for instance, poor promoter mapping impacts CRISPR screens, while incomplete 3’ UTRs can significantly undermine RNAseq quantification assays.

Thus, during recent phases of GENCODE, we have emphasized producing a reference catalog of full-length lncRNAs. Toward that aim, we have specifically employed the Capture Long-read Sequencing (CLS) strategy^23,24^, and designed a capture array with orthologous probes in the human and mouse genomes targeting different lncRNA annotations and additional regions of these genomes that could also host unknown lncRNAs. We employed a cDNA library preparation protocol, CapTrap-Seq^25^, that enriches for 5’ to 3’ complete RNA molecules. We used this approach in a matched collection of adult and embryonic tissues in human and mouse, selected to maximize transcriptome complexity. The resulting libraries were sequenced pre- and post-capture using PacBio and ONT long-read sequencing technologies.

The manually supervised computational annotation of this data, has led to the inclusion of 17,931 novel human genes (140,268 novel transcripts) and 22,784 novel mouse genes (136,169 novel transcripts) in GENCODE, most of which were identified only post-capture. This is, by far, the largest addition to the GENCODE catalogs since the first drafts of the human and mouse genome sequences, and it constitutes a substantial advance toward a complete lncRNAs catalog in these species. Novel genes and transcripts have features characteristic of “bona fide” genes, including properly formed promoter regions, and strongly differing from those exhibited by matching decoy transcripts. Overall, they are associated with human phenotypes, as supported by GWAS mappings and mammalian sequence conservation. Facilitated by our capture design, we have also produced the most complete lncRNAs orthology map between human and mouse to date. This is essential to investigate the impact of lncRNAs in human biology by modeling their impact in mouse, and indeed, we have found mouse orthologs for many human disease-associated lncRNAs.

Our results underline the crucial importance of a complete gene annotation to fully understand the biology encoded in a species genome. Indeed, we show that the novel genes and transcripts discovered through the CLS approach, greatly enhance the functional interpretability of the human genome, as we can assign to “bona fide” well-constructed transcriptional units and their regulatory regions, millions of previously “orphan” omics measurements (including tens of thousands of CAGE tags, millions of ChIP-seq peaks for hundreds of transcription factors, hundreds of thousands of regulatory regions derived from ChIP-seq of histone modifications, etc.), as well as tens of thousands of “orphan” genetic variants associated with phenotypes.

## Results

### Targeting and sequencing the long non-coding transcriptome with CapTrap-CLS

**FIGURE 1.**
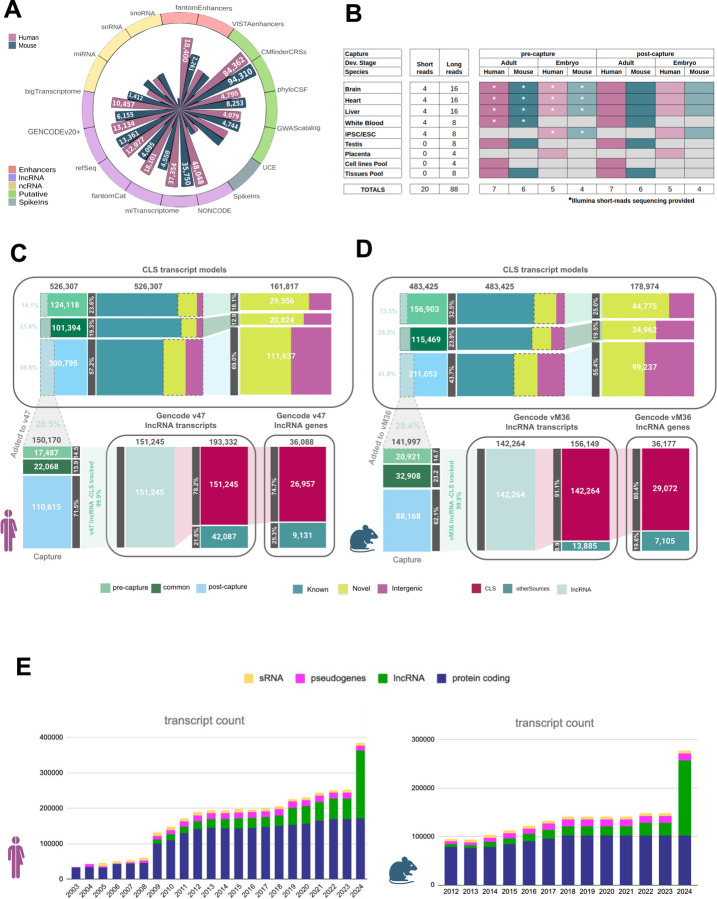
Targeting and sequencing the long non-coding transcriptome with CapTrap-CLS. **A)** Representation of the capture panel; each bar reports the number of targeted regions per catalog, for the human and mouse experiments, organized by the class of elements in focus. **B)** Application of CapTrap-CLS in matched adult and embryonic tissues from human and mouse. Samples were sequenced using long-read platforms from PacBio and Oxford Nanopore Technologies (ONT). Short reads were sequenced with Illumina and highlighted by an asterisk when available. An outline of CLS transcripts and their integration to GENCODE is shown for **C)** human and **D)** mouse. Top panel: final set of CLS transcripts categorized based on the novelty status with respect to GENCODE v27 (human) and vM16 (mouse). Bottom panel: CLS transcript models added to GENCODE v47 (human) and vM36 (mouse) See Figure S6 for a more detailed description **E)** Representation of GENCODE annotation history to releases v47 and vM36 Number of transcripts on primary assembly chromosomes in every year’s last GENCODE release, in human (left) and mouse (right), broken down by broad biotype. IG/TR genes excluded.

We designed a capture array targeting a large fraction of the putative non-coding transcriptome, including the major non-GENCODE lncRNA annotations^13,20,21,23,26–29^, as well as small non-coding RNAs, enhancers^30^, evolutionarily conserved RNA structures^31^, regions that host non-coding GWAS^32,33^, that show putative protein-coding sequence conservation^34^, or that were ultraconserved^35^. Probes were designed in the human genome version GRCh38 using GENCODE v27 as reference annotation (Figure 1A, Table S1). Orthologous regions of the mouse genome were targeted in a corresponding mouse capture library (GENCODE vM16 on GRCm38.p6, Table S2). In total, 176,435 features summing up to 84,103,329 bp were targeted in the human genome (2.9%) and 148,965 features (66,937,555 bp) in the mouse genome (2.5%).

We prepared CapTrap-Seq libraries from a matched collection of adult and embryonic tissues, in human and mouse (i.e., brain, liver, and heart), plus samples designed to maximize transcript complexity. The latter include samples from white blood, testis and placenta, iPSC/ESC cell lines, as well as pools of adult tissues and cell lines. Most samples were sequenced pre- and post-capture using three different technologies: Illumina, PacBio, and ONT. In total, we produced 104 data sets (16 short-read, 438 Million reads, and 88 long-read, 736 Million reads Figures 1B, S2, Table S3). The post-capture samples showed strong enrichment of reads originating from the targeted regions (from 5x to 35x depending on the species, tissue, and platform using the ERCC spike-ins^36^ as a control (Figure S3).

We generated transcript models from the long RNAseq reads using LyRic^37^, an in-house pipeline (Figure S4, Supplement). Models were built for each sample separately, then merged across tissues and stages to produce a comprehensive set of CLS transcript models (Figure S5).

**FIGURE 2.**
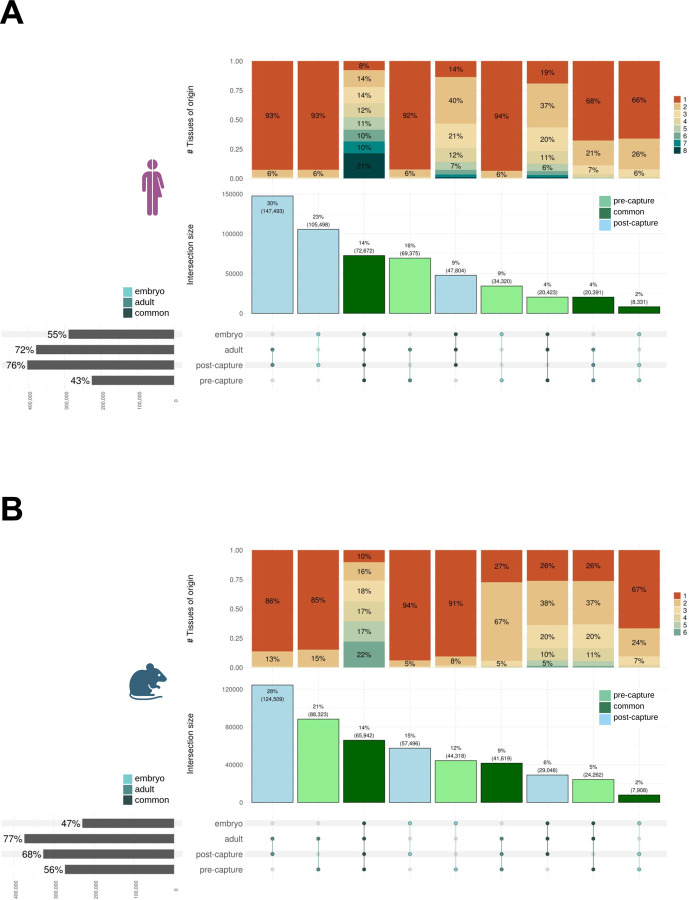
Classification of CLS Transcripts. The panels shows the origin of CLS transcripts in **A)** human and **B)** mouse. The barplot on the left shows the models yield (from top to bottom) pre-capture, post-capture, as well as from adult and embryonic samples (percentage computed over the totality of the transcripts generated). The upset plot shows the intersections across these categories; the dots are colored according to the developmental stage of origin (whether adult, embryo or detected in both), while the bars display the overlap of transcripts between pre-capture and post-capture experiments. The barplot above highlights the proportion of shared transcripts across tissues.

Across all samples, ignoring variations in the transcript termini, we generated 526,307 transcript models in human and 483,425 in mouse (Figures 1C,D, S6, S7A). Of these, 161,817 were novel in human and 178,974 in mouse (with respect to GENCODE versions v27 and vM16, Table S4), predominantly detected uniquely in post-capture samples (78.5% in human and 67% in mouse, Figure 1C,D). Their yield varied across tissues, with testis being the most productive in both human and mouse, followed by brain (both in adult and embryo) in human and the pool of tissues in mouse (Figure S7B). The length of transcripts, exons and introns, the number of exons, the overlap with repetitive elements and other features of novel transcript models are similar to those of lncRNAs annotated in GENCODE (Figure S8, Table S5). Novel transcript models, however, are more tissue-specific than those overlapping known loci: 83% of human and 72% of mouse models are detected uniquely in one sample (compared with 66% and 62% known loci, respectively). Models detected both in adult and embryo tissues are the most broadly expressed (Figures 2, S9).

Finally, different target regions produced different yields of novel transcript models. Overall, about 37% of the targeted regions were detected in human and mouse, the vast majority as a result of the capture (Figure S10). Targets from non-coding RNA annotations, PhyloCSF and enhancer regions were the most productive both in human and in mouse (Figure S11).

### Incorporating CapTrap-CLS models into the GENCODE annotation

The set of CLS transcript models was used as input by the HAVANA team of expert manual annotators to produce GENCODE releases v47 and M36. Manual curation is a founding principle of GENCODE, vital for ensuring the stringency required for ‘reference quality’ gene annotation. Transcript models are constructed by expert annotators, whereby the evidence for every potential model has been traditionally considered on a locus-by-locus basis. Since this approach is not scalable to the one million CLS models produced here, we have deployed a computational workflow, manually supervised at key steps, and developed iteratively via extensive testing by annotators. In order to produce reference quality annotations, its parameters are set to run a minimal false positive rate (i.e., the creation of false annotations) at the expense of an elevated false negative rate (i.e., the rejection of true annotations). This workflow is containerized in a pipeline called TAGENE (Figure S12, Supplement)

As deployed here, we used TAGENE to first eliminate CLS models that are antisense to protein-coding genes or located entirely within the genomic bounds of GENCODE pseudogenes. Next, we examined how annotator confidence in a CLS model correlated with the support for its splice junctions as judged by recount^38^ short-read RNAseq data. Conservatively, we opted to filter out all CLS models that contained any intron supported by less than 50 recount reads (Figure S13), despite the fact that we estimate that ~50% of the models rejected solely on this basis are likely to be correct. Finally, we complemented the TAGENE predictions with manually curated transcripts to help resolve the cases where new models had the potential to merge existing distinct lncRNA genes into single loci.

Using these filters, and additional methodological adjustments, we defined a set of CLS transcripts that meet the stringent requirements to be included in GENCODE. Thus, with this approach we annotated 140,268 and 17,931 new lncRNA transcripts and genes in human, and 136,169 and 22,784 in mouse (with respect to v27 and vM16, respectively), the vast majority incorporated in the latest releases v47 and vM36 (Table S6). This represents a substantial increase over the number of lncRNA annotations present in human GENCODE v46 (>2x) and mouse vM35 (6x, Figure 1E), and brings, for the first time, the number of lncRNA loci in human (37,127) and in mouse (37,336) to comparable numbers. Collectively, these new annotations have increased the transcriptional footprint of GENCODE annotations on the genome by 27.7Mb and 28.9 Mb, respectively (compared to v46 and vM35, Table S7). Transcript models that have not been incorporated into GENCODE for this initial round of annotation may yet be incorporated into future releases as this workflow matures and additional data accumulates to support the inclusion of rejected transcripts.

#### Protein-coding genes/transcripts.

Although this study focuses on lncRNAs, around 100,000 novel CLS models, both in human and mouse, map to known protein-coding genes. These remain to be analyzed systematically by the HAVANA team, but initial inspection suggests some will contain additional coding exons. Here, we have specifically investigated if some of the novel CLS loci actually correspond to previously unknown protein-coding genes. First, we interrogated large-scale tissue-based proteomics experiments. We found convincing evidence for seven human protein-coding genes with multiple peptides that were not annotated as coding. All seven proteins have known human paralogues, and four are substantially truncated compared to their parent gene (Figure S14). Six proteins were detected principally or wholly in testis. For mouse, analysis of proteomics data led to the discovery of 23 protein-coding genes among the novel CLS loci, most of which were also testis expressed (Figure S14C–F). Second, we used PhyloCSF^34^ to find open reading frames (ORFs) within CLS transcripts most likely to contain evolutionarily conserved novel protein-coding regions. Manual examination of over 800 of the top candidates identified one novel protein-coding gene in both human and mouse. (Figure S15). Each of these novel genes has a novel ortholog in the other species. In examining the top candidates, we also discovered other 11 and 47 likely novel protein-coding regions in human and mouse, respectively, including many cassette exons, novel first or last exons, and exon extensions.

#### Pseudogenes.

As in the case of protein-coding genes, our study was not specifically designed to investigate pseudogenes; however, certain regions targeted by probes overlapped with pseudogenes (Figure S10A). In total 5,071 pseudogenes in human, and 2,280 in mouse were targeted by probes (Table S8.1). As expected, given that pseudogenes are generally transcribed at low levels, our capture strategy enhanced the sensitivity in recording expression changes in pseudogenes and their parent genes. Among upregulated pseudogene-parent gene pairs, 1,250 (61%) showed increased expression post-capture in human (Figures S16A,B) and 790 (71%) in mouse. Capturing had a larger impact on the quantification of expression of pseudogenes than of parent genes. Nearly half of the parent genes were not upregulated even when their corresponding pseudogenes were upregulated (Table S8.2, Figure S16C). Still, capturing had an impact on the quantification of parent genes. Over 30% of parent genes were upregulated in human (33% in mouse) after capturing, compared to only 18% (20% in mouse) of non-parent protein-coding genes. These, in contrast, were largely downregulated (Figure S16D,E, Table S8.3). The contrasting behavior between parent and non-parent genes is expected, as protein-coding genes were not targeted in our design, but parent genes share strong sequence similarity with pseudogenes, and could be captured by our design.

#### Unified lncRNA catalog.

**FIGURE 3.**
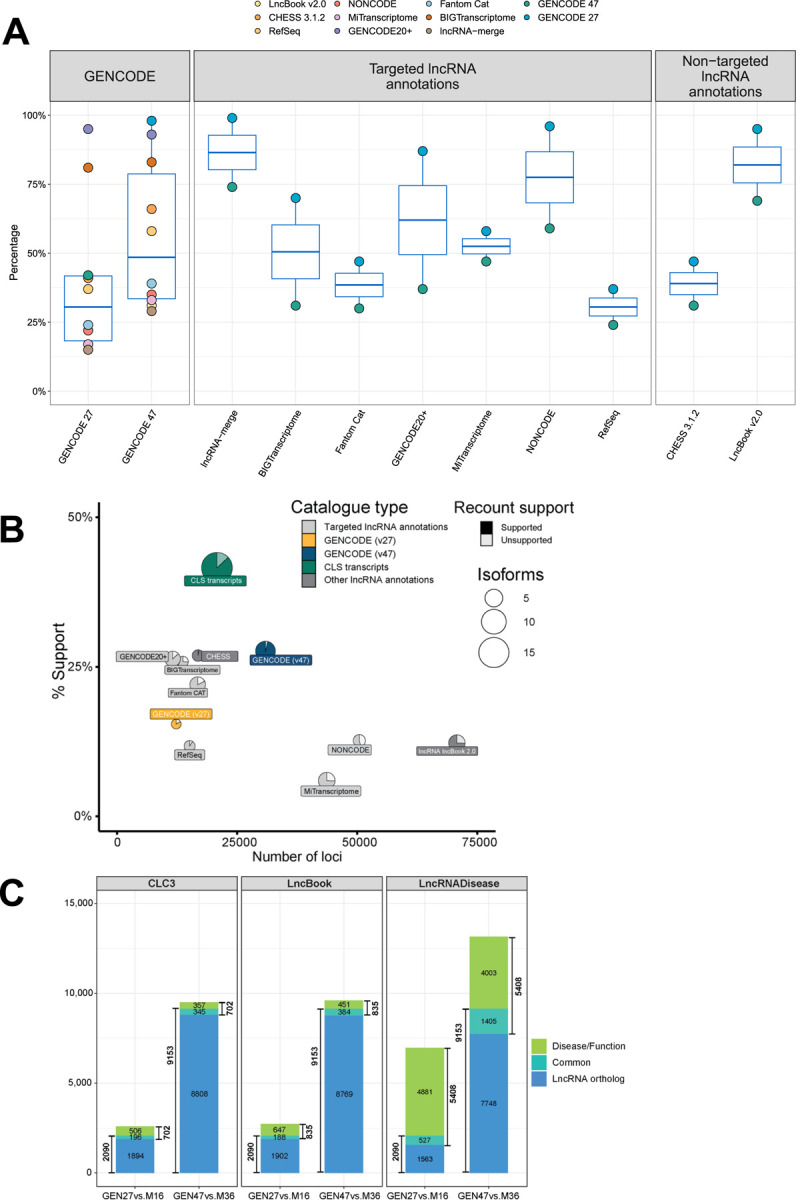
Expansion of the GENCODE lncRNA annotation compared to other lncRNA catalogs. **A)** Gene-level overlap between annotations. The values correspond to the percentage of gene loci from the catalogs represented in the x-axis that overlap the annotations represented in the box-plot. For instance, 29% of the lncRNAs in the merge of all catalogs (lncRNA-merge) are included in GENCODE v47. Conversely, 74% of the lncRNAs in v47 are included in lncRNA-merge. Overlap is defined as a complete overlap of the gene span within either the x-axis set or the corresponding set on the same strand. Both spliced and unspliced genes are included in this analysis. See also Figure S17B. **B)** Comparison of lncRNA catalogs as described in previous study^21^. x-axis: “Comprehensiveness”, representing the total number of gene loci; y-axis: “Support”, indicating the percentage of transcript structures whose start is supported by a FANTOM (Functional Annotation of the Mammalian Genome) CAGE (cap analysis of gene expression) cluster^28^ within ±50 bases, and whose end includes a canonical polyadenylation motif^64^ within 10–50 bp upstream. Circle diameters show “exhaustiveness”, or the average number of transcripts per gene. Pie charts show the proportion of transcripts with all splice junctions supported by recount3 data^38^ (with at least 50 reads). Only spliced models were included in this analysis. CLS transcripts here refer to transcripts identified using CapTrap-CLS, which are spliced, located on the reference chromosomes, and derived from individual lncRNA catalogs. **C)** The overlap between syntenic lncRNA orthologues in human and mouse genomes and the clinically relevant lncRNA genes from three different sources^64–66^.

The driving aim of our work was to advance towards a complete catalog of human and mouse lncRNAs, partially informed by other existing catalogs (Figure S17A). Through the CLS approach, we increased the proportion of lncRNAs from non-GENCODE catalogs from 15% in version v27 to 29% in version v47 (Figures 3A, S17B). The CLS models did not only increase the size of the GENCODE lncRNAs catalog, but also its quality, as reflected across various metrics^21^ (Figure 3B). Despite the inclusion of many models, a significant number of lncRNAs from these catalogs remain absent from GENCODE v47 (77,835 do not overlap annotated loci in v47, Figure S17B). While expanding the range of tissues that we have monitored here is likely to bring in additional candidates, lncRNAs not incorporated into GENCODE are generally weaker compared to those identified through the CLS approach: they have lower recount support (Figure S17A) and they are more catalog specific (Figure S17C).

#### An orthology map of lncRNAs between human and mouse.

Assigning gene orthology between species is crucial for understanding their evolutionary history and, in the case of human, for developing animal models to study and test therapies for human diseases. LncRNAs have been particularly challenging in this regard, due to their lack of sequence conservation, resulting in poor orthology mappings^39,40^. The CLS capture libraries were designed to address this limitation by probing orthologous regions between human and mouse. Coupled with the use of equivalent tissues from both species, this significantly enhanced the sequencing of transcripts from orthologous loci.

We performed orthology-searching between the GENCODE human v47 and mouse vM36 lncRNAs, using ConnectOR, an in-house pipeline, that identifies orthologous lncRNAs based on correct-strand synteny of their exons^41^ (Supplement). We predicted 9,153 human lncRNA orthologues to 9,142 mouse lncRNAs (25% of all lncRNAs in both human and mouse) using a strict reciprocal definition. This is a large increase over the orthologous predicted by ConnectOR between v27 and vM16 (13% and 15%, for human and mouse, respectively, Figure S18A,B). The higher-than-expected number of orthologous lncRNAs, considering the growth of the GENCODE catalog, is likely due to our orthologous probe capture design. The GENCODE catalog of human-mouse lncRNA orthologs is significantly larger than for other available catalogs, which reach up to only 10%^39,40,42^.

The enhanced detection of orthology has significantly increased the identification of clinically relevant lncRNA counterparts in the mouse genome, raising the number of identified lncRNA orthologs in LncRNADisease from 527 (9.7%) to 1,405 (26%), and similarly in other lncRNA disease databases (Figure 3C).

### Enhancing the functional interpretability of the human genome

Accurate gene annotations are fundamental to functionally interpreting the activity (transcription, chromatin modifications, folding, protein binding) and the sequence variation of genomes. Here we show how the GENCODE annotation, extended by incorporation of the CLS data, greatly enhances the functional interpretability of the human genome. In general, we have considered the novel CLS models with respect to GENCODE v27, although some analyses have been restricted to the novel human lncRNA loci in v47, complemented with intergenic spliced CLS models not included in this annotation solely because they did not reach the minimal recount threshold (9,772 genes, 22,211 transcripts). We have compared them to previously annotated lncRNAs (in v27, 8,922 genes, 15,922 transcripts) and protein-coding genes (v27, 19,823 genes, 146,877 transcripts). For some analyses, we have also employed a set of decoy models (17,223 genes, 85,283 transcripts) that attempt to mimic the background (non-genic) behavior of the genome (Supplement). These analyses also serve to validate the biological relevance of the expanded annotation.

#### Transcription Initiation.

**FIGURE 4.**
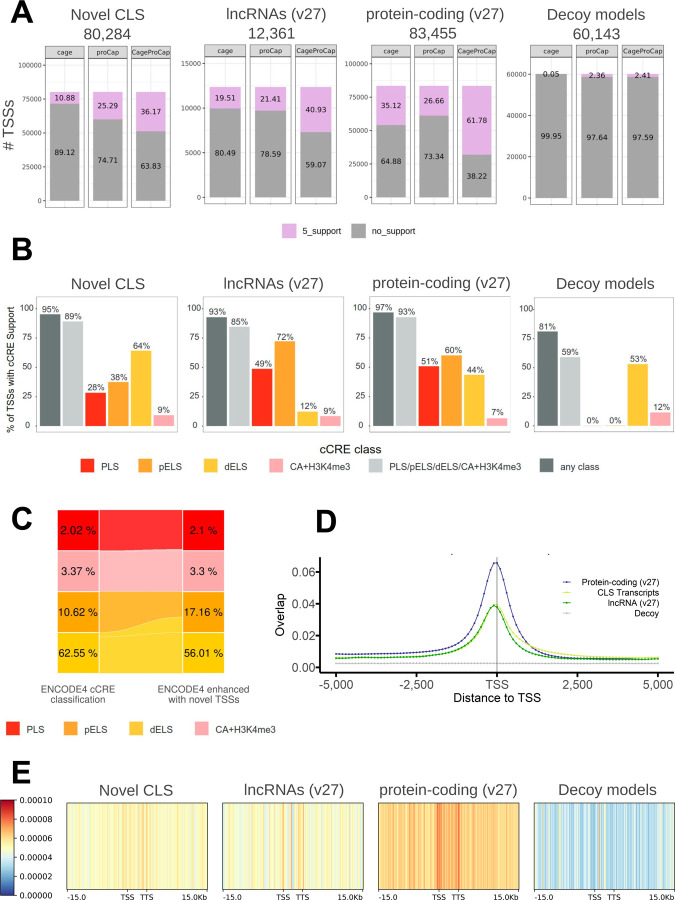
Enhancing the functional interpretability of the human genome. The figure shows how the incorporation of CLS data greatly enhances the functional interpretability of omics measurements on the human genome, assessed on *i)* novel CLS transcripts, *ii)* annotated lncRNA as of GENCODE v27, *iii)* annotated protein-coding genes as of GENCODE v27, and *iv)* decoy models to simulate background signal (from left to right). **A)** Transcription Start Site (TSS) support for novel CLS, annotated lncRNAs, protein-coding and decoy models. Barplots depict the proportion of supported TSSs within each set using CAGE clusters, proCapNet predictions and either CAGE or proCapNet. **B)** Barplot showing the proportion (%, y axis) of Transcription Start Sites (TSSs) supported by different types of cCREs (x axis). TSSs with cCRE support are those for which the distance between the TSS and the center of the cCRE is less than 2 Kb. We performed this analysis for unique TSSs of protein-coding genes, previously annotated lncRNAs, novel CLS transcript models (TM), and decoy models. The type of cCRE is color-coded; “any class” includes additional types of cCREs not shown in the barplot (CA-CTCF, CA-TF, CA, TF). **C)** Alluvial diagram showing the re-classification of TSS-proximity-dependent cCRE categories in the ENCODE registry, given the novel TSS models in the expanded annotation. Two pairs of categories are shown *i)* PLS versus H3K4me3 marking in accessible regions (CA-H3K4me3), and *ii)* pELS versus dELS, which share the same histone marking signatures, but relying on different proximities to closest TSS (200 bp and 2 kb, respectively). The percentages indicate the proportion of cCREs from the entire registry that belong to each category in the original classification (on the left) and upon enhancement with novel TSSs (right). **D)** Peaks of transcription factor binding are centered on TSS of known and CLS transcripts. The plot shows the average (across 1,800 TFs) coverage by ChIP-Atlas peaks of each consecutive 500 bp window around TSS. The coverage increases while we approach the TSS of the real transcripts which is not true for decoys. **E)** GWAS density profile along the gene body and the surrounding ± 15kb area.

In total, we predicted 80,284 novel TSSs in the human genome, 36.2% of which either overlap with CAGE clusters from FANTOM5^16,17^ (n=201,802) or have been associated with ProCapNet predictions^43^, a percentage much larger than for decoys (2.4%). Remarkably, while the CAGE support for annotated lncRNAs and protein-coding genes is much larger than for CLS transcripts, as expected given that these models are mostly seen post-capture, ProCapNet support, which does not directly depend on available sequencing data, is comparable for CLS, lncRNAs and protein-coding genes (Figure 4A). In total we could attach novel TSSs to 10,715 orphan CAGE tags (i.e., tags that could not be associated with previously known TSSs, 7.2% of all orphan tags).

#### Histone Modifications.

Histone modifications are assumed to play an important role in the regulation of gene expression. The ENCODE consortium has recently generated an updated list of 2,348,854 candidate cis-regulatory elements (cCREs) in the human genome after integrating data from 5,712 experiments (DNase-seq, ATAC-seq, ChIP-seq of histone modifications and transcription factors), many of which were performed in the same tissues as those employed in the current study^44^ (manuscript in preparation). About 89% of all novel TSSs were supported by at least one histone mark-associated cCRE. The proportion was similar to the TSSs of protein-coding genes (93%) and previously annotated lncRNAs (85%), but larger than for decoy models (59%) (Figures 4B, S19A,B). However, while known TSSs were mostly supported by proximal cCREs (promoter-like signatures, PLS, and proximal enhancer-like signatures, pELS), novel TSSs were more supported by distal cCREs (distal enhancer-like signatures, dELS), largely reflecting the fact that the ENCODE cCREs classification depends on the proximity to TSSs. Among novel TSSs, support by distal cCREs was particularly strong for tissue-specific TSSs, in contrast to ubiquitously expressed TSSs, suggesting that the latter may co-opt regulatory elements of already known genes (Figure S19B).

We have re-classified cCREs in the ENCODE registry taking into account the enhanced collection of TSSs. As a result, more than 153,000 cCREs previously classified as dELS (about 10%) have been reclassified as pELS, bringing down the proportion of dELS in the human genome from 63% to 56% and increasing, conversely, the proportion of pELS from 11% to 17%. Decoy models, in contrast, produce only a marginal increase (to 13%, Figures 4C, S19C).

#### Transcription factor binding.

Binding of transcription factors (TFs) triggers initiation of transcription. The ChIP-Atlas database^45^ collects ChIP-seq data for more than 1,800 human TFs from about 30,000 experiments. These have been used to generate a set of 64,122,399 unique ChIP-seq peaks along the human genome. We found 89% of the CLS TSSs covered by at least one peak. This number is comparable to that of protein-coding genes (87%) and lncRNA TSSs (82%), and much larger than for decoy models (58%). Similarly, we found overlapping peaks for an average of 121 different TFs for CLS TSSs; a number comparable to protein-coding TSSs (131) and larger than for lncRNA TSSs (76). In decoy TSSs, this average is five. We have additionally computed the fraction covered by ChIP-Atlas peaks of a 500 bp sliding window running −5000 bp to +5000 bp from each TSS region, individually for every TF, and aggregated over all TFs (Table S9, TSS centered window). The density profile, increasing as we approach the TSS, is almost identical for CLS TSSs and lncRNA TSSs, and somehow weaker than for TSSs of protein-coding genes. The profile is flat, as expected, for decoy models (Figure 4D). Overall, the novel CLS transcripts help to assign to promoter regions 2,747,086 ChIP-Atlas peaks (4%) previously mapping to intergenic regions.

#### Non-canonical translation.

LncRNAs are known to host small non-canonical ORFs (ncORFs) that can be translated. Ribo-seq data can typically be employed to assess the translatability of ncORFs^46^. We have used available Ribo-seq data from three human tissues that we have also monitored here (brain, liver and testis^47^) to investigate ncORF translation in the novel CLS transcripts. Overall, we identified 45,198 ncORFs with translation signatures in the CLS transcripts. We found that 27,188 out of 151,611 (18%) CLS transcripts contained one or more translated ncORFs, comparable to annotated lncRNAs (26%), expectedly lower than for protein-coding genes (79%), but much higher than for decoy models (0.02%). The number of translated ncORFs was larger in testis and brain than in liver (Figure S20).

#### GWAS hits.

GWAS allows us to connect variants in the genome with organismic phenotypes. By identifying the genes affected by these variants we can hypothesize the molecular mechanisms underlying organismic traits. We computed the density of GWAS hits from the GWAS catalog^33^ within the boundaries of intergenic CLS transcripts (novel CLS in intergenic regions, Table S10). Overall, we found a density of 4.7 and 10 GWAS hits per 100Kb, within the gene body and within exons, respectively. This is larger than the density in intergenic regions (4.19 hits/100Kb), and comparable to the one within the annotated lncRNAs (4.6 and 8.9 hits/100Kb, respectively). Consistently, the GWAS density within intergenic CLS transcripts is higher than within decoy models (p-value 4.98e-51, Figure S21B). We observed a pattern of GWAS density higher at the boundaries but decaying with distance from the gene body, which is characteristic of annotated genes, also in the intergenic CLS transcripts (Figure 4E). Prior to this study (GENCODE v27), about 65% of the over 262,993 hits in the GWAS catalog mapped within the boundaries of annotated loci. Of the remaining 92,863 GWAS hits, 30,900 (33%) map now within the boundaries of intergenic CLS transcripts; bringing down the percentage of non-genic GWAS from 35% (v27) to 24% (extended v47).

#### Sequence conservation across mammals.

**FIGURE 5.**
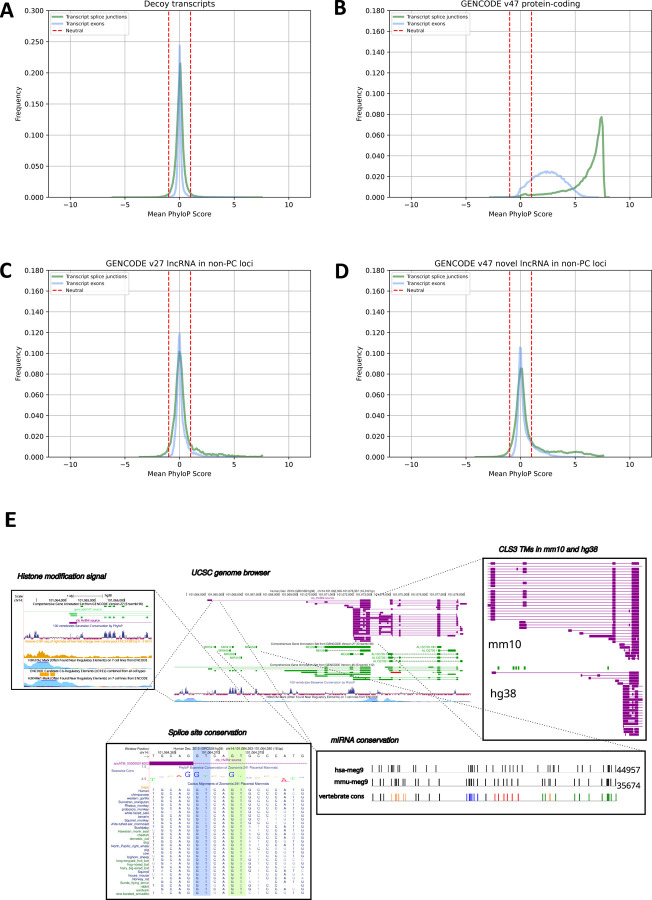
Conservation of lncRNAs and hosting of small RNAs. Frequency of per-transcript exon and splice junction mean PhyloP scores as computed for **A)** GENCODE v47 CLS-based novel lncRNAs outside of protein-coding loci, **B)** GENCODE v27 lncRNAs outside of protein-coding loci **C)** GENCODE v47 protein-coding transcripts, **D)** decoy models. The dashed red lines indicate the range considered under neutral selection. **E)** Example of a putative novel miRNA host gene. The MEG9 locus is a complex ncRNA locus on chr14. MEG9 is highly conserved between mouse and human, with additional exons found in mouse. The microRNA mir-541 cluster and the other miRNAs upstream are present throughout mammals. Given that splicing of the intron is required for miRNA maturation, we find the splice site of the 5’-most exon of the novel lncRNA to be highly conserved across deep mammalian genome alignments (214-way, 470-way). The novel transcript is expressed in liver only, as supported by histone modification marks for H3K27ac.

Conservation of lncRNA sequences is generally low, although some lncRNAs exhibit strong conservation across placental mammals, and even though intron/exon structures can vary, splice junctions often show significant conservation^48^. We evaluated conservation using the Zoonomia 241-way mammalian genomic alignment^49,50^, measuring conservation using the mean per-transcript PhyloP scores^51^ for exon sequences and splice junctions. Decoy models provide a neutral evolution baseline and define the PhyloP scores between −1.0 and 1.0 as neutral for sequence conservation (Figure 5A, Table S11). This results in 97% of the decoy exons being classified as neutral, with 2% showing sequence conservation. GENCODE protein-coding transcripts evaluated using this method show the expected high levels of mammalian conservation (Figure 5B), with 84% of exons and 97% of the splice junctions classified as conserved. LncRNAs outside of protein-coding loci show significantly weaker conservation, although higher than for decoy models (Figure 5C,D). Novel CLS transcripts show higher conservation than previously annotated lncRNAs (16% exons and 26% of splice junctions are classified as conserved in novel CLS transcripts compared to 6% and 14%, respectively, in previously annotated lncRNAs).

#### CLS precursors of small RNAs.

Many classes of small RNAs are processed from long RNAs. For instance, of the 1,869 human miRNAs annotated in GENCODE v27, 1,244 (67%) are contained within annotated long RNAs in the same genomic orientation. Here, we identified an additional 163 orphan miRNAs contained within novel CLS transcripts, bringing the proportion of human miRNAs with reliable precursors to 75% (Tables S12, S13). Notably, the host genes of 35 miRNAs are novel gene loci residing in previously unannotated intergenic space. Figure 5E shows the case of a cluster of orphan miRNAs that fall now within the boundaries of a novel CLS locus. Similarly, we increased the number of mouse miRNAs with potential long precursors from 1,175 (53%) to 1,370 (62%), with 62 orphan miRNAs residing in novel loci. Small nucleolar RNAs (snoRNAs) are another class of small RNAs processed from long precursors. Similarly, we increased the number of snoRNAs with host genes from 572 (61%) to 639 (68%) in human and 535 (36%) to 657 (44%) in mouse, respectively. We found that splice junctions of host lncRNAs, in particular novel CLS transcripts, exhibit much higher conservation than lncRNAs in general (39% of exons and 74% of splice junctions were classified as conserved (Figure S22).

## Discussion

Genes are the basic genomic units responsible for many phenotypic traits. Identifying genes in genome sequences, in particular those from eukaryotic species, is challenging. Genes are often separated from each other by large intergenic regions, they produce multiple transcripts, and the sequence of these transcripts is interrupted by introns, which are removed to produce the mature RNA molecules.

The strong bias in the sequence of the exons and of the splicing signals, and their strong evolutionary conservation and constraint facilitates the identification of protein-coding genes by computational means. Moreover, many protein-coding genes are broadly and/or highly expressed, and transcripts originating from them are well represented in transcriptomic data sets. LncRNAs, in contrast, do not exhibit strong sequence composition biases, and are poorly conserved across the phylogenetic spectrum. Moreover, they tend to be lowly expressed often in a very cell-type specific manner, which makes them difficult to be captured by unbiased sequencing approaches. Therefore, they are poorly represented in transcriptomic data sets. All these make the annotation of lncRNAs particularly challenging.

There is increasing evidence, however, that lncRNAs are more than non-functional by-products of transcription. Growing numbers of carefully documented cases can attest to the bioactivity of mature lncRNA transcripts, and their important roles in both healthy and diseased states^52–54^.

Thus, because of their growing biological relevance, a number of efforts have been dedicated to producing lncRNA catalogs for human and other species^13,20,21,26–29^. Since lncRNAs can essentially be identified only through RNA sequencing, most lncRNA catalogs have been created by processing RNAseq data. This data has been so far largely produced using short-read technology, from which it is difficult to reliably infer exon connectivity along the entire transcript, particularly for lowly expressed transcripts.

As a result, existing lncRNA catalogs contain mostly incomplete and fragmentary models, the biological identity of which cannot be firmly established. They overlap only partially, and the lack of coordination and of consistent accessioning hinders their use by the community, slowing down progress in lncRNA biology. These catalogs are still very useful, however, since they pinpoint those regions in the genome that are likely to encode lncRNAs. Indeed, partially informed by these catalogs, we have employed a targeted RNA sequencing approach that has successfully led to the identification of hundreds of thousands of previously unknown human and mouse lncRNA transcripts, the vast majority of them supported by full-length high-quality sequences.

Our highly stringent filtering results in the exclusion of a substantial fraction of transcripts from GENCODE versions v47 and vM36. For instance, we included only CLS models supported by a minimum of 50 recount reads to each splice junction. This is remarkable, as it indicates that these transcripts have been previously detected in short-read RNAseq experiments, albeit in a fragmented and scattered way (too weak evidence to reliably infer full-length sequences). Thanks to our targeted approach, we have now been able to represent these transcripts as full-length models.

Rare and cell type specific transcripts will likely be underrepresented in bulk RNAseq datasets, therefore many CLS models not reaching our recount threshold are likely to correspond to “bona fide” transcript sequences (we estimate at least 50%, corresponding to tens of thousands of transcripts). Many of these may be incorporated in future GENCODE releases as our manual and automated curation workflows improve. Similarly, about 100,000 CLS models overlapping protein-coding regions were beyond the scope of this study and remain to be analyzed by the HAVANA team. These are likely to lead to many alternatively spliced transcripts and novel translations of existing genes and to extend annotated models to the correct transcript termini.

Transcript discovery and accurate annotation here have been greatly facilitated by advances in long-read RNAseq technologies. However, long-read RNAseq has so far been produced for a limited number of cell types and tissues, mostly in samples from European ancestry^55,56^. Our results suggest that the survey of an increasing number of cell types, conditions, developmental stages, subcellular compartments, and RNA populations (i.e., polyA- RNAs) in samples from individuals of diverse genetic backgrounds, through further advanced long-read sequencing technologies, and library preparation protocols through the single-cell level, is likely to uncover a substantial number of yet unknown human genes.

Deep learning (DL) and large language models seem particularly appropriate to deal with the intrinsic semantic nature of biomolecular sequences, as illustrated by the success in predicting protein structures from amino acid sequences^57^. DL is also being explored to predict genes in genome sequences^58,59^. The accuracy of these methods crucially depends on the size (and the quality) of the training data. Our work, the vast amounts of data produced, and the resulting annotation, can also be considered an important contribution here. The combination of DL with more traditional AI rule-based systems, building on our extensive experience in manual curation, could be the basis of automatic methods producing genome annotations of quasi-manually-curated quality. These methods will be essential to produce high-quality annotations across the entire phylogenetic spectrum^60^, which are essential to maximize the benefit of projects underway to sequence the genomes of all eukaryotic species on Earth^61–63^.

## Supplementary Material

Supplement 1

Supplement 2

Supplement 3
