# Supplementary material for: GENCODE: massively expanding the lncRNA catalog through capture long-read RNA sequencing
Source: bioRxiv. 2024 Oct 31:2024.10.29.620654. Preprint. [Version 1] doi: 10.1101/2024.10.29.620654 (PMC11565817; doi:10.1101/2024.10.29.620654)
Supplement: Supplement 1 [file media-1.pdf]

# GENCODE: massively expanding the lncRNA catalog through capture long-read RNA sequencing

## GENCODE experimental protocols employed to survey the human and mouse transcriptomes

GENCODE has been at the forefront of using advanced methodologies to produce targeted transcriptomic data aimed specifically at gene and transcript annotation. The focus has been to survey the fraction of the transcriptome that is not usually well accessed by standard experimental protocols, complementing therefore the wealth of transcriptomic data produced by the scientific community and deposited in public data archives. The methodologies have evolved through the years towards the automatic production of high-quality full-length transcript sequences that can be incorporated in the GENCODE annotation with minimal manual curation effort. During the pilot phase of ENCODE, extensive experimental validation of annotated transcripts was performed using 5’ RACE and RT-PCR on multiple tissues followed by Sanger sequencing[1]. With the development of tiling arrays, we used their multiplexing capacity to implement the RACEarray normalization strategy, by which 5’ RACE products are hybridized into a tiling array[2]. Subsequently, RT-PCR is carried out from detected exons selected to correspond to novel, not previously annotated, transcripts[3]. Massively parallel sequencing superseded genome-wide tiling arrays, and we implemented RT-PCR-seq, in which RT-PCR products from primers designed to validate exons are pooled and sequenced using Illumina[3]. As soon as long-read sequencing technologies became available, we incorporated them into the GENCODE experimental pipeline. We developed RACE-seq in which RACE products were pooled and sequenced using the ROCHE 454 FLX+ platform[4]. More recently, we implemented the Capture Long-read Sequencing (CLS) strategy, in which probes against targeted regions of the genome are used to capture transcript sequences, which are then sequenced using Pacific Biosciences long-read technology[5]. Over the years, GENCODE has produced, therefore, a unique, massive collection of tens of thousands of targeted transcriptome readouts that have contributed significantly to the quality of the annotation.

## Data production and processing

### Capture design

Short DNA sequences (probes), 120 nucleotides in length, were designed to specifically hybridize with target regions of interest and enrich them using the CLS protocol[5,6]. We designed a capture array with probes targeting a large fraction of the non-coding transcriptome, including virtually all *i)* non-GENCODE lncRNA annotations[5,7–13], as well as *ii)* small non-coding RNAs, *iii)* enhancers[14], *iv)* RNAs predicted to contain evolutionary conserved structures[15], *v)* regions hosting non-coding GWAS hits[16,17], *vi)* showing evolutionary characteristics of protein-coding gene function as

predicted by PhyloCSF[18], or *vii)* being evolutionarily conserved[19]. In addition, we designed probes against GENCODE+[9] catalogs, which is the union of either GENCODE v20 (for human) and GENCODE vM3 (for mouse), with CLS[5] transcript models from the pilot phase. For clarity, here we refer to them as GENCODE20+ and GENCODEM3+, respectively, throughout the text.

Probes were designed in the human genome version hg38 using GENCODE v27 as reference annotation (**Table S1**). Eight key elements in the human genome were lifted over to the mouse genome version mm10, and probes were designed against them; miTranscriptome, fantomCat, bigTranscriptome, CMfinderCRSs, GWAScatalog, UCE, fantomEnhancers, and VISTAenhancers. While for the remaining elements (NONCODE, refSeq, GENCODEM3+, phyloCSF, and small RNAs), probes were designed against the corresponding datasets available for mouse. In total, 176,435 features summing up to 84,103,329 bp have been targeted in the human genome (2.9% of the total ungapped length in hg38), of which 116,383 (66%) intergenic with respect to GENCODE v27 and therefore targeting 54,545,313 bp of the unannotated space. Similarly, 148,965 features have been targeted in the mouse genome, totaling 66,937,555 bp (2.75% of the total ungapped length in mm10), of which 114,926 (77%) intergenic with respect to GENCODE vM16, covering 50,735,573 bp of the unannotated space. In total 107,701 features were orthologous between human and mouse (**Table S2**). To measure the efficiency of our capture protocol, we employed the ERCC spike-ins[20], and designed probes to capture the 42 less abundant (rare) spike-ins (**Figure S3**).

## Library preparation and sequencing

**Ethical statement.** Given that this study utilizes commercial human RNA samples, ethical approval from an institutional review board was not required. However, all procedures were conducted under ethical principles and guidelines for research involving commercial products. Ethical approval for the use of animals in this study was granted by the PRBB animal facility, from which the animals were purchased.

**Samples** (**Figure 1B**). Total RNA was obtained from a diverse set of human and mouse tissues, as well as cell lines. Specifically, samples included 19 human tissues, 4 human cell lines, 20 mouse tissues, and 1 mouse cell line. No biological or technical replicates were included in this study. Detailed information about the samples and their sources can be accessed through the ENCODE portal, where all relevant metadata has been deposited.

For human samples, tissues include adult and embryonic heart, brain, liver, in addition to white blood cells, testis, induced pluripotent stem cells (iPSCs), and placenta. Additionally, a pooled library was prepared from four human cell lines (HCT-116, IMR-90, MCF-7, A549) and a separate pooled library from 13 human tissues: colon, bladder, lung, thyroid, trachea, thymus, esophagus, cervix, adipose tissue, skeletal muscle, spleen, prostate, and small intestine.

Mouse samples were collected from both adult and embryonic mice of the C57BL/6 strain. Tissues include heart, brain, liver, white blood cells, testis, and embryonic stem cells (ESCs). A

pooled library was also prepared from 12 mouse tissues: colon, bladder, lung, thymus, esophagus, kidney, ovary, adipose tissue, skeletal muscle, spleen, prostate, and small intestine.

Total RNA extraction from mouse tissues was performed in-house using TRIzol reagent followed by purification with the PureLink RNA Mini Kit (Thermo Fisher Scientific). As mentioned above, human tissue total RNA was obtained from commercial sources. The purity of RNA samples was evaluated using a NanoDrop One spectrophotometer (Thermo Fisher Scientific), while RNA concentration was measured using the Qubit High Sensitivity RNA Assay Kit (Thermo Fisher Scientific, Cat. No. Q32852). RNA integrity and quality were assessed using RNA Nano chips on an Agilent Bioanalyzer system, with RNA Integrity Number (RIN) values used to confirm sample quality.

**Spike-in controls.** A 1:1 mixture of capped ERCC and Lexogen SIRV spike-in controls was prepared as previously described[21] and added to all samples prior to cDNA library preparation. This mixture served as an internal standard to trace and monitor the sample preparation process.

**Long-Read Library Preparation and Sequencing Protocol.** Total RNA extracted from human and mouse samples was used to prepare double-stranded cDNA libraries following the CapTrap-Seq protocol[21], designated as pre-capture libraries. Sample multiplexing was avoided to prevent demultiplexing problems, and one flow cell was used for each sample. The CapTrap cDNA libraries were then divided into two aliquots to accommodate the requirements of the sequencing platforms. Platform-specific sequencing libraries were subsequently prepared from each aliquot and loaded onto the Oxford Nanopore (ONT) and PacBio long-read sequencing platforms. Sequencing was performed using the respective manufacturing protocols for amplicon sequencing (Amplicon by Ligation SQK-LSK109 for ONT and SMRTbellTM Express Template Prep Kit 2.0 for PacBio). For ONT sequencing, a MinION device with ONT R9.4 flow cells was used, following the standard MinKNOWN protocol script. PacBio data were generated using the Sequel II platform and the standard manufacturing protocol for amplicons sequencing.

Concurrently, another aliquot of 1 µg of cDNA was subjected to a capture experiment using a capture panel, as described in our previously published CLS protocol[5,6]. The resulting enriched libraries are referred to as post-capture libraries. One aliquot of these libraries underwent sequencing using the same long-read sequencing platforms, including ONT and PacBio, and sequencing protocols identical to those used for the pre-capture libraries.

**PacBio Data Preprocessing and Quality Control.** The preprocessing and quality assessment of PacBio sequencing data were conducted externally at Cold Spring Harbor Laboratory (CSHL) in accordance with the manufacturer's protocols. To make the PacBio data compatible with the downstream LyRic processing, PacBio FASTQ files containing CCS (Circular Consensus Sequencing) reads were generated using the pb_gen workflow (https://github.com/guigolab/pb_gen).

**ONT Basecall and Sequencing Quality Control.** Basecalling for ONT sequencing was performed using Guppy v6 SUP, which converts raw signal data from nanopore sequencing into nucleotide sequences. NanoPlot was utilized to generate metrics, including read length distributions, quality scores (Q-scores), and the total number of reads, providing insights into the overall performance of the sequencing run. Additionally, the split_on_adapter utility from the Duplex Tools suite (available on GitHub) was employed to evaluate the presence of concatamers in the ONT sequencing data. Concatamers are formed when multiple DNA fragments are erroneously linked during the library preparation or sequencing process. The split_on_adapter utility from Duplex Tools detects adapter sequences present within the reads, enabling researchers to split them into sub-reads, avoiding downstream misalignments. This utility was adjusted in accordance with the CapTrap-Seq adapter and primer sequences. In addition to these changes, two rounds of "read splitting" were performed. In the first step, the complete set including the ONT adapter linked to the CapTrap-Seq primer was used to detect concatemers and split them into sub-reads. Due to presence of incomplete ONT adapter within some reads, these concatemers were left undetected. Therefore, a second round of splitting was performed wherein the splitting was based on presence of only the CapTrap-Seq adapter within the reads. Later, as a final step of this quality control, the multi-split reads either reported by the utility or split in both the consecutive rounds of splitting were discarded. Overall, the sequencing data was preprocessed to meet the necessary quality standards for reliable downstream analysis.

Raw PacBio and ONT reads are available through ArrayExpress accession E-MTAB-14562.

**Short-Read Library Preparation and Sequencing Protocol** We generated matched short-read RNA-seq data using total RNA and the SMARTer Stranded Total RNA-Seq Kit following the manufacturer's protocol. All samples were barcoded and multiplexed using the Illumina barcoding system (6 mers), and then sequenced in a HiSeq 2500 lane with HiSeq Sequencing v4 Chemistry. On average, 27 million 125-base paired-end reads were generated for each sample. Paired-end reads were subsequently quality-checked and processed together with long-reads.

Raw reads are available through ArrayExpress accession E-MTAB-14562.

## Sequencing Statistics

Overall, we get a consistent mapping rate around 99%, with differences in sequencing statistics across samples mostly driven by the sequencing technology of choice. As expected, regardless of the species, tissue, and capture design, samples sequenced with ONT display a higher throughput (measured as number of sequenced reads) with respect to their counterpart sequenced with PacBio, which on the other hand is associated with a systematically longer mapped read length (**Table S3**, **Figure S2**). Across all samples, ONT gives rise to a substantially higher number of models with respect to PacBio, which is related to the throughput of the technology but does not correlate with the number of sequenced reads. Similarly to what is reported for mapped read length, the transcript models originated from PacBio are, on average, consistently longer than those generated with ONT (**Table S3**).

## LyRic

To streamline the analysis of raw reads obtained from CapTrap-Seq experiments and enhance the discovery of novel transcripts while reducing dependence on the pre-existing reference annotations, we have developed LyRic[22]. LyRic is a snakemake-based bioinformatic pipeline that automates the identification of full-length transcripts from long-read RNA-seq data (**Figure S4**) and incorporates quality control at every step. It is a conservative pipeline, combining low false positives rate with high sensitivity in detecting novel transcript models, as shown in the LRGASP project[23]. First, LyRic maps long-reads to the reference genome using minimap2[24]. In the subsequent steps, LyRic performs a filtering process to remove poor-quality alignments. It achieves this by identifying the High-Confidence Genome Mappings (HCGMs) which consist of only canonical and high-quality sequence splice junctions for spliced reads. This helps eliminate spurious introns that may arise from RT template switching. For unspliced reads, HCGMs require the presence of a detectable, clipped polyA tail. If short-read RNAseq data is available, LyRic performs an additional filtering step on the spliced HCGMs to generate high-quality Hi-Seq-Supported read mappings (HiSS). Importantly, LyRic includes unspliced HCGMs with a polyA tail in the transcript model-building process, even though it does not calculate short-read support for them. When short-read data is not utilized to support spliced HCGMs, as in the work presented here, HiSS reads are equivalent to HCGMs in terms of representation.

The identified HCGMs (or HiSS, if generated) serve as the basis for constructing transcript models using the *tmerge* software[25]. With *tmerge*, compatible reads and transcripts are merged to create non-redundant transcript models. Notably, spliced and unspliced reads are treated separately and never merged together. To ensure the reliability of the resulting transcript models, *tmerge* incorporates parameters that prevent artificial elongation of intron chains. Additionally, LyRic addresses the issue of mismapped splice junctions by correcting exon/intron overhangs. Finally, through the *buildLoci* software[26], LyRic can be employed to merge the newly obtained transcript models into sets of overlapping transcripts, i.e., loci. Additionally, this utility enables the redefinition of gene boundaries in existing gene annotations based on the overlap across transcripts.

## Spike-ins and capture efficiency

After mapping all reads to the 92 ERCC spike-ins, wherein 42 target spike-ins with varying abundances were selected after removal of the 8 most abundant ones, we observed a 40-fold increase in the proportion of reads mapping to the rare spike-ins post-capture compared to pre-capture (from 2.5% to 96%) (**Figure S3**). This substantial fold increase was consistent across various tissues and sequencing platforms, underscoring the robustness of our capture method. In total, between 7% and 39% of all reads in humans and between 9% and 58% of all reads in mice depending on tissues and sequencing platforms, mapped to targeted regions in the post-capture samples (**Figure S3**). This variation highlights the influence of biological and technical factors on capture efficiency. Specifically, the enrichment of reads mapping to CLS-targeted regions demonstrated a substantial increase across different sequencing technologies. For PacBio sequencing, the fold increase ranged from 6 to 15 in human samples, and from 9 to 35 in

mouse samples. Similarly, for ONT, the fold increase varied from 4 to 9 in human samples, and from 8 to 27 in mouse samples (**Figure S3**).

## Building CLS models

The CLS long-read data was processed with LyRic to obtain high-confidence transcript models per sample. Models yielded across tissues, stages and technologies were anchored according to the 5’ and 3’ support assigned by LyRic, and merged together to build a comprehensive set for this experiment (**Figure S5**). This way we reduce the redundancy present across samples, while preserving potential alternative, tissue-specific, transcriptional start and end sites. The initial set of transcripts was manually refined to identify and tag potential artifacts (see TAGENE filters below). Accordingly, we filter out those models sharing same-strand exonic overlaps with annotated pseudogenes, as well as opposite strand mismapping to annotated genes (see TAGENE filters 3 and 4), resulting in the exclusion of 9,120 and 10,754 transcripts in human and mouse, respectively. Additionally, we kept track of all the transcripts originally built from any proportion of reads having a contrasting polyA tail and canonical splice junctions strand assignment.

As an outcome, we identified 1,212,480 unique transcript models in human and 1,092,208 in mouse, which corresponded to 526,307 unique intron chains and monoexonic models in human, and 483,425 in mouse (see section on intron chains below). As an initial quality check, the accuracy and quality of the models have been assessed using SQANTI3[27], in comparison to GENCODE v27 and vM16, for human and mouse, respectively [https://guigolab.github.io/CLS3_GENCODE/SQANTI_reports/Human_CLStranscripts_v27.html, https://guigolab.github.io/CLS3_GENCODE/SQANTI_reports/Mouse_CLStranscripts_vM16.html ]. A huge fraction of those models are built from ONT reads (80% and 78%, respectively in human and mouse), as well as more than a half have been contributed post-capture (63% and 56%, respectively in human and mouse). The higher fraction of models detected in adult tissues with respect to embryo ones (64% and 67%, respectively in human and mouse), is mainly attributable to the higher number of adult tissues employed. About 11% of the models are detected by both ONT and PacBio in the two species, which translates into 35% of the PacBio and 14% of the ONT models in human, and 34% of the PacBio and 15% of the ONT models in mouse. On the other hand, 8.5% of all the models are commonly detected pre- and post-capture (19% of pre-capture and 13% of post-capture models), while 9.6% are shared in mouse (18% and 17% pre- and post-capture, respectively). The majority of the transcript models (about 95%) originate from one or two tissues only; models detected across sequencing technologies, developmental stages, and capture strategies are also more broadly detected across tissues. This relatively low overlap can be partially explained by the still very-high proportion of redundant intron-chains (see below). Indeed, by anchoring 5’ and 3’ supported ends in the attempt to preserve potentially interesting alternative TSSs and TTSs, we are intrinsically preserving highly similar transcript models with only slight differences at the extremities. This issue is tackled by generating, per species, a catalog of unique intron chains, collapsing together all those transcript models sharing the same

exon-intron structure, keeping the furthest 5’ and 3’ extremities as TSS and TTS, respectively (see intron chain section below).

The utility used for anchor-merging is available at https://github.com/guigolab/LyRic/blob/master/utils/anchorTranscriptsEnds.pl

## Novel Transcript models

To assess the novelty harbored by the aforementioned set of anchored transcript models, we compared their structures to the GENCODE v27 and vM16 reference annotations by means of gffcompare[28], and assigned them to nine simplified categories based on the extent of their overlap, further grouped in two disjoint classes; known, and novel (**Table S4**). Of all transcript models, 262,843 were novel in human and 303,996 in mouse; the larger number of novel mouse models is likely attributable to the less advanced state of the annotation compared to human. Of these, 93,425 mapped to regions implicitly annotated as intergenic in human and 131,618 as intergenic in mouse, respectively, corresponding to 17,911 and 25,936 completely novel loci (see loci section below).

## Intron chains

To further reduce the redundancy across the different models, those were collapsed into unique intron chains, simply merging the transcripts sharing the same internal exon-intron structure (as identified by gffcompare[28]), ignoring eventual variation at the terminal exons, and therefore neglecting end-support information. A different strategy was employed for mono-exonic transcripts, which were extended into a single one when overlapping more than 50% of their length, again irrespective of the end support. Different tissues produced different yields of models, with testis being among the most productive in both human and mouse, followed by brain (both in adult and embryo) in human and the pool of tissues in mouse (**Figure S7**).

The separate projection of spliced and monoexonic transcript models resulted in a set of 468,598 unique intron chains and 57,709 monoexonic regions in human (**Figure S6A**), and a total of 407,194 unique intron chains and 76,231 unspliced regions in mouse (**Figure S6B**). In the main manuscript we use the term “CLS transcript models” to refer to the union of unique intron chains and monoexonic transcripts (526,307 in human and 483,425 in mouse). Overall, 161,817 of such transcript models were novel in human (with respect to GENCODE v27) and 178,974 in mouse (with respect to vM16), of which 62,734 and 79,777 mapped to intergenic regions, respectively, corresponding to 17,911 and 25,936 novel loci (**Figures S6, S7B**). The majority of these novel CLS models were spliced (71% in human and 64% in mouse). Spliced models were highly supported by independent recount[29] reads; about 75% have all splice junctions supported by a recount score higher than 50 in human, and 78.7% in mouse (**Figure S13**).

The extent of agreement between the two technologies now increases, which makes the set of models yield through PacBio almost a subset of those gained through ONT (**Figure S9BC**). More precisely, in human, 25.5% of the transcript models are now shared between the two technologies

(28% and 76% of the entire ONT and PacBio sets, respectively), while 28.3% are shared in mouse (31% and 74% of the entire ONT and PacBio sets, respectively). The overlap also increases when comparing the transcript models detected pre- and post-capture; in human, 76.4% of the models are detected post-capture, 19.3% are shared across design (45% and 25% of the total number of models obtained pre- and post-capture, respectively). Similarly, 67.5% of the transcript models are detected post-capture in mouse, and overall 24% are shared across pre- and post-capture samples (42% and 35% of the total number obtained pre- and post-capture, respectively).

The consensus of the models across different tissues also increases, with about 20% of the transcripts detected in three or more tissues, in accordance with the increase of consensus across sequencing technologies, developmental stages, and capture strategies (**Figure S9BC**). Among all the regions targeted by this experiment, 37% in human and 36% in mouse eventually detected transcription (**Figure S10B**). Of these, in human, 28.6% help detect 107,981 completely novel transcript chains (**Figure S11C**), representing 66.7% of all novel transcript chains (**Figure S11A**). While for mouse, 29.6% of the regions helped detect 105,399 completely novel transcript chains, representing 58.9% of all novel transcript chains (**Figures S10, S11B**,**D**), proving the effectiveness of the capture in detecting novel transcripts. With respect to the total number of regions targeted, 25.5% and 20.7% of the targeted regions, respectively in human and mouse, were detected uniquely post-capture (**Figure S10B**), further highlighting the efficiency and the importance of the capture for refining and detecting these poorly annotated regions.

### Loci

With the sole intent of grouping together different models in uniquely identifiable loci, we clustered CLS transcripts into regions of continuous transcription. For this purpose, upon merging all the transcripts together using *tmerge* to further reduce any redundancy, we employed bedtools[30] to test for overlap across them. Eventually, transcripts sharing any overlap on the same strand have been brought together into a single locus using *buildLoci*, preserving their structure. An additional round of intersection with the reference annotation (GENCODE v27 for human, and GENCODE vM16 for mouse) was carried to track whether the newly identified loci overlied previously annotated genes, and assign novelty at the loci level accordingly.

## Incorporating CapTrap-CLS models into the GENCODE annotation

### The TAGENE Pipeline

As a reference annotation resource, GENCODE has strict criteria for the inclusion of models into its geneset, and these must evolve when new technologies become available to support annotations. Over time, the sequencing quality of long-read data has improved, alongside methodological advances in the algorithms used to process and map the data. Nonetheless, GENCODE does not incorporate aligned transcriptomics data directly into the geneset, i.e., using an unsupervised, computational approach. This is because reference gene annotation strives towards perfection in terms of the quality of transcript models included. Thus, it is generally

considered that the inclusion of incorrect models, e.g., those that do not accurately represent genuine biological transcripts, is more harmful to the geneset than the omission of correct models. In other words, in this context, reference annotation takes a conservative approach.

Historically, the accuracy of the reference annotation has been ensured by the fact that all GENCODE models are constructed manually by expert human annotators, who examine the 'evidence' for each prospective model (e.g., transcriptomics data) in the context of the genome sequence. For the first decade of the GENCODE project, human and mouse annotations were built almost entirely using cDNAs and EST sequences deposited in INSDC databases, the number of which proved to be tractable for a purely manual approach, which was deployed locus-by-locus, chromosome-by-chromosome.

Today, the primary challenge provided by long-read datasets to reference gene annotation is in terms of the sheer volume of reads produced. These numbers are not tractable for GENCODE manual annotation, as previously performed. However, we consider that full manual annotation is in fact not necessary for long read datasets. Primarily, this is because the size of next generation datasets, including short read RNA-seq libraries, provide key information leveraged to support the annotation process. Fundamentally, the most important aspect is confidence in the structure of a transcript model, i.e., its splice junctions, start, and end points. Historically, the need to individually check each of these elements of a prospective model was a major time burden on the manual annotation process. It is now well established that splice junction accuracy can be controlled, at least to some extent, in model construction through the use of short read data to supplement long-read alignments. However, prior to our work here, it was not apparent how this should be achieved to create reference gene annotations.

Thus, in order to maintain reference annotation standards for the incorporation of CLS data, it was first necessary to fully understand the quality of the aligned reads produced by LyRic. At the same time, given the size of these datasets, we needed to devise a pathway for the large-scale incorporation of these reads into GENCODE that was not dependent on manual annotation, and yet nonetheless could achieve high stringency. The combination of these two strands evolved into the creation of the TAGENE annotation workflow.

Principles for the design of TAGENE

TAGENE, as far as possible, has been designed to replicate the manual annotation process in terms of its logistical stages. However, the process is greatly simplified as we are not attempting to annotate coding sequences, given that the scope of this phase of the project is entirely focused on the annotation of lncRNAs. Thus, in creating GENCODE gene models, TAGENE needs to accomplish several things: *i)* ensure that models have accurate splice junctions, *ii)* set appropriate first and last coordinates for each model, i.e., start and end points, which would ideally correspond to transcription start sites (TSSs) and polyadenylation (polyA) sites as discussed below, and *iii*) ensure that 'merging' behavior is appropriate, i.e., the process by which two or more overlapping models may or may not be ultimately joined together into a single GENCODE model. To ensure that TAGENE works in line with the principles of manual annotation, our solution was to develop

an iterative workflow based on substantial input from the expert annotators in the HAVANA group, which were fed back to the development team to make adjustments for the next run.

### Manual assessment of the splicing accuracy of CapTrap-Seq CLS transcripts model

Splice junction accuracy is crucial to the overall quality and consistency of the GENCODE geneset; we take a conservative approach to the assessment of introns for inclusion where only canonical splice sites supported by contiguous alignment of transcriptional data are allowed. Thus, the HAVANA group at EMBL-EBI performed a manual assessment of the quality of splice junctions contained in CLS reads aligned through LyRic. At this point we were specifically interested in examining introns that had low expression, based on the consideration that this set was more likely to include false splicing reactions. For all aligned reads produced by LyRic, we first produced scores for all introns. Specifically, we measured the level of expression of each splicing reaction (as opposed to the expression of the overall transcript) using short-read data processed by the recount3 project. Next, 10 aligned read introns for each recount3 intron score between 0 and 199 were randomly selected to create a set of 2000 introns for manual checking. Each intron was assessed for accuracy based on standard GENCODE annotation guidelines, and independently of any other evidence. Introns identified as being incorrect, i.e., not annotatable by GENCODE, were QC'd a second time by a second annotator. Where annotators were satisfied that an intron was incorrect, we attempted to extrapolate a reason for this outcome. This allowed for us to classify false introns into several categories, outlined below, and then used to devise additional filtering stages for TAGENE. To ensure the high stringency of reference annotation reads were screened for *i)* recount3 score, *ii)* tandem repeats overlap, *iii*) pseudogenes overlap, *iv)* opposite strand mismapping to coding genes, and *v)* splice sites misalignments.

Of the 165 incorrectly spliced introns identified, 115 had a recount3 score below 50. The majority fell into one of the "incorrect alignment" categories previously defined. It was therefore decided to set a conservative recount3 intron score threshold of 50, attempting at removing the majority of the observed alignment errors. We estimate that approximately 50% of introns with a score below 50 are in fact likely correct, although this proportion is inappropriately low to support reference annotation. Further, we identified 14 examples in which the splice junction of a CLS read was localized within a genomic tandem repeat, as defined by Tandem Repeats Finder. In short, tandem repeats are defined as two or more adjacent copies of a sequence of nucleotides. In these cases, we found that it was impossible to unambiguously determine the true splicing structure of the CLS read as it aligned equally well to multiple locations. As such, we decided to filter out all TAGENE models that contain a splice junction localized within a tandem repeat. In 50 cases CLS reads incorrectly aligned at or near a pre-existing GENCODE processed pseudogene due to the alignment of the read polyA tail to the genomic polyA sequence inserted at retrotransposition, ultimately conferring a higher mapping score to the pseudogene locus over the parent protein-coding gene. For this reason, we decided to filter out all TAGENE models having same-strand exonic overlap with annotated pseudogenes. We note that this was a brute-force approach, and that substantial numbers of TAGENE models overlapping pseudogenes are likely genuine. We plan to revisit this question in future iterations. We also found 20 examples in which LyRic misaligned a CLS read onto the opposite strand of a protein-coding gene. It was determined that

these reads genuinely belonged to the protein-coding gene, but were incorrectly placed due to the pipeline's alignment software being unable to find canonical splice sites on the true strand. This was in turn caused by the poor sequence quality of the underlying reads, and was therefore decided to filter out all TAGENE models antisense to annotated genes, when the first and final coordinate of such models fell within a window defined by the gene boundaries of the existing locus. Finally, 57 cases were identified where the lack of sequence similarity between the CLS read and the genomic sequence, particularly around potential splice sites, led to ambiguous alignments deemed by a GENCODE annotator to be resolvable to an alternative, better supported, splice site. In these cases we suspect that the underlying RNA-seq support from the recount3 dataset likely fell victim to the same misalignment issue.

### Merging CapTrap-Seq CLS transcripts models into the existing GENCODE annotation

The GENCODE geneset (v46) already contained a rich and highly detailed catalog of lncRNA annotations (20,310 loci, 59,927 transcripts). The new CapTrap-Seq CLS transcripts therefore needed to be integrated into this existing annotation in a logical and consistent manner, and in accordance with the current GENCODE manual annotation guidelines. All novel CLS transcripts sharing exonic overlap and mapping to an intergenic region were assigned to the same new lncRNA locus and provided with a unique, stable Ensembl locus-level ID (ENSG ID) and Ensembl transcript-level IDs (ENST IDs). CLS transcripts with exonic overlap to a single pre-existing GENCODE lncRNA were automatically designated as part of the existing locus and given new Ensembl transcript-level stable IDs. Where CLS transcripts shared exonic overlap with two or more pre-existing lncRNA loci, GENCODE expert human annotators provided manual oversight to determine whether they should be merged into a single locus or maintained as separate genes. If the loci were merged the CLS transcripts were automatically added to the new single gene, otherwise manual supervision was required. The decision-making was carried in accordance to the current GENCODE manual annotation guidelines, evaluating the presence of transcriptional data to support TSSs, polyA sites, and consistent use of the proposed full-length intron chains as determined via RNA-seq intron counts from the recount3 dataset. The maintenance of previously well defined lncRNA genes was prioritized by manually supervising the addition of CLS transcripts to lncRNAs with known gene names/symbols. Additionally we consulted with the HUGO Gene Nomenclature Committee (HGNC) at the University of Cambridge with regard to all cases in which lncRNAs with known gene names/symbols were to be merged, to determine which gene name/symbol should be retained.

## Protein-coding genes/transcripts

To determine which protein-coding genes had already been added to GENCODE due to CLS data, we searched for GENCODE v43 protein-coding transcripts whose CDS was entirely contained in a CLS transcript and for which some portion was not annotated as transcribed in GENCODE v27; GENCODE manual annotators then examined the annotation notes for each such gene to determine if CLS data was what had prompted its addition. We found that CLS data

has already led to the addition of at least three novel human protein-coding genes, namely *NFILZ*, *RPSA2*, and T-cell receptor ENSG00000289723.

**PhyloCSF search.** To find CLS transcripts likely to contain additional conserved novel protein-coding regions not previously annotated we searched for every complete open reading frame (ORF) in a CLS transcript, at least 25 codons long, some portion of which was *i)* not annotated as protein-coding or pseudogene in GENCODE v43 or vM32 or *ii)* antisense to a protein-coding region or pseudogene, at least 15 nucleotides of which were not annotated as transcribed in v27 or vM16, having a positive PhyloCSF score and positive PhyloCSF-Psi score, whose alignment had relative branch length at least 0.4, and for which there was no species in which some portion of the alignment overlapped the alignment of an annotated coding region (which usually indicates a pseudogene). PhyloCSF was run using the `58mammals` parameters on alignments extracted from the 58 placental mammal subset of the hg38 100 vertebrates whole genome alignment (for human) or the 40 placental mammal subset of the mm10 60 vertebrates whole genome alignment (for mouse), downloaded from the UCSC Genome Browser. We then manually examined the resulting candidates using CodAlignView (https://data.broadinstitute.org/compbio1/cav.php) to find regions likely to be novel coding regions.

Manual examination of over 800 top candidates in human and mouse identified one novel protein-coding gene in each. The human one has a 24 codon ORF having hg38 coordinates chr3:117729172-117729179+chr3:117997182-117997248(-). The mouse one has two isoforms, a 37 codon ORF having mm10 coordinates chr6:136171799-136171878+chr6:136172263-136172296(-), and a 16 codon ORF having coordinates chr6:136171861-136171882+chr6:136172268-136172296(-). Each of these novel genes has a novel ortholog in the other species.

In examining the top candidates, we also discovered 11 and 47 other likely novel protein-coding regions in human and mouse, respectively, including many cassette exons, novel first or last exons, and exon extensions. We expect that a more thorough examination of CLS transcripts overlapping known protein-coding genes by GENCODE's expert manual curators will reveal many more novel protein-coding regions.

**Proteomic Search**. Proteomic evidence for the translation of the CLS data came from large-scale mass spectrometry analyses. In the case of the human CLS data, the translated CLS regions and the human reference protein sequences annotated in GENCODE v43 were mapped to spectra from four large-scale, tissue-based, proteomics experiments[31–34], while for mouse we mapped the CLS translations and the GENCODE vM32 reference protein sequences to spectra from a single compendium of normal tissue experiments[35]. Spectra from the five experiments were downloaded from ProteomeXchange[36].

Peptide-spectrum matches (PSMs) were generated with COMET[37] using default parameters, including a maximum precursor charge of 4, a maximum fragment charge of 3 and a mass tolerance of 10.0. Peptides were limited to a minimum length of 7 and a maximum length of 40

amino acids. We allowed the oxidation of methionines. The PSMs detected by COMET were post-processed with Percolator[38]. We used the default parameters in Percolator too, including setting the test and training false discovery rate to 0.01. We considered only fully tryptic peptides with up to two missed cleavages and PSM that had Percolator posterior error probabilities (PEP) values below 0.0002. These filters meant that the false discovery rate of the novel peptides from the CLS data was 1.41% for the human CLS data (0.25% at the PSM level) and 2.04% in the mouse analysis (0.28% for PSMs).

We further disregarded peptides that were just a single amino acid different from annotated protein sequences because these identifications might also be explained as single amino acid variants or post-translational modifications of the annotated protein sequences. To validate the translation of a CLS ORF, we required at least two non-overlapping peptides.

We found convincing evidence for seven human novel protein-coding genes with multiple peptides that were not annotated as coding in the Ensembl/GENCODE reference set. All seven proteins have known human paralogues, and four are substantially truncated compared to the parent gene (**Figure S14**). Six proteins were detected principally or wholly in testis. For example, *C5orf60* is annotated as non-coding in both Ensembl/GENCODE and RefSeq, but we detected ten peptides for the protein in our analysis. We detected peptides for the correct *WASHC1* gene that has recently been uncovered as part of the novel T2T-CHM13 assembly[39,40]. For mouse, analysis of proteomics data led to the discovery of 23 protein-coding genes that were novel to GENCODE, most of which were also testis expressed (**Figure S14**). As with the human proteins, the coding status of many of these ORFs was already predicted by other projects (mostly RefSeq in the case of mouse), with GENCODE having previously considered many of them to be pseudogenes. None of the novel coding genes for which we detected peptide evidence overlapped with the PhyloCSF-supported ORFs.

## Pseudogenes

We used featureCount[41] to quantify expression at the CLS loci level. The loci were tagged with same-strand annotation overlaps to GENCODE v43 for the human and GENCODE vM16 for the mouse. The following parameters were applied: -L -s 0 -T 8 -t exon -g gene_id. In total, we quantified 74,073 genes (CLS loci) for human and 88,192 for mouse across all tissues and cell lines. On average, 98.75% of alignments were successfully assigned to a feature.

For downstream analysis, we mapped gene IDs from the CLS model to the gene IDs in the GENCODE annotation, using bedtools intersect[30] with a requirement of at least a 1 bp overlap. Next, we categorized protein-coding genes into two groups: parent and non-parent, based on the parent protein-coding gene identified by PseudoPipe[42] (**Table S8.4** for human and **Table S8.5** for mouse). Using the mapping relationship from CLS model to GENCODE, we established gene IDs for pseudogenes and their parent genes in the CLS model.

We then analyzed gene expression patterns across various experimental conditions using DESeq2[43]. The experiments involved three variables: *i)* whether the sample was captured, *ii)* the sequencing technology used, and *iii)* the tissue origin of the sample. To assess the individual effects of capturing and DNA sequencing technology, we controlled for the remaining two variables as covariates. We defined significantly differentially expressed genes as those with an absolute log2 fold change greater than 1 and FDR-adjusted p-values < 0.001.

In the main manuscript, we analyzed the impact of capturing in the quantification of expression of pseudogenes and parent genes. We have also analyzed the impact of the sequencing platform. For both the human and the mouse, we observed that more pseudogenes were upregulated using ONT compared to PacBio. In contrast, more parent genes showed upregulation with PacBio (**Figure S16A**).

## The unified lncRNA catalog for human and mouse genomes

The primary goal of this work was to expand the GENCODE lncRNA catalog by integrating various lncRNA annotations with GENCODE (**Figure S17A**). All targeted annotations are detailed in [9], and for clarity, GENCODE+ is referred to as GENCODE20+ in this text. Most of the targeted annotations rely on GENCODE, while others, like NONCODE[44], are integrative, combining manual literature searches with other annotations. This leads to significant redundancy across individual catalogs, as shown by their gene-level overlap (**Figure S17B**). To account for variability across annotations, we generated gene loci for each catalog (except GENCODE v27 and v47) using the *buildLoci* utility[26]. To create a non-redundant lncRNA catalog (lncRNA-merge), transcript models from all targeted lncRNA annotations were merged using *tmerge*[25], ran using the default parameters with the exception of *--exonOverhangTolerance*, which was set to 8. Notably, *tmerge* handles spliced and monoexonic transcripts separately, as monoexonic transcripts are never merged. Finally, gene loci boundaries for the lncRNA-merge catalog were generated using the *buildLoci* software[26]. The gene-level overlap was calculated using *bedtools intersect*[30], run with default parameters along with the additional options -f 1, -F 1, -e and -s. The quality comparison of GENCODE v47 with other leading lncRNA annotations was performed following the approach described in previous work[9], with two key modifications. First, "completeness" (y-axis) has been replaced with "support" because FANTOM (Functional Annotation of the Mammalian Genome) CAGE (cap analysis of gene expression) clusters are derived from independent experiments, whereas the evaluation of 3' end completeness relies on the proximity of a canonical polyadenylation signal[45]. Consequently, the presence or absence of CAGE/polyA support does not directly determine transcript completeness. In this analysis, a transcript is considered supported if its start overlaps a robust phase 1/2 FANTOM CAGE cluster[11] (n = 201,802) within ±50 bases, and its 3' end contains a canonical polyadenylation motif[45] within 10–50 bp upstream. Second, to evaluate the accuracy of lncRNA transcript structures, we introduced a new layer represented by pie charts. These display the proportion of transcripts with all splice junctions supported by recount3 data[29], with a minimum of 50 reads per splice junction.

The analysis of catalog specificity was conducted by examining the efficiency of intron chain merging. Transcripts with distinct intron chains were considered non-redundant and, therefore, catalog-specific. For each catalog-specific lncRNA transcript not included in GENCODE v47, we identified and reported its source catalog. As expected, the majority of unannotated catalog-specific lncRNAs originated from automated annotations such as NONCODE or MiTranscriptome (**Figure S17C**).

## LncRNA orthology map between human and mouse

LncRNA orthology prediction was performed using an updated version[46] of the ConnectOR pipeline[47]. ConnectOR operates by performing a LiftOver of syntenic regions between the human (hg38) and mouse (mm10/mm39) genomes, in both directions. ConnectOR was run with default parameters using exon mode to produce strand-specific orthology predictions. Instances where results were classified as “not lifted” or “one to none” were interpreted as no orthology prediction.

The negative control set was generated using an in-house script, *shuffleGTF*, which randomizes gene positions along the genome while preserving their exonic structures. We ran *shuffleGTF* 100 times for each comparison to obtain a distribution of values, and the median value was used to calculate the false discovery rate. The false discovery rate for lncRNA orthology predictions is approximately 3% for v27 and vM16, and around 6% for GENCODE versions v47 and vM36 (**Figure S18B**).

The utility used to generate the control set is available at; https://github.com/cobRNA/utilities

## Enhancing the functional interpretability of the human genome

### Extended GENCODE v47

While thousands of the CLS transcripts were incorporated into GENCODE v47, a fraction of those were left out as they failed to meet the initial requirements of the TAGENE workflow (see above). However, despite these criteria being meant to ensure high stringency of reference annotation, few thousands of spliced CLS transcripts ended up being discarded a priori, of which roughly a thousand remain intergenic with respect to GENCODE v47 annotation. Of these, 88% were discarded solely because not matching the minimum recount3[29] score threshold (more than 50 reads coverage at the lowest supported junction). As deemed still potentially reliable, and to evaluate their biological relevance, for some analyses we employ an extended version of the current GENCODE v47, complemented with the set of aforementioned CLS transcripts, clustered into loci (1,066 genes, 1,039 transcripts).

### Decoy models

With the objective of building a set of matched transcripts against which to assess the biological relevance of our results, we generated a set of decoy models by randomly relocating the spliced

CLS loci structures (n=45,819) in the intergenic space. For this purpose, using bedtools 2.29.2[30,48], we merged the GENCODE annotation v27 with our generated set of spliced CLS loci, extended the boundaries of each entry 10kb on both sides, then subtracting those coordinates from the reference chromosomes (GRCh38/hg38, excluding the mitochondrial genome). From this newly defined intergenic space were removed the ENCODE blacklist regions (ENCFF356LFX) and the centromeres[49], as well as other genomic gaps retrieved via the UCSC table browser (group 'Mapping and Sequencing', track 'Gap', table 'gap', format 'bed'). For each CLS locus, drawn at random, the set of associated spliced transcripts was relocated in one of the aforementioned intergenic chunks chosen at random, ensuring no overlap among them. Loci for which those requirements could not be fulfilled were excluded. Strand information was not propagated. Eventually, we obtain 17,223 random loci (85,283 spliced transcripts), covering around 250 million bases (70.2% of the viable intergenic space).

### TSS support

Sets of non-redundant representative TSSs were built for *i)* lncRNAs (v27), *ii)* novel CLS transcripts, *iii)* protein-coding genes (v27) and *iv)* decoy models. Herein, the consecutive TSSs within 100bp from each other were merged and associated to a single representative TSS, chosen to be the median among the collapsed ones. Specifically to generate the set for novel CLS, their TSSs (extended 100 bp) were intersected to GENCODE v27 (184,093 TSSs) to get a set of novel TSSs that do not overlap those already annotated. Further, these novel TSSs were merged to give a non-redundant set of 80,284 novel TSSs.

### Support by Histone modifications

We utilized the ENCODE4 cCRE tissue-agnostic catalog to evaluate the extent to which the transcription start sites (TSSs) of our novel CLS models are supported by external epigenetic evidence. In this context, cCREs support is defined by a proximity of less than 2 kb between a given TSS and the center of a cCRE. Given the stronger overlap of novel TSSs with distal cCREs (dELS) compared to previously annotated TSSs (**Figure 4B**), we explored how this enrichment correlates with the expression levels of these novel TSSs across the different tissues. To investigate this, we classified novel TSSs into two main groups: ubiquitously and non-ubiquitously expressed. Within the non-ubiquitously expressed group, we further identified tissue-specific TSSs. For each TSS, we gathered all the corresponding CLS models and recorded the tissues in which they were expressed, without distinguishing between adult and embryonic samples. A TSS is classified as ubiquitously expressed if its associated CLS models are expressed across all tissues. If the CLS models are expressed in only a subset of tissues, the TSS is classified as non-ubiquitously expressed. Within this latter case, if all CLS models associated with a given TSS are expressed in just one tissue, the TSS is defined as tissue-specific. From this latter set, TSSs uniquely expressed in tissue-pool or cell-pool samples were excluded from these analyses, as it is not possible to determine which specific tissue or cell type contributed to their detection. A similar proportion of ubiquitously expressed and non-ubiquitously expressed TSSs are supported by cCREs (96% and 95%, respectively). However, while the former are mostly associated with proximal (PLS and pELS) cCREs, the latter are predominantly supported by distal (dELS) cCREs

(**Figure S19A**). This enrichment in distal regulatory activity was even stronger among tissue-specific TSSs: 69% of these TSSs, on average across tissues, showed support by at least one dELS cCRE (**Figure S19A**). We also evaluated whether the dELS cCREs intersecting tissue-specific TSSs showed activity in the same tissue as the corresponding CLS model. For this analysis, we focused on three adult tissues (heart, liver, and testis) with available tissue-specific cCRE catalogs and maps of H3K27ac and H3K4me3 histone modifications[50]. On average across the three tissues, 11% of the intersecting dELS cCREs showed activity (i.e., were not classified as "low DNase") in the same tissue as the corresponding TSS. This proportion reached an average of ~49% (53% heart, 63% liver, and 30% testis) after integrating peaks of H3K27ac and H3K4me3 histone modifications (**Figure S19B**).

## Transcription factors binding

The ChIP-Atlas 3.0 database[51] integrates 428,000 ChIP-seq, ATAC-seq and Bisulfite-seq experiments from six species. We downloaded the summary (**fileList.tab**) file from the ChIP-Atlas portal. We filtered this file for the experiments that contain "hg38" in the genome assembly (second) column and "TFs and others" in the class (third) column. We next filter the type (fourth) column by the valid name of human transcription factors and the cell type (sixth column) for "All cell types". Thus we obtained a list of 1,800 human transcription factors for which there is at least one ChIP-seq experiment in ChIP-Atlas. For each transcription factor we downloaded the full collection of peaks across all tissues at FDR < 1e-5 (q=5) threshold. We merged the peaks for the same TF and obtained 64,122,399 peaks in total (35,624 on average per TF). Peaks for different TFs may overlap each other.

## ncORF

Ribo-seq datasets were obtained from Wang et al.[32], which included three samples of testis, three of liver and three of brain. Ribo-seq data was processed through an in-house pipeline and translated sequences were identified with RibORF v2.0[52] with default threshold considered (0.6), at least 10 reads per sequence detected, and found in at least one of the three samples per tissue.

## GWAS analysis

We pruned the NHGRI-EBI GWAS catalog[53] (downloaded on 24 October 2023) down to 134,059 unique coordinates, using a greedy approach similar to what previously described at Boix et al.[54]; upon ranking hits by significance, we iteratively collapsed SNPs within a 5 kb window, with a top-down approach, assigning each pruned entry to its representative signal, to ensure an unbiased calculation of GWAS density/100kb. Additionally, all associations falling within the HLA locus (for hg38, chr6: 29,723,340 - 33,087,199) were discarded. Bedtools 2.29.2[30] was employed to intersect the whole catalog against *i)* intergenic CLS transcript models, *ii)* the set of randomly generated decoy models, the reference annotation GENCODE v27, further split into *iii)* protein-coding genes, and *iv)* lncRNAs which overlap with protein-coding genes no longer than 10% of their length (n = 8,922), *vi)* the intergenic space according to GENCODE v27 and *vii)* to GENCODE v47. For the purpose of the current investigation, the genomic space has been restricted to reference chromosomes only (mitochondrial excluded), refined as previously

described for decoy models. With the term “intergenic CLS transcript models” in the current analysis we refer to all those models used to build intergenic lncRNAs genes now in GENCODE v47 (30,247 CLS transcripts), together with those spliced CLS transcripts belonging to loci still intergenic with respect to GENCODE v47, originally excluded because failing the recount3 score filter (1,066 genes, 1,309 transcripts). The analysis has been conducted at transcript and exonic level, separately. The GWAS density/100kb has been then computed dividing, per each obtained set, the total number of unique hits after mapping to their representative proxy, by the total genomic area spanned;

$$( \#Pruned\_Hits / Genomic\_Length ) * 100{,}000$$

This has been further refined grouping captured CLS models by targeted catalog, in the attempt to dissect different trends associated to the different nature of the targeted elements. Finally, the significance of our results was assessed by means of pairwise t-tests on the density of GWAS per transcript computed across *i)* the set of intergenic CLS models with respect to GENCODE v27, *ii)* the set of randomly generated decoy models, *iii)* the protein-coding annotated genes, and *iv)* the annotated lncRNAs refined as reported above. Finally, GWAS density in the ±15kb region flanking gene bodies has been computed with deeptools v3.5.2[55] using a bin size of 200 bp, and setting the TSS to TTS window size to 5kb.

Overall, considering the catalog before pruning, 15,406 out of the 92,863 (16.6%) GWAS hits not enclosed in GENCODE v27 map now within the boundaries of intergenic CLS loci; number that raises up to 30,900 (33%) if we were to consider all the CLS nucleotides not overlapping with the annotation, bringing down the percentage of non-genic GWAS from 35% to 29% and 24%, respectively. When dissecting the contribution of the different targeted catalogs, models originating from phyloCSF, fantomEnhancers, and regions predicted to encode structural RNAs, are among the ones showing the highest density of GWAS hits per 100Kb (irrespectively of whether whole gene body or exonic space were considered). Remarkably, these are the type of elements in which single nucleotide disruptions are likely to have the larger functional impact (**Figure S21A**).

Sequence conservation across mammals

We used mammalian conservation of human lncRNAs as a metric to compare the GENCODE CLS-based lncRNA annotations with previously existing lncRNAs. We obtained PhyloP scores for the Zoonomia 241-way mammalian alignment from the UCSC Genome Browser[56]. To evaluate conservation, we employed two methods: per-transcript and per-unique intron. For the per-transcript metric, we computed mean exon and splice-junction PhyloP scores. We used the mean score of four splice junctions for the per-unique intron evaluation.

Transcripts with more than 10% of the exon bases not having PhyloP scores or having any of its splice junction bases lacking scores are excluded. Similarly, all unique intron splice junction bases must have scores to be included.

Each method has its trade-offs and biases. The per-transcript analysis counts bases multiple times, while the unique intron method may still involve overlapping donor or acceptor sites and ignores the overall transcript context. Since we aimed to compare the nature of GENCODE annotations rather than examine precise conservation levels, we chose these straightforward methods, focusing primarily on the per-transcript analysis. Also, the single alignment column nature of PhyloP scores of splice junctions does not consider donor or acceptor sites that may have moved location in the orthologous RNAs, although the intron is conserved. Examples of moved splice sites can be seen in white-faced saki, tamarin, and white-tufted-ear marmoset in MEG9 (**Figure 5E**).

To avoid confounding conservation signals from protein-coding genes, we separately analyzed lncRNAs overlapping protein-coding loci. For GENCODE v47, we only considered novel lncRNAs based on CLS LyRic models. In the conservation analysis of miRNA and snoRNA host genes, we classified them based on the same-strand genomic overlap from either the transcript or the unique intron. GENCODE v27 and v47 annotations are evaluated independently. The annotations within each GENCODE release determine the classification of protein-coding loci and host RNAs for that release.

**Figure 5** shows the distribution of scores from both metrics. We set the neutrally evolving range based on the PhyloP score distribution from -1.0 to 1.0. This threshold resulted in 97% of the per-transcript exons and 91% of the per-transcript introns being conserved. The distribution of decoy scores for unique introns was broader, with 84% falling within the neutral range. The breakdown of the transcript categories examined and the frequencies of accelerated, neutral, and conserved PhyloP scores are given in **Table S11**.

Remarkably, lncRNAs hosting miRNAs and snoRNAs are significantly more conserved than lncRNAs in general, both for previously annotated hosts in v27 (22% of the exons and 58% of the splice junctions), and with stronger conservation for the novel CLS hosts (22% of exons and 65% of splice junctions, **Figure S22**).

### Long transcripts act as hosts of orphan small RNAs.

**The small RNA set** comprises GENCODE genes of gene types miRNA, snRNA, snoRNA, scaRNA, sRNA and Mt_tRNA, as well as tRNAs from a complementary GENCODE annotation file. The number of small RNAs has remained stable since v27 and vM16, respectively, with only minor changes (**Table S12, S13**).

**The long RNA set** contains the remainder, i.e., all but small RNAs described above, with protein-coding genes, lncRNAs and pseudogenes being the most prominent gene types (**Table S12, S13**).

**Genome partitions.** To assess the position of small RNAs and their putative host genes in the genome, we partitioned the genome into genic and intergenic regions, and the genic partition

further into exonic and intronic. This way, we assigned a unique label to each nucleotide in the genome in a hierarchical fashion (gene over intergenic, exon over intron). Partitions were created both for annotations on the same strand, as well as independent of their strand. The former were used to identify host transcript regions to exclude any influence on genome composition and conservation by genes on the opposite strand. Using this procedure we produced partitions for all 'long RNAs' (see long RNA set above), for all 'protein-coding genes' (gene types: protein_coding, polymorphic_pseudogene, translated_processed_pseudogene, all IG_genes, TR_genes and their pseudogenes) and for 'CLS transcripts'.

**Intergenic space.** The definition of intergenic regions in this study varies slightly depending on the goals of the individual analysis. When studying the small RNA-host gene relationships, intergenic is defined as the space between long genes on the same strand, because miRNAs and snoRNA are co-transcribed with their hosts and post-transcriptionally processed from mostly introns. Orphan small RNAs lacking a host are small RNAs in intergenic space, for many of which we were able to identify novel host genes. Studying conservation of splice sites in lncRNAs we excluded protein-coding genes to avoid any sequence conservation signal interfering. We therefore defined intergenic/non-protein-coding space as the intervals between protein-coding genes (definition see above), not considering their strand.

**Resulting gene sets.** By comparing the positions of small RNAs (see above), long RNAs (see above), intergenic space and CLS loci, we created new subsets, for which we propose biological relations and functions. MicroRNAs and snoRNAs, for instance, reside in introns of host genes, i.e., lncRNAs or protein-coding genes. They are transcribed along with their hosts in an operon-like fashion and are subsequently processed from introns. Host genes therefore require miRNAs and snoRNAs to be *i)* contained in introns and *ii)* on the same strand. Small RNAs that do not fulfill these criteria were labeled as 'orphan'. In human, 1,244 (67%) of 1,869 miRNAs annotated in GENCODE v27 have host genes, while 625 (33%) are orphans, whereof 397 (21%) are considered true orphans, i.e., they do not even overlap with a long transcript in antisense. In mouse, 1,175 (53%) of 2,207 microRNAs annotated in GENCODE vM16 have host genes, while 1,032 (47%) are orphans, whereof 700 (32%) are true orphans in intergenic regions (IG) (**Table S12, S13**).

**Discovering novel host genes.** For 16-41% of IG orphans we were able to identify potential host genes in our CLS. In humans, we found host genes for 163 (41%) of 397 IG orphan microRNAs, where 35 (9%) reside in novel CLS loci and 128 (32%) in extensions of annotated genes. For snoRNAs in human, the success rate was slightly lower: 67 (30%) of 220 IG orphan snoRNAs, 16 (7%) in novel CLS loci, 51 (23%) extensions of annotation. In mouse, we identified CLS hosts for 195 (28%) of 700 IG orphan miRNAs (62/9% novel CLS loci) and for 122 (16%) of 760 IG orphan snoRNAs (45% in novel CLS loci, **Table S14, S15**).

## Data and Code availability

Docker image with reference R packages used for analysis can be pulled at docker://tamaraperteghella/r4_gencode_phase3.

The codes, data, and links to the original files can be found at; https://github.com/guigolab/CLS3_GENCODE, doi: 10.5281/zenodo.13941033.

# Supplementary Figures Captions:

**Figure S1. GENCODE annotation history.** Numbers of genes and transcripts on primary assembly chromosomes in every year's last GENCODE release in human **(A, B)** and mouse **(C, D)** broken down by broad biotype. IG/TR genes excluded.

**Figure S2. Mapped read length distribution.** Distribution of reads' length upon mapping, excluding reads mapping to SIRVs and ERCCs. Each sample is shown separately for human (left panel) and mouse (right panel). For visualization purposes the x axis has been cut at 3,500bp.

**Figure S3. Capture on spike-ins. A)** Spiked-in synthetic External RNA Control Consortium (ERCC) RNA sequences targeted in the capture design. Read enrichment for the **B)** targeted control ERCC sequences, and **C)** targeted regions across all catalogs post-capture, in both pacBio and ONT samples aggregated across tissues and developmental stages. **D)** Read enrichment for the targeted regions in pacBio and ONT post-capture samples in each tissue and developmental stage.

**Figure S4. LyRic workflow.** The LyRic pipeline workflow for long-read RNA-seq data analysis. The process includes seven main steps: (1) Read alignment using Minimap2[24] (long reads) and STAR[57] (short reads, if available); (2) Identification of High-Confidence Genome Mappings (HCGMs) and HiSeq-supported stranded reads; (3) Orientation based on splice sites and poly(A) tails; (4) Merging of stranded alignments into non-redundant transcript models using *tmerge*[25]; (5) Evaluation of transcript completeness at the 3' and 5' ends using polyA tail clipping and external CAGE data[58,59]; (6) Optional customization steps, where non-overlapping capture-targeted regions for each sample are provided in standard GTF format to group features and generate summary statistics; (7) Generation of per-sample GTF files containing the final transcript models.

**Figure S5. CLS transcript model creation workflow.** High-confidence models obtained for each sample through LyRic were subjected to "anchored" merging across all tissues and developmental stages to obtain a comprehensive set of transcripts. These transcripts were further merged together into unique "intron chains" and monoexonic transcripts, then clustered together based on same-strand exonic overlap into CLS loci.

**Figure S6. CLS transcript model summary.** A detailed graphical classification for the obtained CLS transcript models (top-right panel) as well as those added to GENCODE (bottom-right panel) in **A)** human and **B)** mouse. The left-bottom panel shows the proportion of GENCODE lncRNA genes and transcripts either refined or incorporated thanks to CLS transcripts.

**Figure S7. CLS transcript models yield across experiments.** Barplots (left) display **A)** all; and **B)** novel CLS transcripts obtained across all samples, pre-capture, post-capture, and commonly detected in the two. The three panels on the right show (from left to right) the number of CLS

transcripts obtained across both pacBio and ONT, as well as individually through pacBio and ONT sequencing platforms.

**Figure S8. CLS transcripts and exons length distribution.** From left to right, for **A)** human and **B)** mouse, the plots display the distribution of transcripts length, exons length and introns length, compared across all CLS transcripts, novel CLS transcripts, annotated lncRNAs, and protein-coding transcripts. The central panel is split in two rows, showing the length of all exons (top) and the length of the internal exons (bottom).

**Figure S9. Classification of CLS anchored transcripts. A)** Extent of tissue sharing across CLS transcripts in human (left panel) and mouse (right panel), grouped by novelty status as described in Table S4. The CLS transcripts distribution according to several metrics across the experiment is shown for **A)** human and **B)** mouse. The barplot on the left shows the models yield (from top to bottom) for ONT, PacBio, in pre-capture and post-capture, as well as for adult and embryonic samples (percentage computed over the totality of the transcripts generated). The intersections across these categories are summarized by the upset plot; the dots are colored according to the technology of origin (whether unique to ONT, unique to PacBio, or detected through both), while the bars display the overlap of transcripts between pre-capture and post-capture experiments. The barplot above highlights, for each intersection, the proportion of shared transcripts across tissues.

**Figure S10. Target regions detected. A)** Proportion of probed target regions detected and their biotype distribution with respect to GENCODE v27 and GENCODE vM16 for human and mouse. **B)** Proportion of probed target regions detected pre-capture, post-capture, commonly in pre-capture and post-capture and across all the experiments in human (top) and mouse (bottom). **C)** Proportion of target regions that help detect novel and known CLS transcripts (GENCODE v27 in human; top and GENCODE vM16 in mouse; bottom)

**Figure S11. Novel CLS transcript models captured.** Matrix depicting the number of novel CLS transcripts overlapping the probed target regions across each feature type and sample, as well as collated across samples and all the catalogs, in addition to the novel transcripts that did not have any overlapping probes in **A)** human and **B)** mouse. The corresponding proportions are also reported for both in **C)** human and **D)** mouse.

**Figure S12. The TAGENE workflow.** Overview of the TAGENE workflow to integrate CLS3 long reads into the GENCODE annotation. Long RNA-seq reads are subjected to stringent filtering to remove possible misalignments, especially those introducing spurious splice sites, and merged into transcript models having unique intron chains. This TAGENE transcript set is then compared to the HAVANA annotation, which is the union of the current GENCODE annotation and recently added manual annotation, in order to determine what TAGENE transcript models will introduce new exon sequences or splice junctions. Finally, this selected subset of the TAGENE transcripts is merged into the HAVANA annotation, which can involve the creation of novel transcripts or the extension of existing transcripts, in what will be part of the new GENCODE annotation release.

**Figure S13. Recount support.** Proportion of CLS transcripts (y-axis) supported by increasing recount3[29] score support (x-axis) for human (left) and mouse (right).

**Figure S14. Possible coding regions for which we detected at least two non-overlapping peptides for transcripts from in the human and mouse CLS analyses.** Structures predicted using the HHPRED server[60] or AlphaFold3[61]. The detected peptides are mapped to the structures In yellow. **A)** Human predicted pseudogene MFFP3 which is expressed in testis and N-terminally truncated with respect to its parent. The ORF is also present in mouse, but it is even more truncated and would be a single helical protein. **B)** Human predicted pseudogene CFAP144P1, detected in testis and sperm and in higher quantities than the parent gene. **C)** A Smg5-like ORF in mouse with peptides detected in nervous tissues. It is substantially different from the parent gene, but it conserves important functional residues - a Smg5 ligand (shown in red) has been mapped onto the model and the Smg5-like residues that would bind this ligand are highly conserved. **D)** Mouse Taf7l2, annotated as lncRNA in GENCODE and peptides detected in testis and epididymis. **E)** The globular domain of a mouse Mageb4-like protein, peptides detected in testis. **F)** The N-terminal domain of a mouse Ankrd26-like protein, peptides detected in testis and epididymis. We detected peptides for a novel Ankrd26-like protein in human too, but it is not clear whether the two genes are related[61].

**Figure S15. Novel 2-transcript protein-coding mouse gene found by searching CLS transcripts for regions with high PhyloCSF score that were not already annotated as protein-coding.** The 37 and 16 amino acid transcripts share most of the first exon and overlap in different frames in the second exon. The human ortholog is also a novel protein-coding gene (not shown) but it is not contained in any CLS transcript. **A)** UCSC Genome Browser image showing the two transcripts, overlapping CLS transcripts, and PhyloCSF signal indicating evolutionary signature of conserved protein-coding DNA. **B)** and **C)** Mammal genome alignments of the two transcripts, rendered to show features indicative of protein-coding evolution, including frame conservation and predominance of synonymous substitutions (light green), by CodAlignView (https://data.broadinstitute.org/compbio1/cav.php)

**Figure S16. Comparison of the effect of sequencing technology and capturing on expression levels of pseudogenes and parent protein-coding genes. A)** Heat map plot indicating the number of pseudogenes and parent protein-coding genes that were upregulated when comparing the ONT and PacBio sequencing. **B)** Pairs of pseudogenes and parent protein-coding genes that were upregulated in pre-capture or post-capture. **C)** Number of expressed pseudogenes and parent protein-coding genes in various tissues depending on the sequencing and capturing technology. **D)** and **E)** Bar charts indicating the proportion of significantly upregulated or downregulated parent and non-parent protein-coding genes when comparing **D)** ONT and PacBio sequencing and **E)** pre-capture vs post-capture.

**Figure S17. Targeting lncRNA catalogs. A)** Number of transcripts from individual catalogs targeted by the CapTrap-CLS approach and incorporated into GENCODE v47. The unique

transcripts represent the non-redundant set of transcript models across all individual annotations. The percentage at the top of each bar indicates the proportion of transcripts from each catalog with all splice junctions supported by recount3 data[29] (at least 50 reads per individual splice junction); **B)** Gene-level overlap between annotations, using a strict definition. The values represent the percentage of gene loci in each row's annotation that overlap with those in each column. Overlap is defined as a complete overlap of at least one gene's span on the same strand. Both mono- and multi-exonic genes are included in this analysis. **C)** Catalog-specificity of lncRNA transcripts incorporated to GENCODE v47 (25.4% shared across catalogs; 74.6% catalog specific) and those not detected by the CapTrap-CLS experiment (4.2% and 95.8% respectively). The catalog composition represents the source of 95.8% catalog-specific transcripts that were not included in the GENCODE v47 annotation.

**Figure S18. Detecting positionally conserved lncRNAs between human and mouse genomes. A)** The number of orthologues in each orthology class. *One-to-half* are species-specific orthologs (one-way), that cannot be verified by reciprocal definition; **B)** Orthology predictions using negative controls generated through the shuffle GTF approach[46].

**Figure S19. cCREs support for novel CLS TSSs. A)** Barplot showing the proportion (%, y axis) of TSSs of novel CLS models supported by different types of cCREs (x axis), distinguishing between ubiquitously expressed and non-ubiquitously expressed TSSs (y axis). The type of cCRE is color-coded; "any class" includes additional types of cCREs not shown in the barplot (CA-CTCF, CA-TF, CA, TF). In the lower panel, a similar representation focuses on tissue-specific TSSs across the five different tissues. **B)** Barplot showing the proportion (%, y-axis) of dELS cCREs intersecting tissue-specific TMs that are characterized by chromatin activity in the same tissue as the corresponding TSS. "Active cCRE" means that the cCRE was attributed a category different than "low-DNase" in the corresponding tissue, "H3K4me3" and "H3K27ac" means that the TSS of the TM was found within 2 Kb from a peak of H3K4me3 or H3K27ac in the corresponding tissue. "Any support" is the union of active cCREs, H3K4me3, and H3K27ac-supported TSSs. **C)** Alluvial diagram showing the re-classification of TSS-proximity-dependent cCRE categories in the ENCODE registry. Two pairs of categories are shown *i)* PLS versus H3K4me3 marking in accessible regions (CA-H3K4me3), and *ii)* pELS versus dELS, which share the same histone marking signatures, but relying on different proximities to closest TSS (200 bp and 2 kb, respectively). The percentages indicate the proportion of cCREs from the entire registry that belong to each category in the original classification (left-side of either panel) compared upon enhancement with novel TSSs (left panel, right side) and **B)** decoy model TSSs (right panel, right side).

**Figure S20. Non-canonical ORF (ncORFs) in novel CLS. A)** Percentage of translated transcripts (i.e., transcripts containing ncORFs) by class: CLS transcripts, lncRNAs (v27) and protein-coding genes (v27). **B)** Number of translated transcripts per class (as in A), and by the tissue in which translation is detected. Testis means only in testis, testis-brain only in testis and brain, all in the three tissues including liver. Translated sequences were identified with RibORF v2.0, using Ribo-Seq data from Wang et al.[32].

**Figure S21. GWAS frequency in novel CLS. A)** GWAS frequency (hits/100kb) computed along the exon projection of novel CLS transcripts across the different targeted catalogs, colored by focus of targeted element. On top right the frequency computed for *i)* protein-coding genes, *ii)* lncRNAs, and *iii)* decoy models. For visualization purposes, the frequencies for gene body (9.2) and exon (58.72) in CLS captured via the GWAS catalog are not reported. **B)** Distribution of GWAS density computed per transcript in *i)* annotated lncRNA according to GENCODE v27, *ii)* novel CLS models, *iii)* decoy models, and *iv)* protein-coding genes annotated as of GENCODE v27.

**Figure S22. Novel host genes of small RNAs.** Frequency of per-transcript exon and splice junction, and unique-intron splice junction mean PhyloP scores for lncRNAs hosting miR-NAs or snoRNAs outside of protein-coding loci. The dashed red lines indicate the range considered under neutral selection. **A)** 454 GENCODE v27 host lncRNA transcripts with 20% of exons and 64% of the splice junctions classified as conserved, and 71% and 28% respectively neutral-evolving and 307 host unique-introns with 51% conserved, **B)** 4,087 GENCODE v47 host lncRNA transcripts derived from CLS models with 39% of exons and 74% of the splice junctions classified as conserved and 74% and 23% respectively neutral-evolving and 1,104 unique host introns with 48% conserved.

# Supplementary Tables Captions:

**Table S1.** Targeted regions from various catalogs included in the capture panel.

**Table S2.** Targeted regions from various catalogs that were liftedOver and from human to mouse (liftedOverFeatures) and help detect transcription in mouse.

**Table S3.** Summary generated by LyRic reporting samples metadata and sequencing details as well as several statistics at read and transcript level.
https://guigolab.github.io/CLS3_GENCODE/summary_GENCODE.html

**Table S4.** Novelty tags assigned to CLS transcripts based on various GffCompare[28] classes.

**Table S5.** The overlap of TSSs from known and CLS transcripts with repetitive regions within the human genome.

**Table S6.** Counts of current GENCODE genes and transcripts created using CLS data **1)** since human releases v27 and v46 and **2)** mouse releases vM16 and vM35.

**Table S7.** Exonic span of different GENCODE annotations, for human and mouse, reporting the fraction of total genomic area covered.

**Table S8. 1)** Number of upregulated pseudogenes and parent genes in human and mouse, based on whether they are targeted or untargeted. **2)** Number of pseudogene-parent gene pairs in human and mouse, grouped by upregulation status in pre-capture, post-capture, or non-significant categories. **3)** Number of differentially expressed pseudogenes and parent genes in human and mouse, based on different sequencing technologies and capturing approaches. **4)** Pseudogene-parent pairs in human and **5)** mouse.

**Table S9.** The overlap of TSSs from known and CLS transcripts with peaks of transcription factor binding from ChIP-Atlas database (the 500 bp window centered on TSS).

**Table S10.** Number of GWAS hits and their density along the gene body and exons of novel CLS transcript models, decoy models, as well as annotations from GENCODE v27.

**Table S11.** PhyloP score distributions from 241 species Zoonomia Cactus alignment for categories of GENCODE transcripts. The columns show the per-transcript and per-unique intron counts with the frequencies of scores categorized as accelerated, neutral, or conserved. The datasets are the decoy models, and GENCODE v27 or v47 transcripts. The GENCODE transcripts are divided into subsets based on various attributes. These protein-coding mRNAs, existing lncRNAs in v27, and novel, CLS-based lncRNAs in v47. 'PC loci" indicate lncRNAs that

overlap with protein-coding genes, and 'non-PC loci' are outside of protein-coding genes. The "host" category are transcripts or unique introns that overlap miRNAs or snoRNAs on the same strand, while "non-host" have no or opposite strand overlap of these small RNAs.

**Table S12.** Summary table of miRNAs count and their nucleotide span in human across annotated and novel genic regions.

**Table S13.** Summary table of miRNAs count and their nucleotide span in mouse across annotated and novel genic regions.

**Table S14.** Small RNAs in annotated and novel genic regions. The tables list counts of small RNAs and nucleotide enrichments/depletion for the respective regions (genic/intergenic, exonic/intronic) in human.

**Table S15.** Small RNAs in annotated and novel genic regions. The tables list counts of small RNAs and nucleotide enrichments/depletion for the respective regions (genic/intergenic, exonic/intronic) in mouse.

## References:


1. Harrow, J. *et al.* GENCODE: producing a reference annotation for ENCODE. *Genome Biol.* **7**, S4 (2006).
2. Djebali, S. *et al.* Efficient targeted transcript discovery via array-based normalization of RACE libraries. *Nat. Methods* **5**, 629–35 (2008).
3. Howald, C. *et al.* Combining RT-PCR-seq and RNA-seq to catalog all genic elements encoded in the human genome. *Genome Res.* **22**, 1698–710 (2012).
4. Lagarde, J. *et al.* Extension of human lncRNA transcripts by RACE coupled with long-read high-throughput sequencing (RACE-Seq). *Nat. Commun.* **7**, (2016).
5. Lagarde, J. *et al.* High-throughput annotation of full-length long noncoding RNAs with capture long-read sequencing. *Nat. Genet.* **49**, 1731–1740 (2017).
6. Carbonell Sala, S., Uszczyńska-Ratajczak, B., Lagarde, J., Johnson, R. & Guigó, R. Annotation of Full-Length Long Noncoding RNAs with Capture Long-Read Sequencing (CLS). in *Functional Analysis of Long Non-Coding RNAs* (ed. Cao, H.) vol. 2254 133–159 (Springer US, New York, NY, 2021).
7. Derrien, T. *et al.* The GENCODE v7 catalog of human long noncoding RNAs: analysis of their gene structure, evolution, and expression. *Genome Res.* **22**, 1775–89 (2012).
8. Roux, B. T., Heward, J. A., Donnelly, L. E., Jones, S. W. & Lindsay, M. A. Catalog of Differentially Expressed Long Non-Coding RNA following Activation of Human and Mouse Innate Immune Response. *Front. Immunol.* **8**, 1038 (2017).
9. Uszczynska-Ratajczak, B., Lagarde, J., Frankish, A., Guigó, R. & Johnson, R. Towards a complete map of the human long non-coding RNA transcriptome. *Nat. Rev. Genet.* **19**, 535–548 (2018).
10. Iyer, M. K. *et al.* The landscape of long noncoding RNAs in the human transcriptome. *Nat. Genet.* **47**, 199–208 (2015).
11. Hon, C.-C. *et al.* An atlas of human long non-coding RNAs with accurate 5′ ends. *Nature* **543**, 199–204 (2017).
12. You, B.-H., Yoon, S.-H. & Nam, J.-W. High-confidence coding and noncoding transcriptome maps. *Genome Res.* **27**, 1050–1062 (2017).
13. Fang, S. *et al.* NONCODEV5: A comprehensive annotation database for long non-coding RNAs. *Nucleic Acids Res.* **46**, D308–D314 (2018).
14. Visel, A., Minovitsky, S., Dubchak, I. & Pennacchio, L. A. VISTA Enhancer Browser–a database of tissue-specific human enhancers. *Nucleic Acids Res.* **35**, D88-92 (2007).
15. Yao, Z., Weinberg, Z. & Ruzzo, W. L. CMfinder—a covariance model based RNA motif finding algorithm. *Bioinformatics* **22**, 445–452 (2006).
16. Welter, D. *et al.* The NHGRI GWAS Catalog, a curated resource of SNP-trait associations. *Nucleic Acids Res.* **42**, D1001-6 (2014).
17. Sollis, E. *et al.* The NHGRI-EBI GWAS Catalog: knowledgebase and deposition resource. *Nucleic Acids Res.* **51**, D977–D985 (2023).
18. Lin, M. F., Jungreis, I. & Kellis, M. PhyloCSF: a comparative genomics method to distinguish protein coding and non-coding regions. *Bioinforma. Oxf. Engl.* **27**, i275-82 (2011).

19. Dimitrieva, S. & Bucher, P. UCNEbase—a database of ultraconserved non-coding elements and genomic regulatory blocks. *Nucleic Acids Res.* **41**, D101–D109 (2013).
20. Baker, S. C. *et al.* The External RNA Controls Consortium: a progress report. *Nat. Methods* **2**, 731–734 (2005).
21. Carbonell-Sala, S. *et al.* CapTrap-seq: a platform-agnostic and quantitative approach for high-fidelity full-length RNA sequencing. *Nat. Commun.* **15**, 5278 (2024).
22. Lagarde, J. julienlag/LyRic: Zenodo https://doi.org/10.5281/ZENODO.5524444 (2021).
23. Pardo-Palacios, F. J. *et al.* Systematic assessment of long-read RNA-seq methods for transcript identification and quantification. *Nat. Methods* (2024) doi:10.1038/s41592-024-02298-3.
24. Li, H. Minimap2: pairwise alignment for nucleotide sequences. *Bioinforma. Oxf. Engl.* **34**, 3094–3100 (2018).
25. Lagarde, Julien. guigolab/tmerge: Version 1.0. Zenodo https://doi.org/10.5281/ZENODO.11261789 (2024).
26. Lagarde, Julien. The buildLoci GitHub repository: https://github.com/julienlag/buildLoci.
27. Pardo-Palacios, F. J. *et al.* SQANTI3: curation of long-read transcriptomes for accurate identification of known and novel isoforms. *Nat. Methods* (2024) doi:10.1038/s41592-024-02229-2.
28. Pertea, G. & Pertea, M. GFF Utilities: GffRead and GffCompare. *F1000Research* **9**, 304 (2020).
29. Wilks, C. *et al.* recount3: summaries and queries for large-scale RNA-seq expression and splicing. *Genome Biol.* **22**, 323 (2021).
30. Quinlan, A. R. & Hall, I. M. BEDTools: a flexible suite of utilities for comparing genomic features. *Bioinformatics* **26**, 841–842 (2010).
31. Kim, M.-S. *et al.* A draft map of the human proteome. *Nature* **509**, 575–581 (2014).
32. Wang, D. *et al.* A deep proteome and transcriptome abundance atlas of 29 healthy human tissues. *Mol. Syst. Biol.* **15**, e8503 (2019).
33. Carlyle, B. C. *et al.* A multiregional proteomic survey of the postnatal human brain. *Nat. Neurosci.* **20**, 1787–1795 (2017).
34. Schiza, C., Korbakis, D., Jarvi, K., Diamandis, E. P. & Drabovich, A. P. Identification of TEX101-associated Proteins Through Proteomic Measurement of Human Spermatozoa Homozygous for the Missense Variant rs35033974*. *Mol. Cell. Proteomics* **18**, 338–351 (2019).
35. Giansanti, P. *et al.* Mass spectrometry-based draft of the mouse proteome. *Nat. Methods* **19**, 803–811 (2022).
36. Deutsch, E. W. *et al.* The ProteomeXchange consortium in 2017: supporting the cultural change in proteomics public data deposition. *Nucleic Acids Res.* **45**, D1100–D1106 (2017).
37. Eng, J. K., Jahan, T. A. & Hoopmann, M. R. Comet: An open-source MS / MS sequence database search tool. *PROTEOMICS* **13**, 22–24 (2013).
38. The, M., MacCoss, M. J., Noble, W. S. & Käll, L. Fast and Accurate Protein False Discovery Rates on Large-Scale Proteomics Data Sets with Percolator 3.0. *J. Am. Soc. Mass Spectrom.* **27**, 1719–1727 (2016).

39. Cerdán-Vélez, D. & Tress, M. L. The T2T-CHM13 reference assembly uncovers essential WASH1 and GPRIN2 paralogues. *Bioinforma. Adv.* **4**, vbae029 (2024).
40. Cerdán-Vélez, D. & Tress, M. L. Lost in the WASH. The functional human WASH complex 1 gene is on chromosome 20. *BioRxiv Prepr. Serv. Biol.* 2023.06.14.544951 (2023) doi:10.1101/2023.06.14.544951.
41. Liao, Y., Smyth, G. K. & Shi, W. featureCounts: an efficient general purpose program for assigning sequence reads to genomic features. *Bioinformatics* **30**, 923–930 (2014).
42. Zhang, Z. *et al.* PseudoPipe: an automated pseudogene identification pipeline. *Bioinformatics* **22**, 1437–1439 (2006).
43. Love, M. I., Huber, W. & Anders, S. Moderated estimation of fold change and dispersion for RNA-seq data with DESeq2. *Genome Biol.* **15**, 550 (2014).
44. Zhao, L. *et al.* NONCODEV6: an updated database dedicated to long non-coding RNA annotation in both animals and plants. *Nucleic Acids Res.* **49**, D165–D171 (2021).
45. Lopez, F., Granjeaud, S., Ara, T., Ghattas, B. & Gautheret, D. The disparate nature of “intergenic” polyadenylation sites. *RNA* **12**, 1794–1801 (2006).
46. sasti gopal das & Tomasz Mądry. cobRNA/ConnectOR-optimized: ConnectOR-optimized v1.0. Zenodo https://doi.org/10.5281/ZENODO.13942053 (2024).
47. Pulido-Quetglas, Carlos. The ConnectOR GitHub repository.
48. Quinlan, A. R. BEDTools: The Swiss-Army Tool for Genome Feature Analysis. *Curr. Protoc. Bioinforma. Ed. Board Andreas Baxevanis Al* **47**, 11.12.1-11.12.34 (2014).
49. Miga, K. H. *et al.* Centromere reference models for human chromosomes X and Y satellite arrays. *Genome Res.* **24**, 697–707 (2014).
50. Rozowsky, J. *et al.* The EN-TEx resource of multi-tissue personal epigenomes & variant-impact models. *Cell* **186**, 1493-1511.e40 (2023).
51. Zou, Z., Ohta, T. & Oki, S. ChIP-Atlas 3.0: a data-mining suite to explore chromosome architecture together with large-scale regulome data. *Nucleic Acids Res.* **52**, W45–W53 (2024).
52. Ji, Z., Song, R., Regev, A. & Struhl, K. Many lncRNAs, 5’UTRs, and pseudogenes are translated and some are likely to express functional proteins. *eLife* **4**, e08890 (2015).
53. MacArthur, J. *et al.* The new NHGRI-EBI Catalog of published genome-wide association studies (GWAS Catalog). *Nucleic Acids Res.* **45**, D896–D901 (2017).
54. Boix, C. A., James, B. T., Park, Y. P., Meuleman, W. & Kellis, M. Regulatory genomic circuitry of human disease loci by integrative epigenomics. *Nature* **590**, 300–307 (2021).
55. Ramírez, F. *et al.* deepTools2: a next generation web server for deep-sequencing data analysis. *Nucleic Acids Res.* **44**, W160–W165 (2016).
56. Raney, B. J. *et al.* The UCSC Genome Browser database: 2024 update. *Nucleic Acids Res.* **52**, D1082–D1088 (2024).
57. Dobin, A. *et al.* STAR: ultrafast universal RNA-seq aligner. *Bioinformatics* **29**, 15–21 (2013).
58. The FANTOM Consortium *et al.* An atlas of active enhancers across human cell types and tissues. *Nature* **507**, 455–461 (2014).
59. Noguchi, S. *et al.* FANTOM5 CAGE profiles of human and mouse samples. *Sci. Data* **4**, 170112 (2017).

60. Zimmermann, L. *et al.* A Completely Reimplemented MPI Bioinformatics Toolkit with a New HHpred Server at its Core. *J. Mol. Biol.* **430**, 2237–2243 (2018).
61. Abramson, J. *et al.* Accurate structure prediction of biomolecular interactions with AlphaFold 3. *Nature* **630**, 493–500 (2024).

# *Glossary*

‘**Raw reads’** refers to sequencing reads produced on the PacBio or ONT platforms.
‘**Aligned reads**’ refers to sequencing reads that have been preprocessed and aligned to the genome using LyRic.
‘**TAGENE models**’ are *in silico* transcripts filtered in by the TAGENE workflow described below, based on the processing of aligned reads.
‘**GENCODE models**’ are *in silico* transcripts that will go into the public GENCODE geneset as annotations, having been extrapolated from TAGENE models using the workflow described below.
‘**CLS**‘ Capture Long-read Sequencing, library preparation protocol in which probes against targeted regions of the genome are used to capture tailored transcript sequences.
‘**CLS anchored transcripts**’ are transcript models generated upon anchoring the original LyRic output according to the 5’ and 3’ ends support, and then merge models across tissues, technologies and developmental stages.
‘**CLS transcripts**’ or ‘**CLS models**’ are intron chain models generated upon merging the CLS anchored transcripts, across tissues, technologies and developmental stages, disregarding support information at the 5’ and 3’ ends.
**‘CLS loci**’ are CLS transcripts merged together to build regions of continuous transcription on the same strand, thus generating uniquely identifiable loci.
‘**pre-capture**’ refers to the library preparation employing CapTrap protocol, and therefore extends to all the samples and models yielded from those.
‘**post-capture**’ refers to the library preparation employing CapTrap protocol in conjunction with CLS, and therefore extends to all the samples and models yielded from those.
‘**novel lncRNA transcripts**’ transcripts now annotated in GENCODE v47 because of CLS models.
**‘Intergenic CLS transcripts**’ intron chain models that are located in the intergenic space as of the annotation GENCODE v47, therefore not overlapping any other entry in such. The majority of those models have been discarded prior to TAGENE as not fulfilling the minimal recount score requirement.
**‘novel CLS transcripts**’ intron chain models used to extend or create the novel lncRNA transcript in GENCODE v47, complemented with intergenic CLS transcripts.
