# Supplementary material for: GENCODE: massively expanding the lncRNA catalog through capture long-read RNA sequencing
Source: bioRxiv. 2024 Oct 31:2024.10.29.620654. Preprint. [Version 1] doi: 10.1101/2024.10.29.620654 (PMC11565817; doi:10.1101/2024.10.29.620654)
Supplement: Supplement 2 [file media-2.pdf]

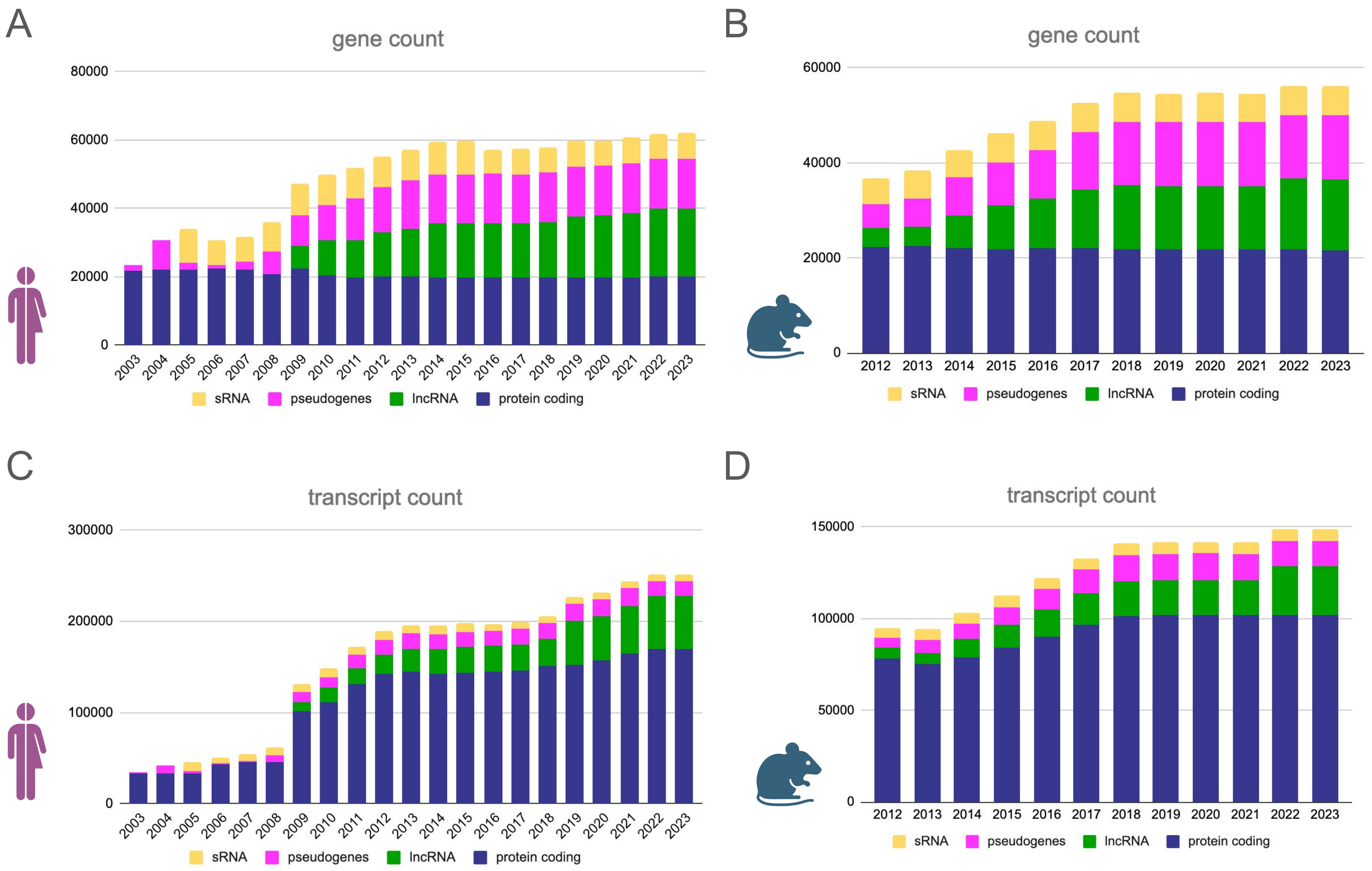
A
gene count
80000
60000
40000
20000
0
2003
2004
2005
2006
2007
2008
2009
2010
2011
2012
2013
2014
2015
2016
2017
2018
2019
2020
2021
2022
2023
sRNA
pseudogenes
lncRNA
protein coding
B
gene count
60000
40000
20000
0
2012
2013
2014
2015
2016
2017
2018
2019
2020
2021
2022
2023
sRNA
pseudogenes
lncRNA
protein coding
C
transcript count
300000
200000
100000
0
2003
2004
2005
2006
2007
2008
2009
2010
2011
2012
2013
2014
2015
2016
2017
2018
2019
2020
2021
2022
2023
sRNA
pseudogenes
lncRNA
protein coding
D
transcript count
150000
100000
50000
0
2012
2013
2014
2015
2016
2017
2018
2019
2020
2021
2022
2023
sRNA
pseudogenes
lncRNA
protein coding

# Fig S1

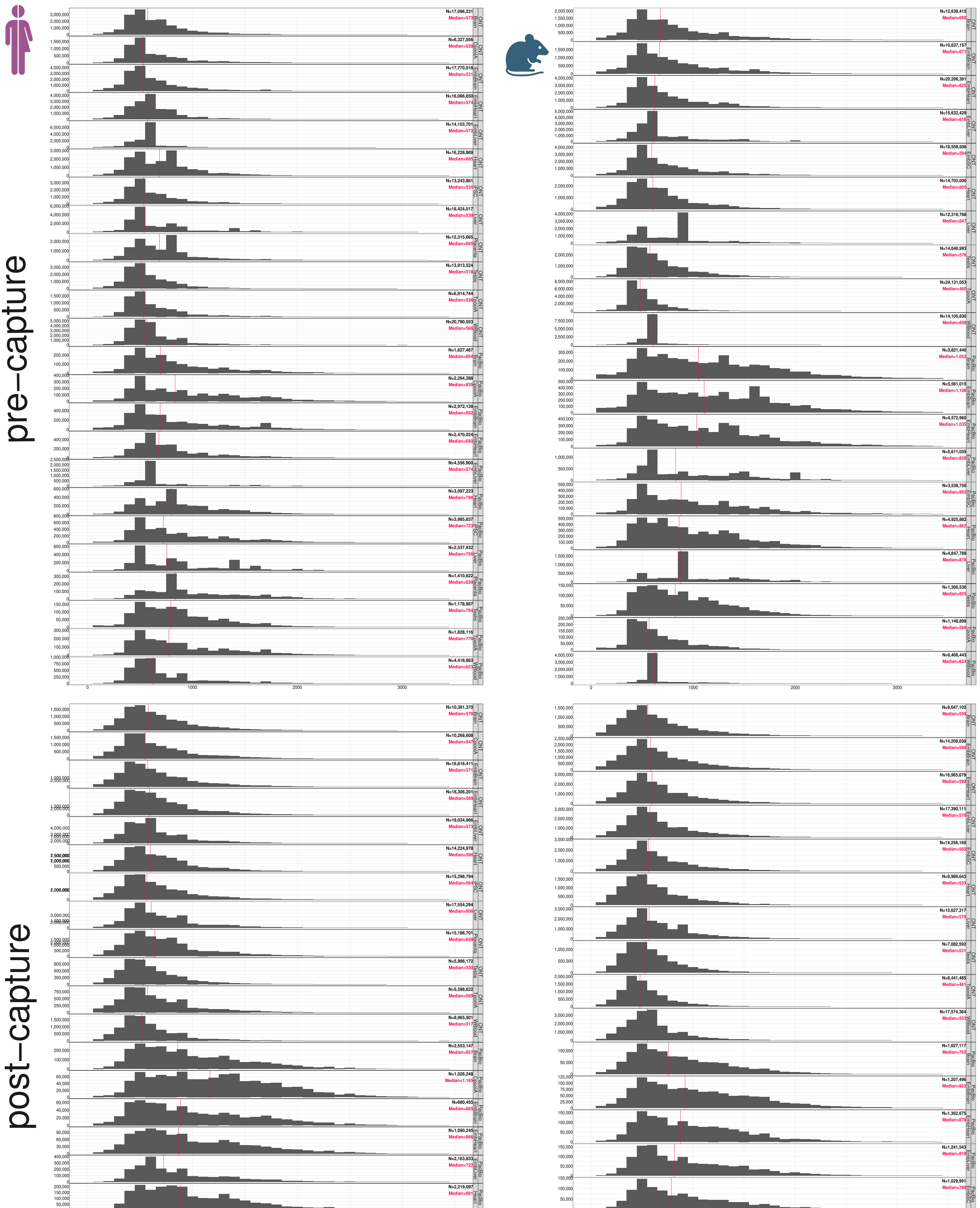


Figure S2

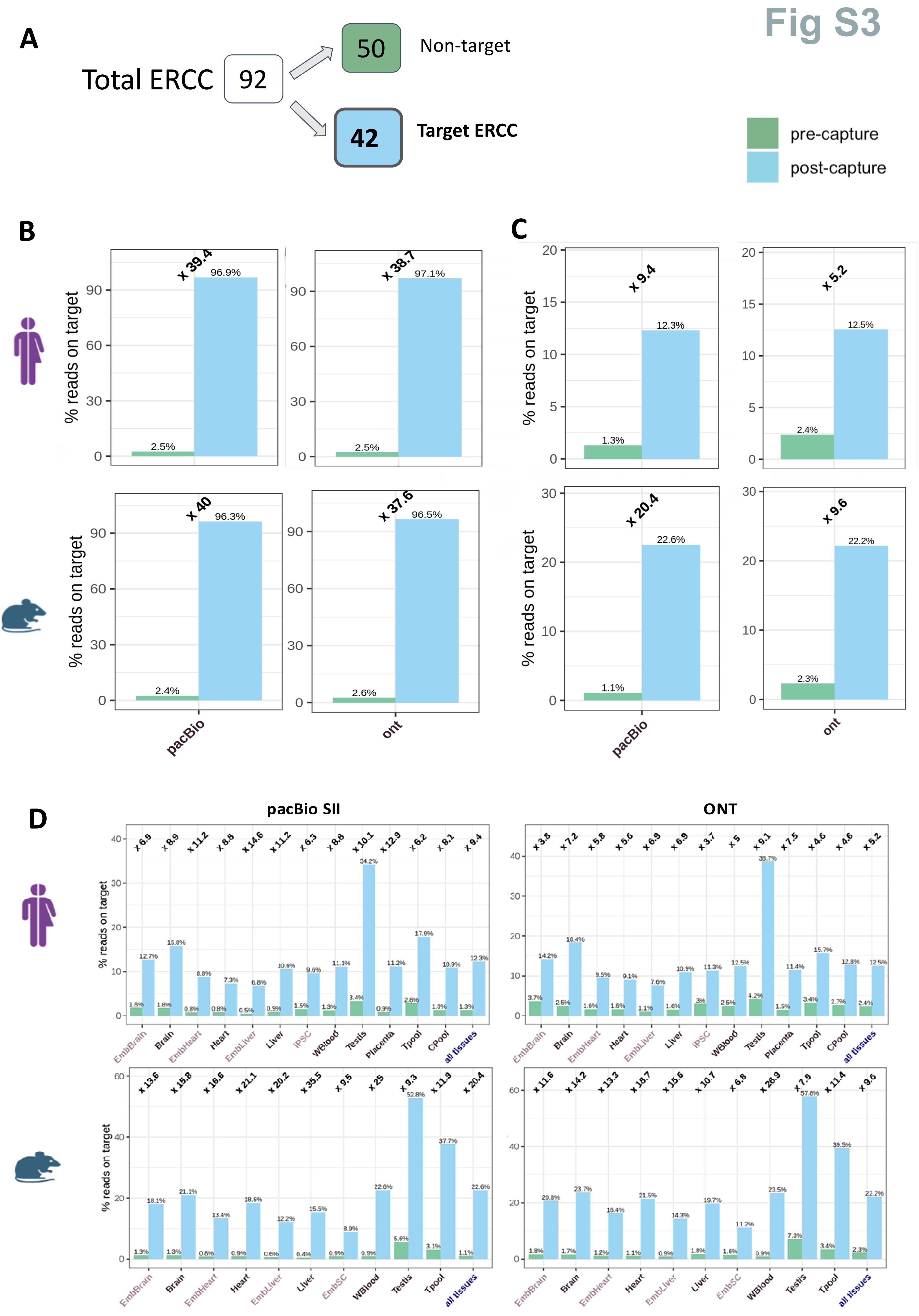
Fig S3
A
Total ERCC
92
50
Non-target
42
Target ERCC
pre-capture
post-capture
B
C
% reads on target
pacBio
ont
D
pacBio SII
ONT
EmbBrain
Brain
EmbHeart
Heart
EmbLiver
Liver
iPSC
WBlood
Testis
Placenta
Tpool
CPool
all tissues
EmbSC

# Fig S4

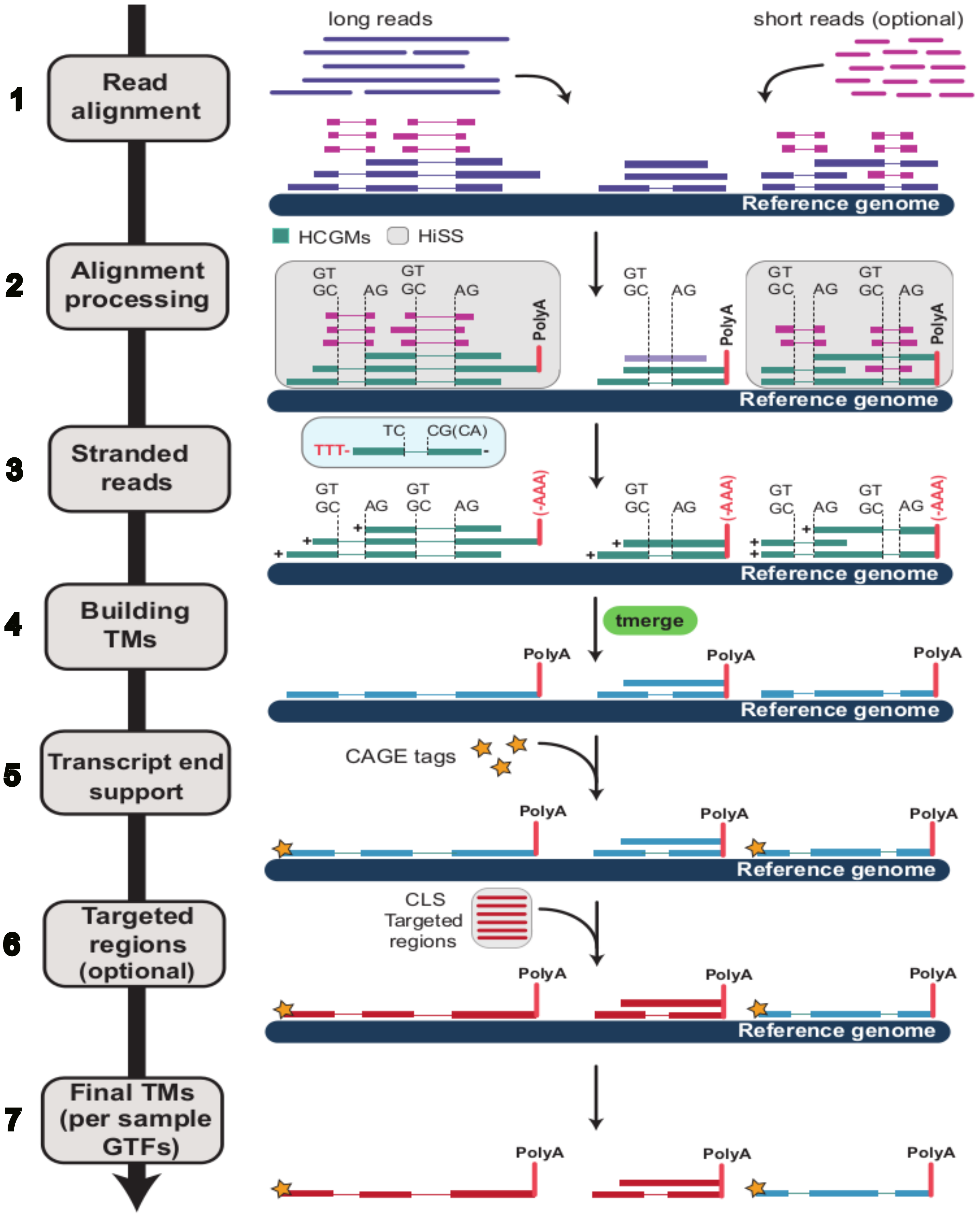

1 Read alignment
2 Alignment processing
3 Stranded reads
4 Building TMs
5 Transcript end support
6 Targeted regions (optional)
7 Final TMs (per sample GTFs)
long reads
short reads (optional)
Reference genome
HCGMs
HiSS
GT
GC
AG
PolyA
TC
CG(CA)
TTT-
(-AAA)
tmerge
CAGE tags
CLS Targeted regions

# Fig S5

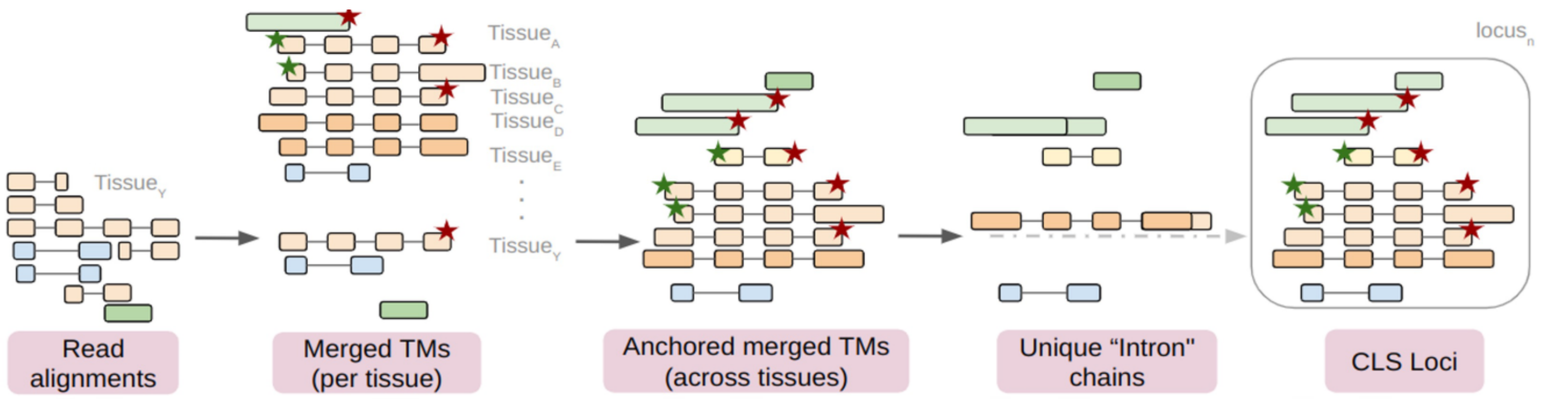

Tissue$_A$
Tissue$_B$
Tissue$_C$
Tissue$_D$
Tissue$_E$
Tissue$_Y$
Tissue$_Y$
locus$_n$
Read alignments
Merged TMs (per tissue)
Anchored merged TMs (across tissues)
Unique “Intron" chains
CLS Loci

# Fig S6

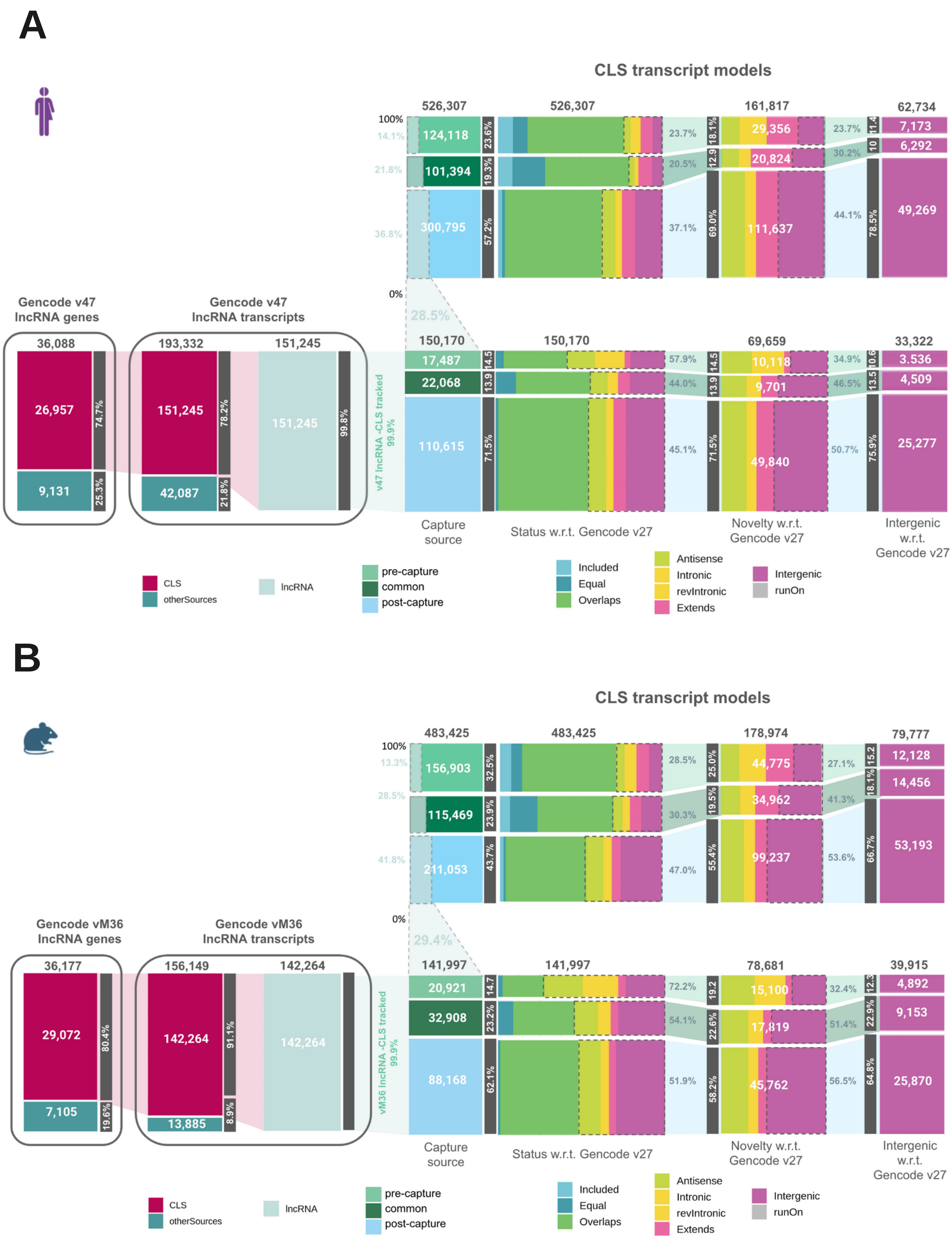

A
CLS transcript models
Gencode v47 lncRNA genes
Gencode v47 lncRNA transcripts
36,088
26,957
9,131
74.7%
25.3%
193,332
151,245
42,087
78.2%
21.8%
151,245
99.8%
v47 lncRNA -CLS tracked 99.9%
526,307
124,118
101,394
300,795
161,817
29,356
20,824
111,637
62,734
7,173
6,292
49,269
150,170
17,487
22,068
110,615
69,659
10,118
9,701
49,840
33,322
3.536
4,509
25,277
28.5%
Capture source
Status w.r.t. Gencode v27
Novelty w.r.t. Gencode v27
Intergenic w.r.t. Gencode v27
CLS
otherSources
lncRNA
pre-capture
common
post-capture
Included
Equal
Overlaps
Antisense
Intronic
revIntronic
Extends
Intergenic
runOn
B
CLS transcript models
Gencode vM36 lncRNA genes
Gencode vM36 lncRNA transcripts
36,177
29,072
7,105
80.4%
19.6%
156,149
142,264
13,885
91.1%
8.9%
142,264
vM36 lncRNA -CLS tracked 99.9%
483,425
156,903
115,469
211,053
178,974
44,775
34,962
99,237
79,777
12,128
14,456
53,193
141,997
20,921
32,908
88,168
78,681
15,100
17,819
45,762
39,915
4,892
9,153
25,870
29.4%
Capture source
Status w.r.t. Gencode v27
Novelty w.r.t. Gencode v27
Intergenic w.r.t. Gencode v27
CLS
otherSources
lncRNA
pre-capture
common
post-capture
Included
Equal
Overlaps
Antisense
Intronic
revIntronic
Extends
Intergenic
runOn

# Fig S7

A

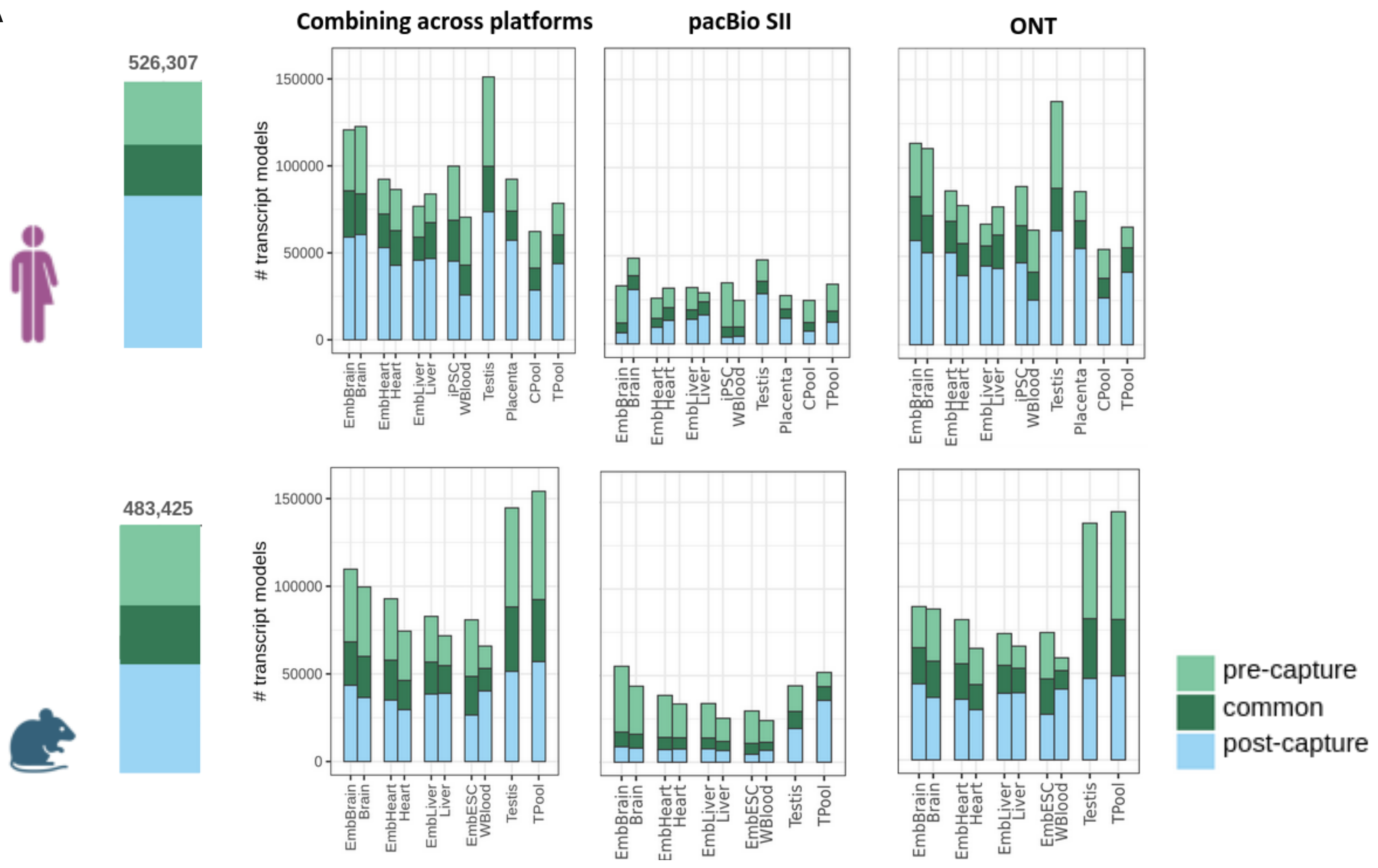

526,307
483,425
Combining across platforms
pacBio SII
ONT
# transcript models
150000
100000
50000
0
EmbBrain
Brain
EmbHeart
Heart
EmbLiver
Liver
iPSC
WBlood
EmbESC
Testis
Placenta
CPool
TPool
pre-capture
common
post-capture


B

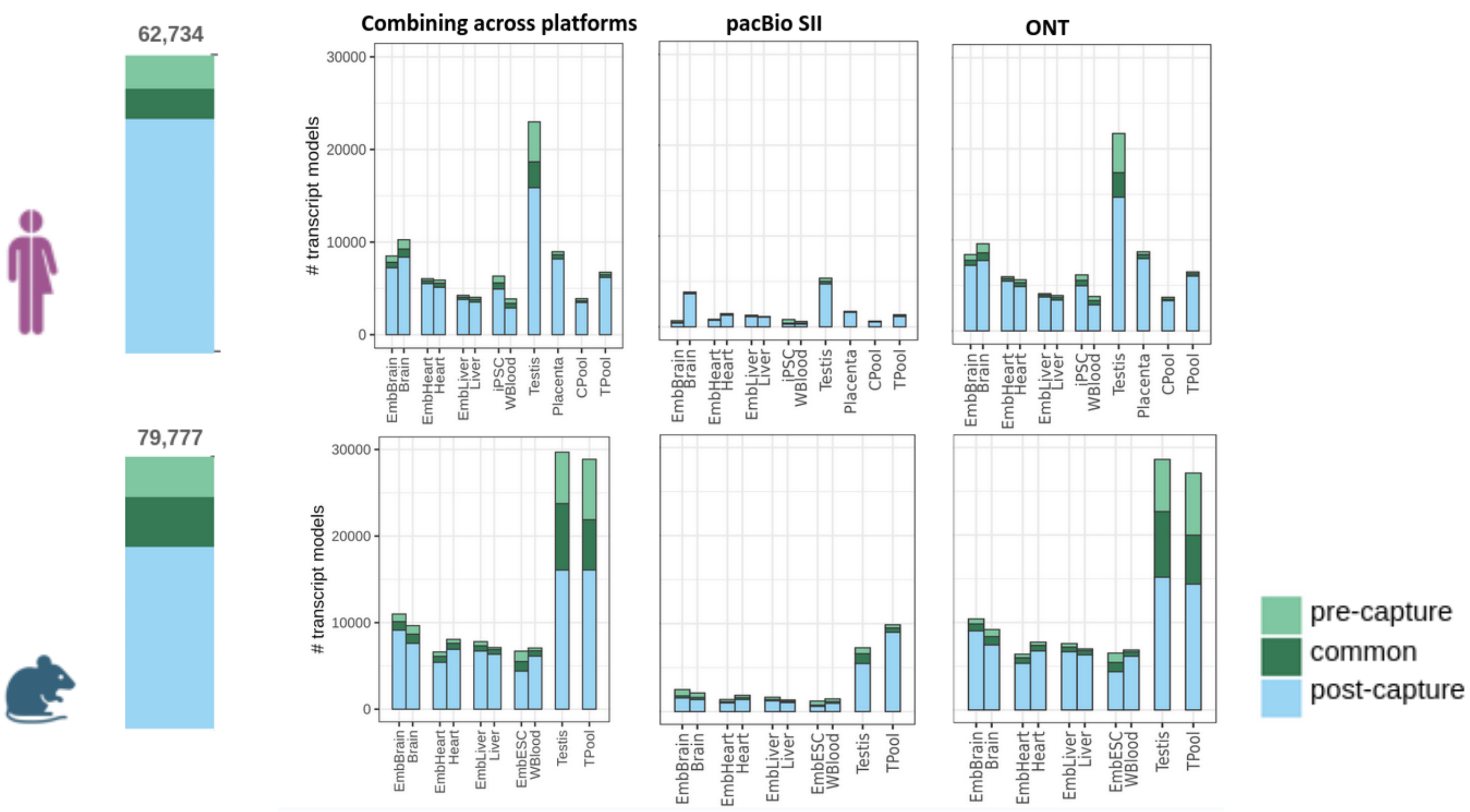

62,734
79,777
Combining across platforms
pacBio SII
ONT
# transcript models
30000
20000
10000
0
EmbBrain
Brain
EmbHeart
Heart
EmbLiver
Liver
iPSC
WBlood
EmbESC
Testis
Placenta
CPool
TPool
pre-capture
common
post-capture

# Fig S8

## A

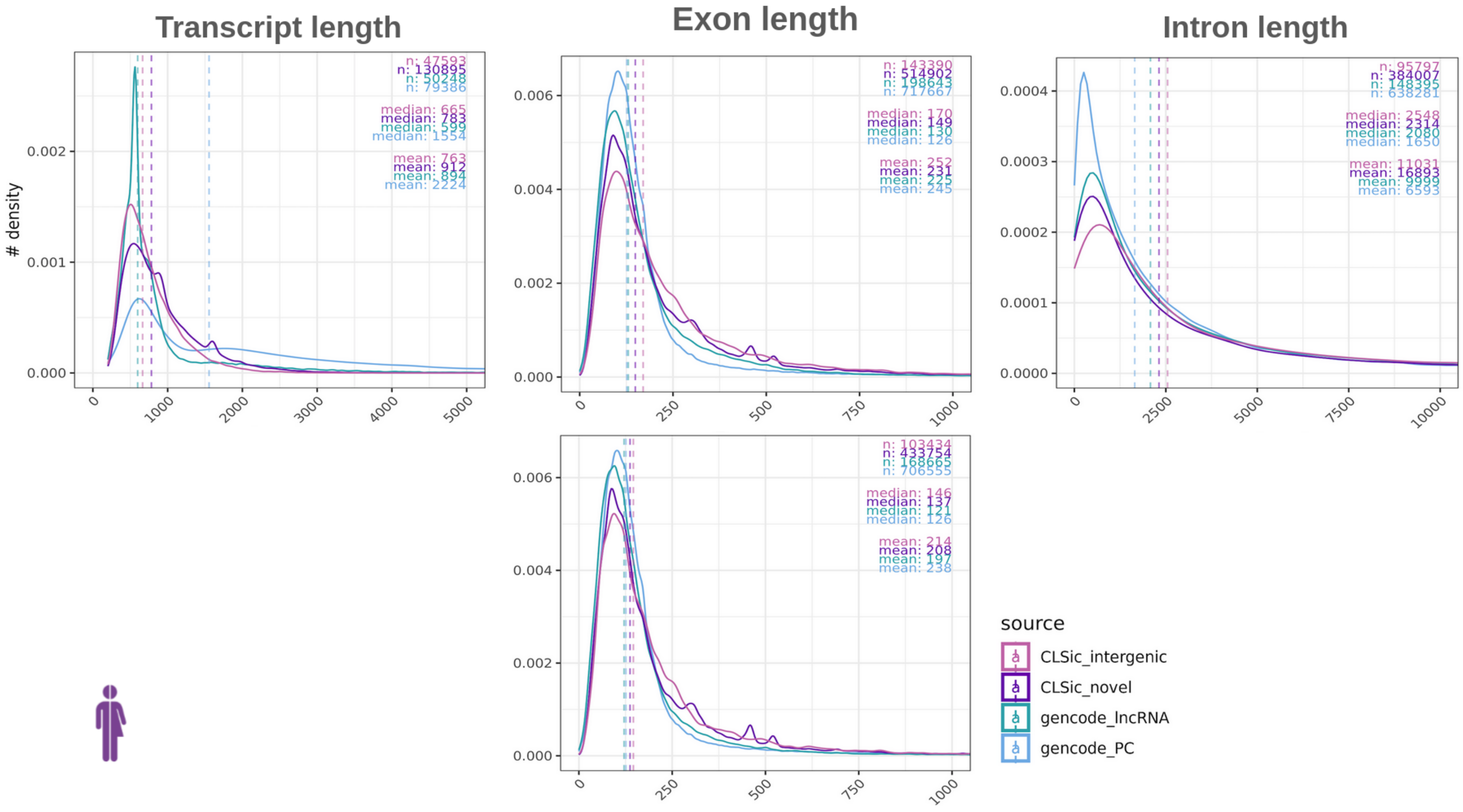

Transcript length
n: 47593
n: 130895
n: 50248
n: 79386
median: 665
median: 783
median: 599
median: 1554
mean: 763
mean: 912
mean: 894
mean: 2224
# density
Exon length
n: 143390
n: 514902
n: 198643
n: 717667
median: 170
median: 149
median: 130
median: 126
mean: 252
mean: 231
mean: 225
mean: 245
Intron length
n: 95797
n: 384007
n: 148395
n: 638281
median: 2548
median: 2314
median: 2080
median: 1650
mean: 11031
mean: 16893
mean: 9999
mean: 6593
n: 103434
n: 433754
n: 168665
n: 706555
median: 146
median: 137
median: 121
median: 126
mean: 214
mean: 208
mean: 197
mean: 238
source
CLSic_intergenic
CLSic_novel
gencode_lncRNA
gencode_PC


## B

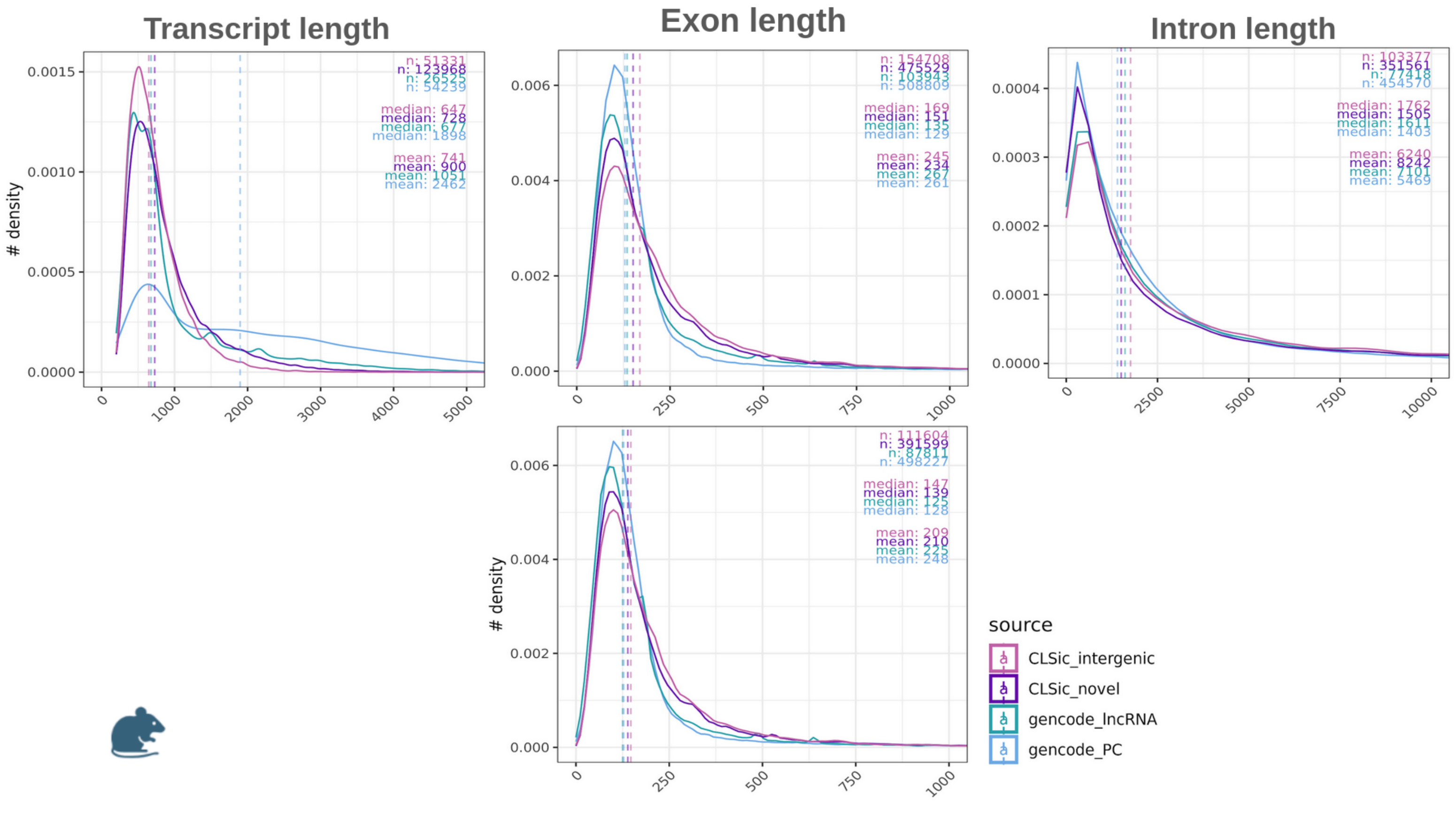

Transcript length
n: 51331
n: 123968
n: 26525
n: 54239
median: 647
median: 728
median: 677
median: 1898
mean: 741
mean: 900
mean: 1051
mean: 2462
# density
Exon length
n: 154708
n: 475529
n: 103943
n: 508809
median: 169
median: 151
median: 135
median: 129
mean: 245
mean: 234
mean: 267
mean: 261
Intron length
n: 103377
n: 351561
n: 77418
n: 454570
median: 1762
median: 1505
median: 1611
median: 1403
mean: 6240
mean: 8242
mean: 7101
mean: 5469
n: 111604
n: 391599
n: 87811
n: 498227
median: 147
median: 139
median: 125
median: 128
mean: 209
mean: 210
mean: 225
mean: 248
# density
source
CLSic_intergenic
CLSic_novel
gencode_lncRNA
gencode_PC

# Fig S9

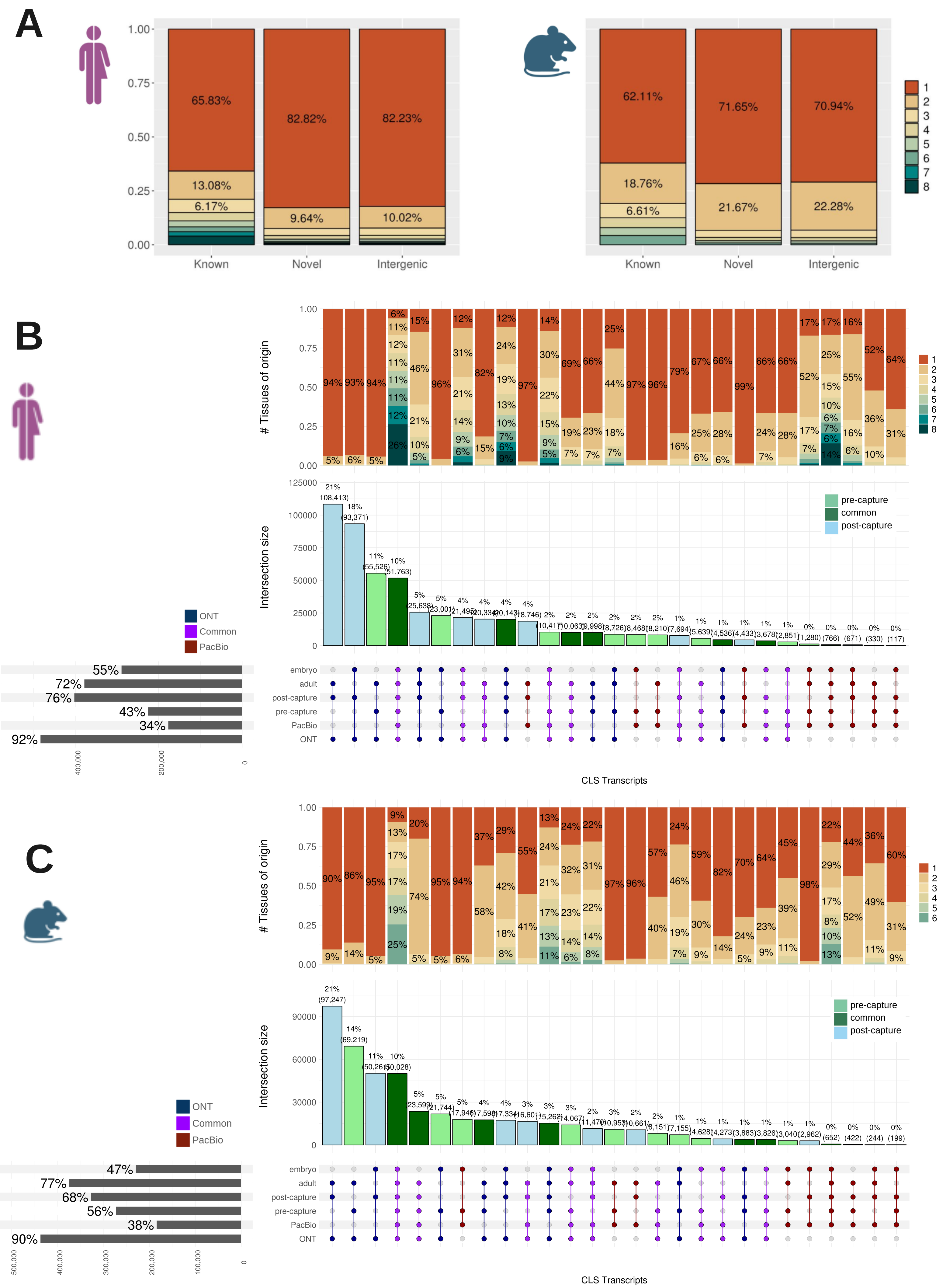
A
1.00
0.75
0.50
0.25
0.00
65.83%
13.08%
6.17%
82.82%
9.64%
82.23%
10.02%
Known
Novel
Intergenic
62.11%
18.76%
6.61%
71.65%
21.67%
70.94%
22.28%
1
2
3
4
5
6
7
8
B
# Tissues of origin
Intersection size
pre-capture
common
post-capture
21% (108,413)
18% (93,371)
11% (55,526)
10% (51,763)
ONT
Common
PacBio
55%
72%
76%
43%
34%
92%
400,000
200,000
0
embryo
adult
post-capture
pre-capture
PacBio
ONT
CLS Transcripts
C
# Tissues of origin
Intersection size
pre-capture
common
post-capture
21% (97,247)
14% (69,219)
11% (50,261)
10% (50,028)
ONT
Common
PacBio
47%
77%
68%
56%
38%
90%
500,000
400,000
300,000
200,000
100,000
0
embryo
adult
post-capture
pre-capture
PacBio
ONT
CLS Transcripts

# Fig S10

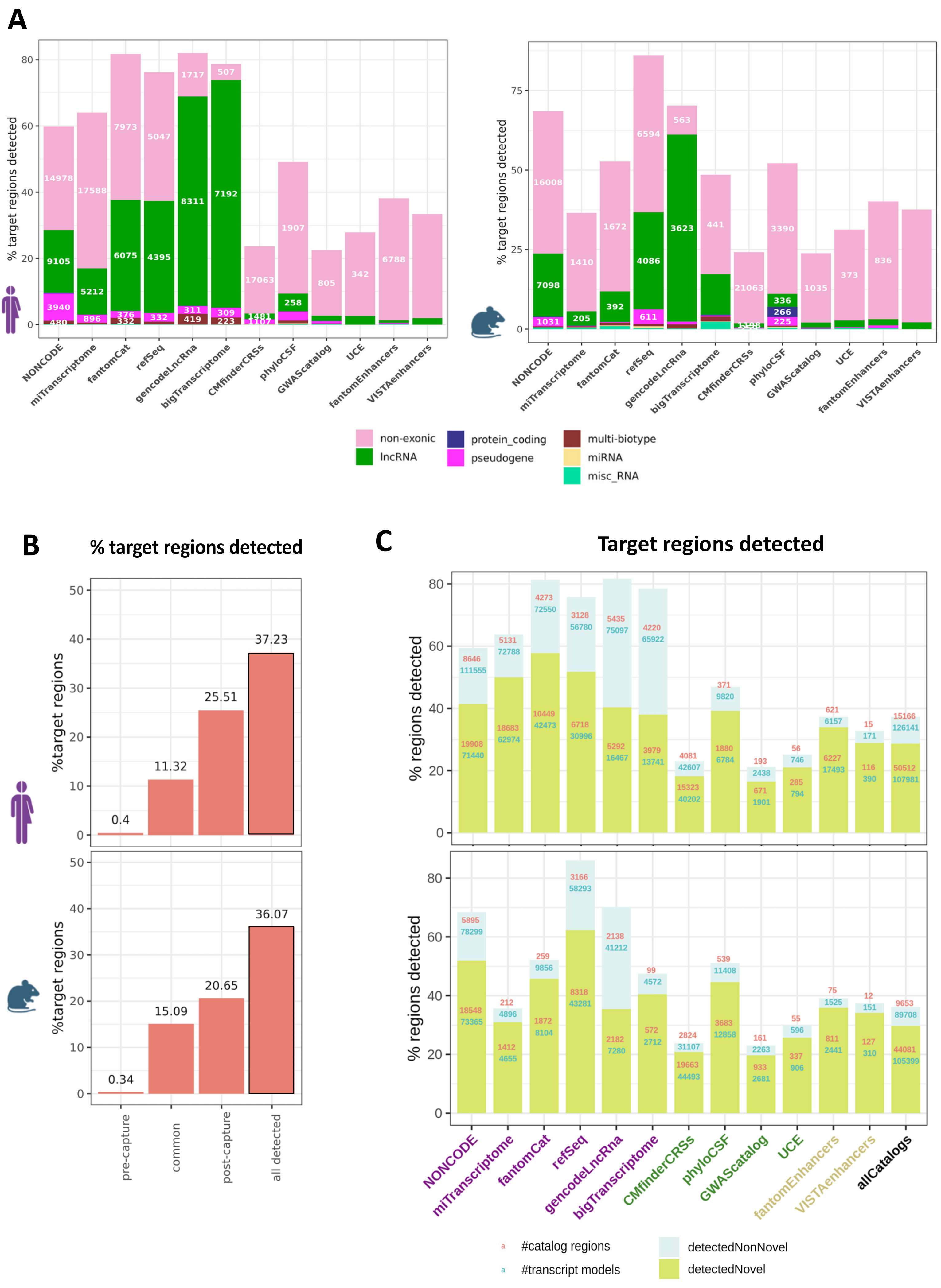
A
% target regions detected
NONCODE
miTranscriptome
fantomCat
refSeq
gencodeLncRna
bigTranscriptome
CMfinderCRSs
phyloCSF
GWAScatalog
UCE
fantomEnhancers
VISTAenhancers
non-exonic
lncRNA
protein_coding
pseudogene
multi-biotype
miRNA
misc_RNA
B
% target regions detected
%target regions
0.4
11.32
25.51
37.23
0.34
15.09
20.65
36.07
pre-capture
common
post-capture
all detected
C
Target regions detected
% regions detected
allCatalogs
#catalog regions
#transcript models
detectedNonNovel
detectedNovel

# Fig S11

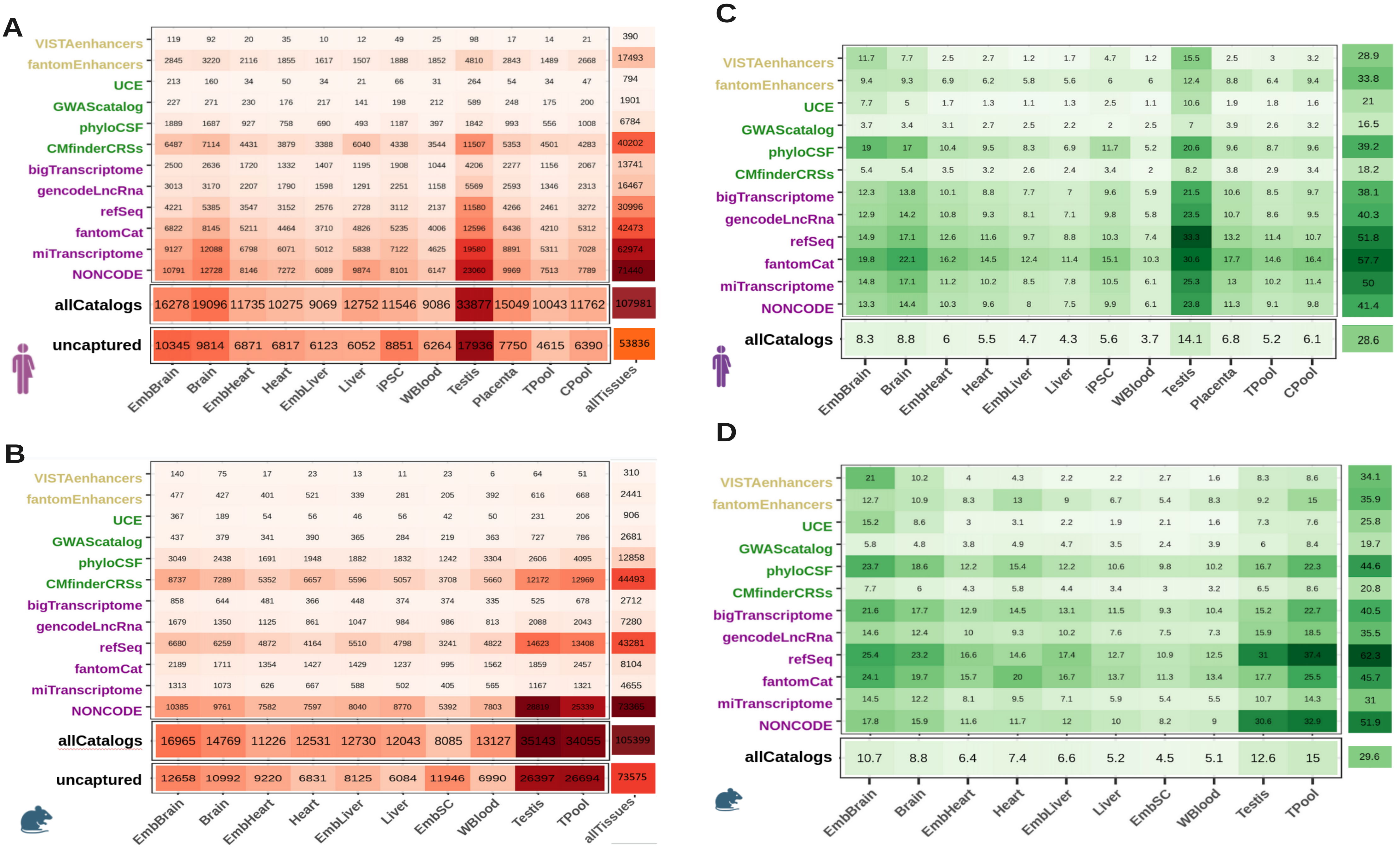
A
VISTAenhancers 119 92 20 35 10 12 49 25 98 17 14 21 390
fantomEnhancers 2845 3220 2116 1855 1617 1507 1888 1852 4810 2843 1489 2668 17493
UCE 213 160 34 50 34 21 66 31 264 54 34 47 794
GWAScatalog 227 271 230 176 217 141 198 212 589 248 175 200 1901
phyloCSF 1889 1687 927 758 690 493 1187 397 1842 993 556 1008 6784
CMfinderCRSs 6487 7114 4431 3879 3388 6040 4338 3544 11507 5353 4501 4283 40202
bigTranscriptome 2500 2636 1720 1332 1407 1195 1908 1044 4206 2277 1156 2067 13741
gencodeLncRna 3013 3170 2207 1790 1598 1291 2251 1158 5569 2593 1346 2313 16467
refSeq 4221 5385 3547 3152 2576 2728 3112 2137 11580 4266 2461 3272 30996
fantomCat 6822 8145 5211 4464 3710 4826 5235 4006 12596 6436 4210 5312 42473
miTranscriptome 9127 12088 6798 6071 5012 5838 7122 4625 19580 8891 5311 7028 62974
NONCODE 10791 12728 8146 7272 6089 9874 8101 6147 23060 9969 7513 7789 71440
allCatalogs 16278 19096 11735 10275 9069 12752 11546 9086 33877 15049 10043 11762 107981
uncaptured 10345 9814 6871 6817 6123 6052 8851 6264 17936 7750 4615 6390 53836
EmbBrain Brain EmbHeart Heart EmbLiver Liver iPSC WBlood Testis Placenta TPool CPool allTissues
B
VISTAenhancers 140 75 17 23 13 11 23 6 64 51 310
fantomEnhancers 477 427 401 521 339 281 205 392 616 668 2441
UCE 367 189 54 56 46 56 42 50 231 206 906
GWAScatalog 437 379 341 390 365 284 219 363 727 786 2681
phyloCSF 3049 2438 1691 1948 1882 1832 1242 3304 2606 4095 12858
CMfinderCRSs 8737 7289 5352 6657 5596 5057 3708 5660 12172 12969 44493
bigTranscriptome 858 644 481 366 448 374 374 335 525 678 2712
gencodeLncRna 1679 1350 1125 861 1047 984 986 813 2088 2043 7280
refSeq 6680 6259 4872 4164 5510 4798 3241 4822 14623 13408 43281
fantomCat 2189 1711 1354 1427 1429 1237 995 1562 1859 2457 8104
miTranscriptome 1313 1073 626 667 588 502 405 565 1167 1321 4655
NONCODE 10385 9761 7582 7597 8040 8770 5392 7803 28819 25339 73365
allCatalogs 16965 14769 11226 12531 12730 12043 8085 13127 35143 34055 105399
uncaptured 12658 10992 9220 6831 8125 6084 11946 6990 26397 26694 73575
EmbBrain Brain EmbHeart Heart EmbLiver Liver EmbSC WBlood Testis TPool allTissues
C
VISTAenhancers 11.7 7.7 2.5 2.7 1.2 1.7 4.7 1.2 15.5 2.5 3 3.2 28.9
fantomEnhancers 9.4 9.3 6.9 6.2 5.8 5.6 6 6 12.4 8.8 6.4 9.4 33.8
UCE 7.7 5 1.7 1.3 1.1 1.3 2.5 1.1 10.6 1.9 1.8 1.6 21
GWAScatalog 3.7 3.4 3.1 2.7 2.5 2.2 2 2.5 7 3.9 2.6 3.2 16.5
phyloCSF 19 17 10.4 9.5 8.3 6.9 11.7 5.2 20.6 9.6 8.7 9.6 39.2
CMfinderCRSs 5.4 5.4 3.5 3.2 2.6 2.4 3.4 2 8.2 3.8 2.9 3.4 18.2
bigTranscriptome 12.3 13.8 10.1 8.8 7.7 7 9.6 5.9 21.5 10.6 8.5 9.7 38.1
gencodeLncRna 12.9 14.2 10.8 9.3 8.1 7.1 9.8 5.8 23.5 10.7 8.6 9.5 40.3
refSeq 14.9 17.1 12.6 11.6 9.7 8.8 10.3 7.4 33.3 13.2 11.4 10.7 51.8
fantomCat 19.8 22.1 16.2 14.5 12.4 11.4 15.1 10.3 30.6 17.7 14.6 16.4 57.7
miTranscriptome 14.8 17.1 11.2 10.2 8.5 7.8 10.5 6.1 25.3 13 10.2 11.4 50
NONCODE 13.3 14.4 10.3 9.6 8 7.5 9.9 6.1 23.8 11.3 9.1 9.8 41.4
allCatalogs 8.3 8.8 6 5.5 4.7 4.3 5.6 3.7 14.1 6.8 5.2 6.1 28.6
EmbBrain Brain EmbHeart Heart EmbLiver Liver iPSC WBlood Testis Placenta TPool CPool
D
VISTAenhancers 21 10.2 4 4.3 2.2 2.2 2.7 1.6 8.3 8.6 34.1
fantomEnhancers 12.7 10.9 8.3 13 9 6.7 5.4 8.3 9.2 15 35.9
UCE 15.2 8.6 3 3.1 2.2 1.9 2.1 1.6 7.3 7.6 25.8
GWAScatalog 5.8 4.8 3.8 4.9 4.7 3.5 2.4 3.9 6 8.4 19.7
phyloCSF 23.7 18.6 12.2 15.4 12.2 10.6 9.8 10.2 16.7 22.3 44.6
CMfinderCRSs 7.7 6 4.3 5.8 4.4 3.4 3 3.2 6.5 8.6 20.8
bigTranscriptome 21.6 17.7 12.9 14.5 13.1 11.5 9.3 10.4 15.2 22.7 40.5
gencodeLncRna 14.6 12.4 10 9.3 10.2 7.6 7.5 7.3 15.9 18.5 35.5
refSeq 25.4 23.2 16.6 14.6 17.4 12.7 10.9 12.5 31 37.4 62.3
fantomCat 24.1 19.7 15.7 20 16.7 13.7 11.3 13.4 17.7 25.5 45.7
miTranscriptome 14.5 12.2 8.1 9.5 7.1 5.9 5.4 5.5 10.7 14.3 31
NONCODE 17.8 15.9 11.6 11.7 12 10 8.2 9 30.6 32.9 51.9
allCatalogs 10.7 8.8 6.4 7.4 6.6 5.2 4.5 5.1 12.6 15 29.6
EmbBrain Brain EmbHeart Heart EmbLiver Liver EmbSC WBlood Testis TPool

# Fig S12

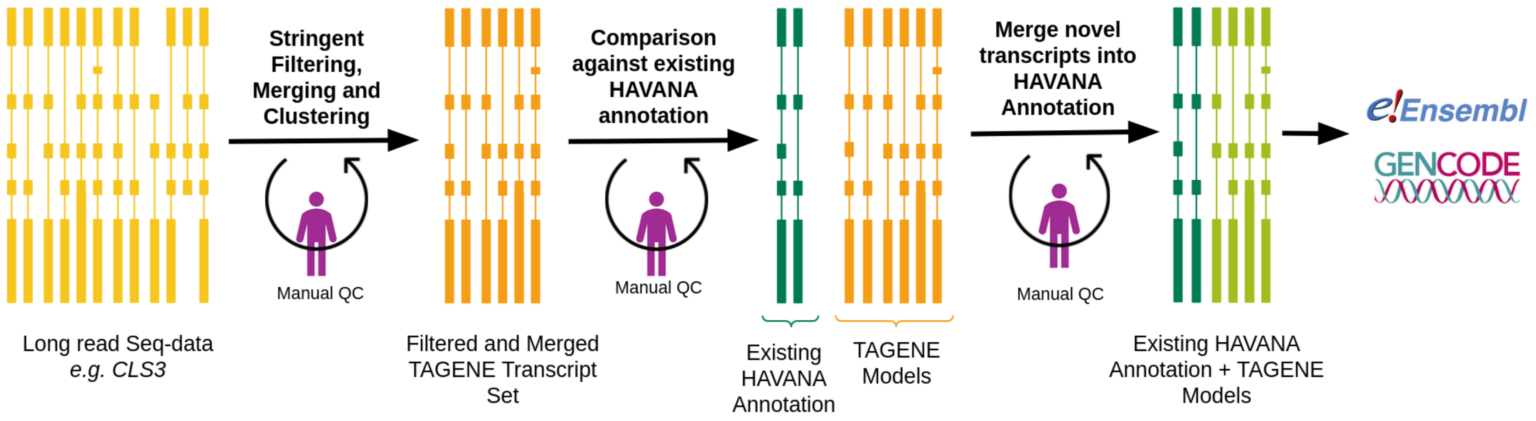
Stringent Filtering, Merging and Clustering
Manual QC
Comparison against existing HAVANA annotation
Manual QC
Merge novel transcripts into HAVANA Annotation
Manual QC
e!Ensembl
GENCODE
Long read Seq-data
*e.g. CLS3*
Filtered and Merged TAGENE Transcript Set
Existing HAVANA Annotation
TAGENE Models
Existing HAVANA Annotation + TAGENE Models

# Fig S13

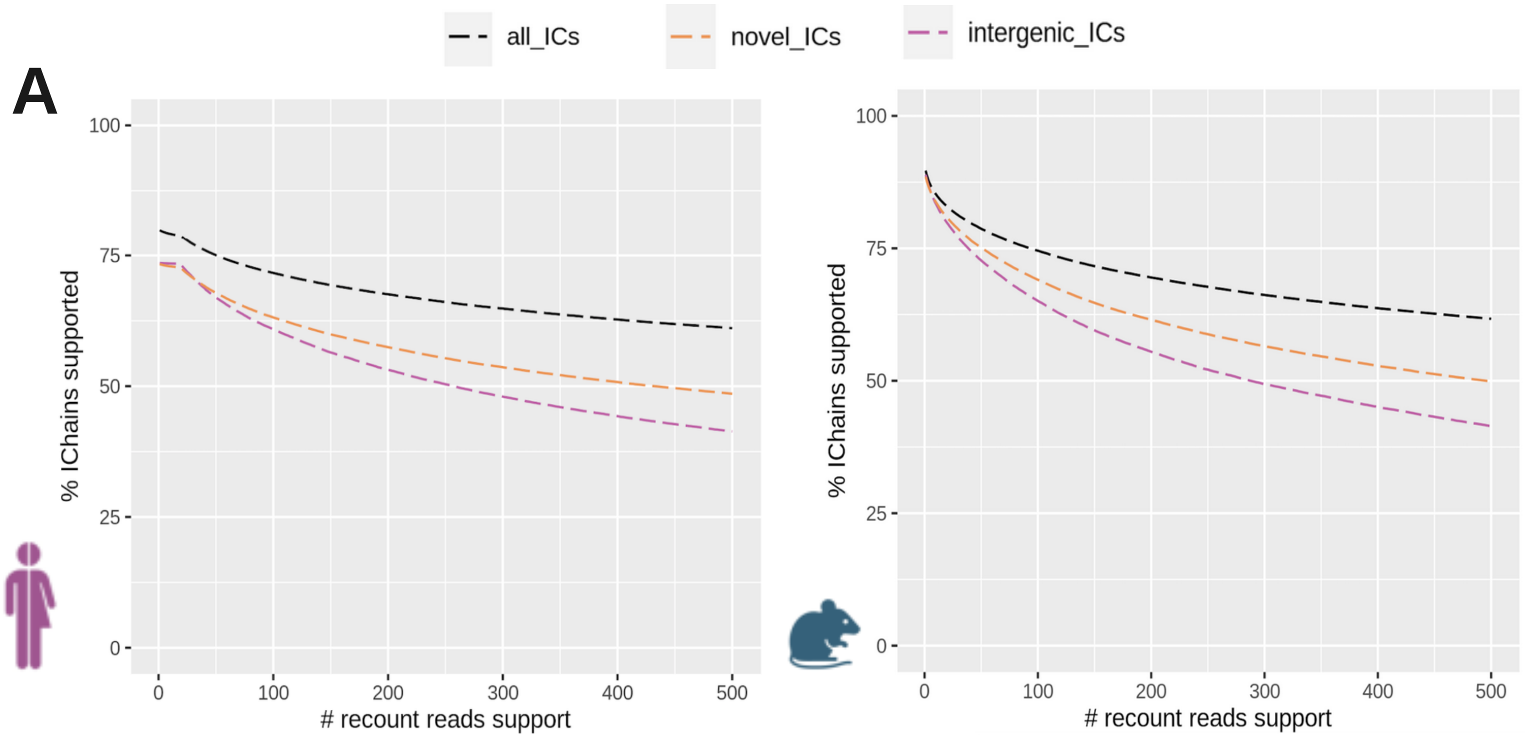

all_ICs
novel_ICs
intergenic_ICs
A
% IChains supported
100
75
50
25
0
0
100
200
300
400
500
# recount reads support
% IChains supported
100
75
50
25
0
0
100
200
300
400
500
# recount reads support

# Fig S14

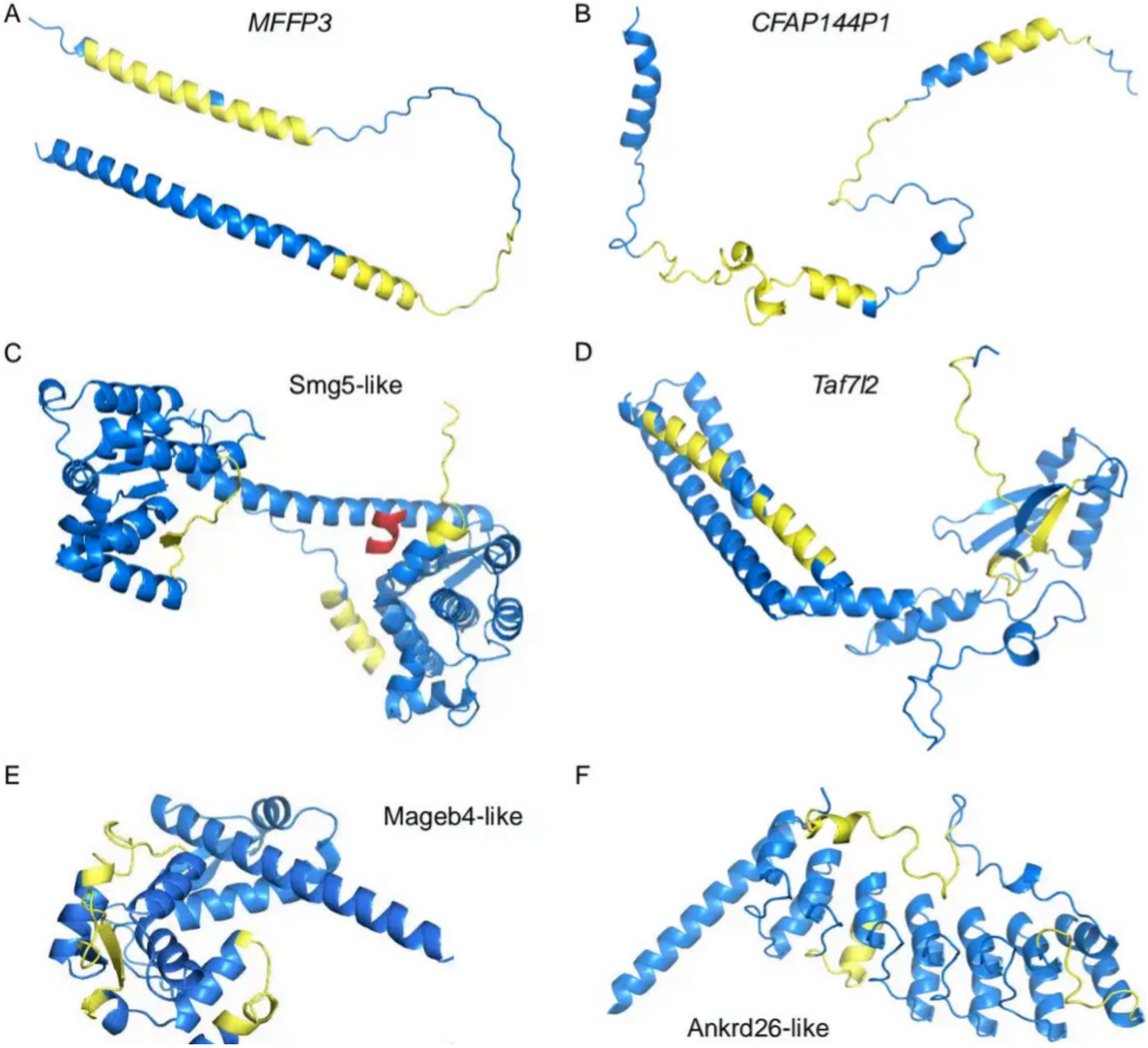

A
MFFP3
B
CFAP144P1
C
Smg5-like
D
Taf7l2
E
Mageb4-like
F
Ankrd26-like

# Fig S15

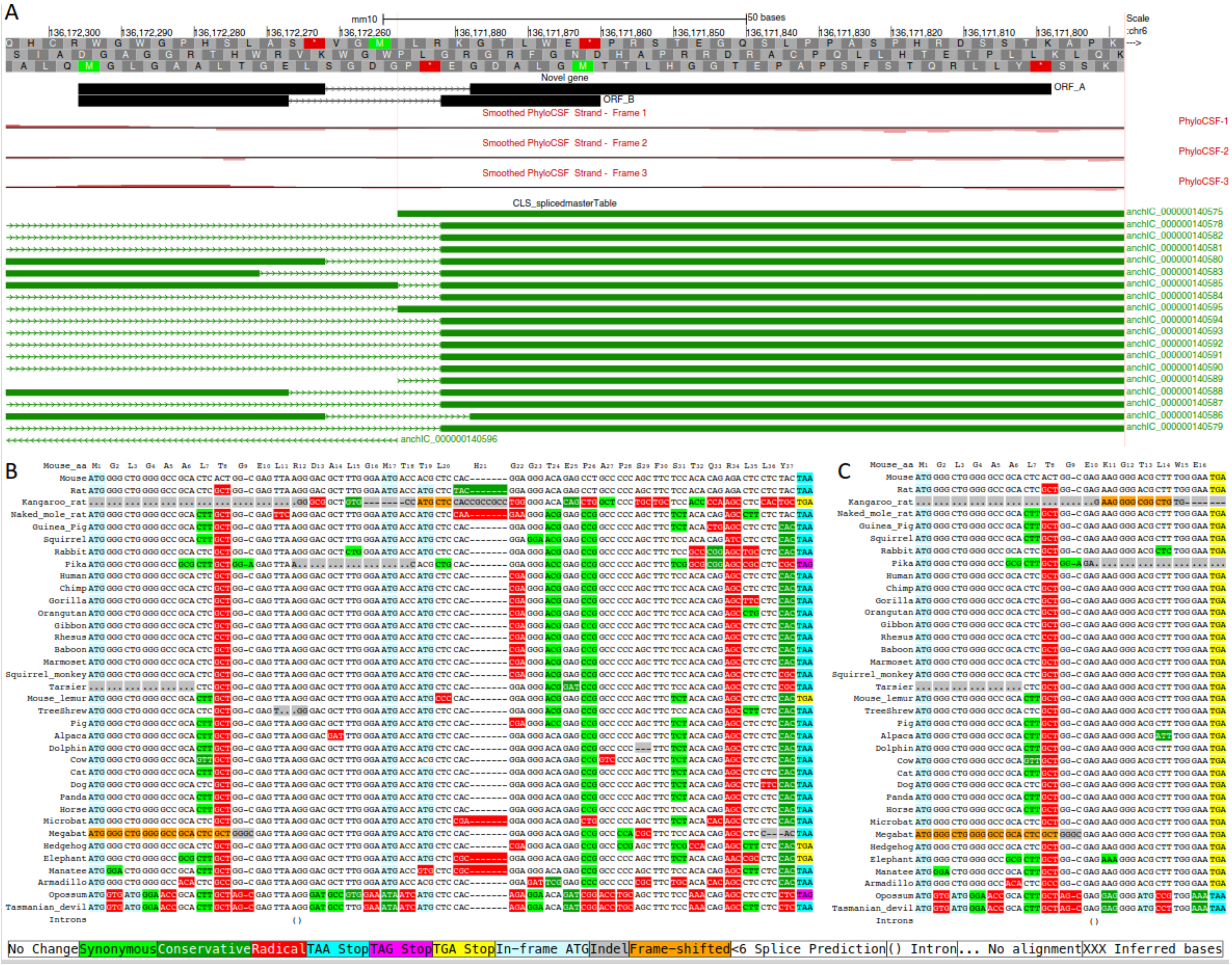
A
B
C
mm10
50 bases
Scale
chr6
Novel gene
ORF_A
ORF_B
Smoothed PhyloCSF Strand - Frame 1
Smoothed PhyloCSF Strand - Frame 2
Smoothed PhyloCSF Strand - Frame 3
PhyloCSF-1
PhyloCSF-2
PhyloCSF-3
CLS_splicedmasterTable
No Change
Synonymous
Conservative
Radical
TAA Stop
TAG Stop
TGA Stop
In-frame ATG
Indel
Frame-shifted
<6 Splice Prediction
() Intron
... No alignment
XXX Inferred bases

# Fig S16

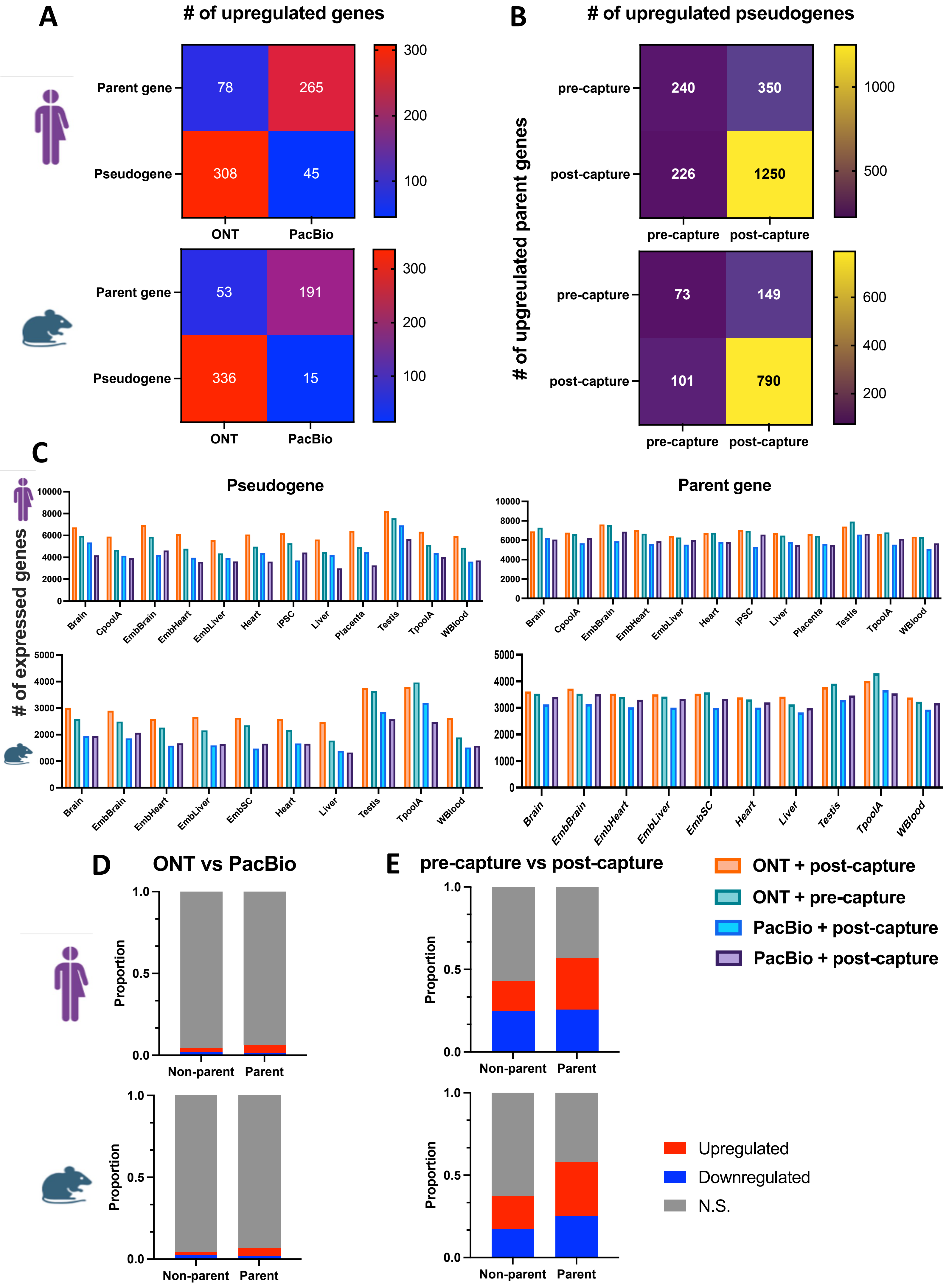

A
# of upregulated genes
Parent gene
Pseudogene
ONT
PacBio
78
265
308
45
53
191
336
15
300
200
100
B
# of upregulated pseudogenes
# of upgreulated parent genes
pre-capture
post-capture
240
350
226
1250
73
149
101
790
1000
500
600
400
200
C
Pseudogene
Parent gene
# of expressed genes
Brain
CpoolA
EmbBrain
EmbHeart
EmbLiver
Heart
iPSC
Liver
Placenta
Testis
TpoolA
WBlood
EmbSC
D
ONT vs PacBio
E
pre-capture vs post-capture
Proportion
Non-parent
Parent
ONT + post-capture
ONT + pre-capture
PacBio + post-capture
PacBio + post-capture
Upregulated
Downregulated
N.S.

Fig S17

A

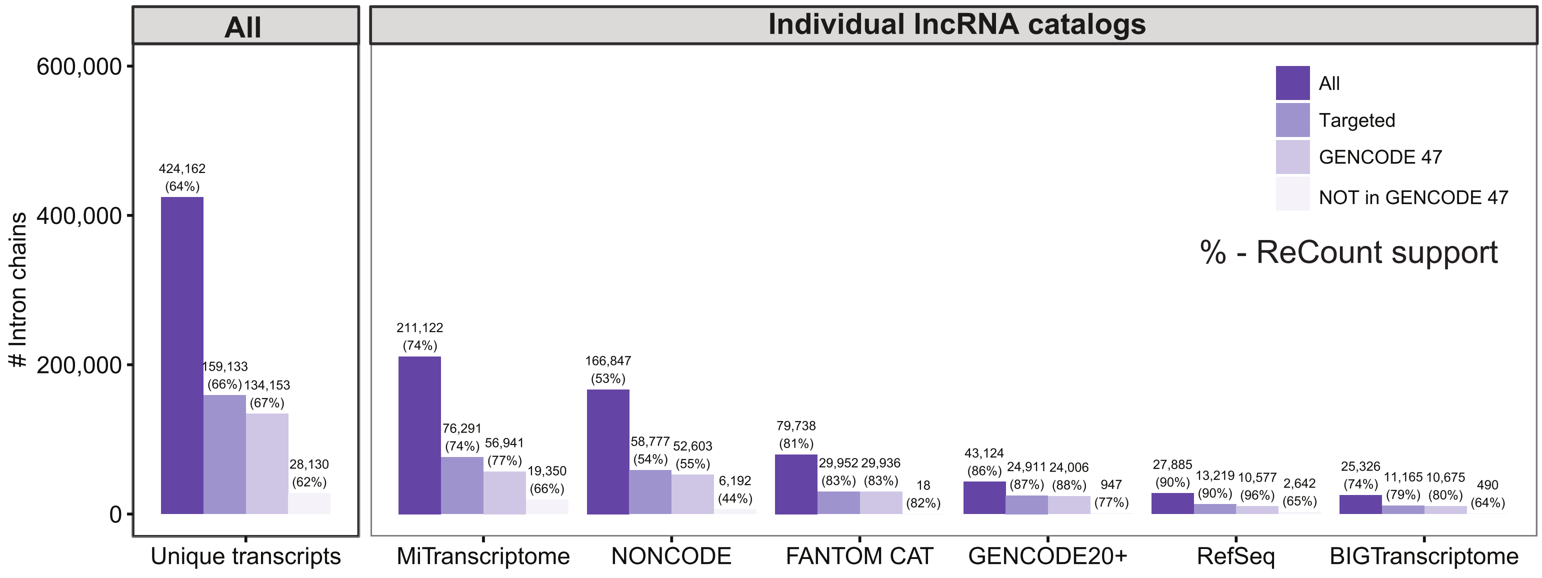
All
Individual lncRNA catalogs
# Intron chains
600,000
400,000
200,000
0
424,162 (64%)
159,133 (66%)
134,153 (67%)
28,130 (62%)
211,122 (74%)
76,291 (74%)
56,941 (77%)
19,350 (66%)
166,847 (53%)
58,777 (54%)
52,603 (55%)
6,192 (44%)
79,738 (81%)
29,952 (83%)
29,936 (83%)
18 (82%)
43,124 (86%)
24,911 (87%)
24,006 (88%)
947 (77%)
27,885 (90%)
13,219 (90%)
10,577 (96%)
2,642 (65%)
25,326 (74%)
11,165 (79%)
10,675 (80%)
490 (64%)
All
Targeted
GENCODE 47
NOT in GENCODE 47
% - ReCount support
Unique transcripts
MiTranscriptome
NONCODE
FANTOM CAT
GENCODE20+
RefSeq
BIGTranscriptome

B

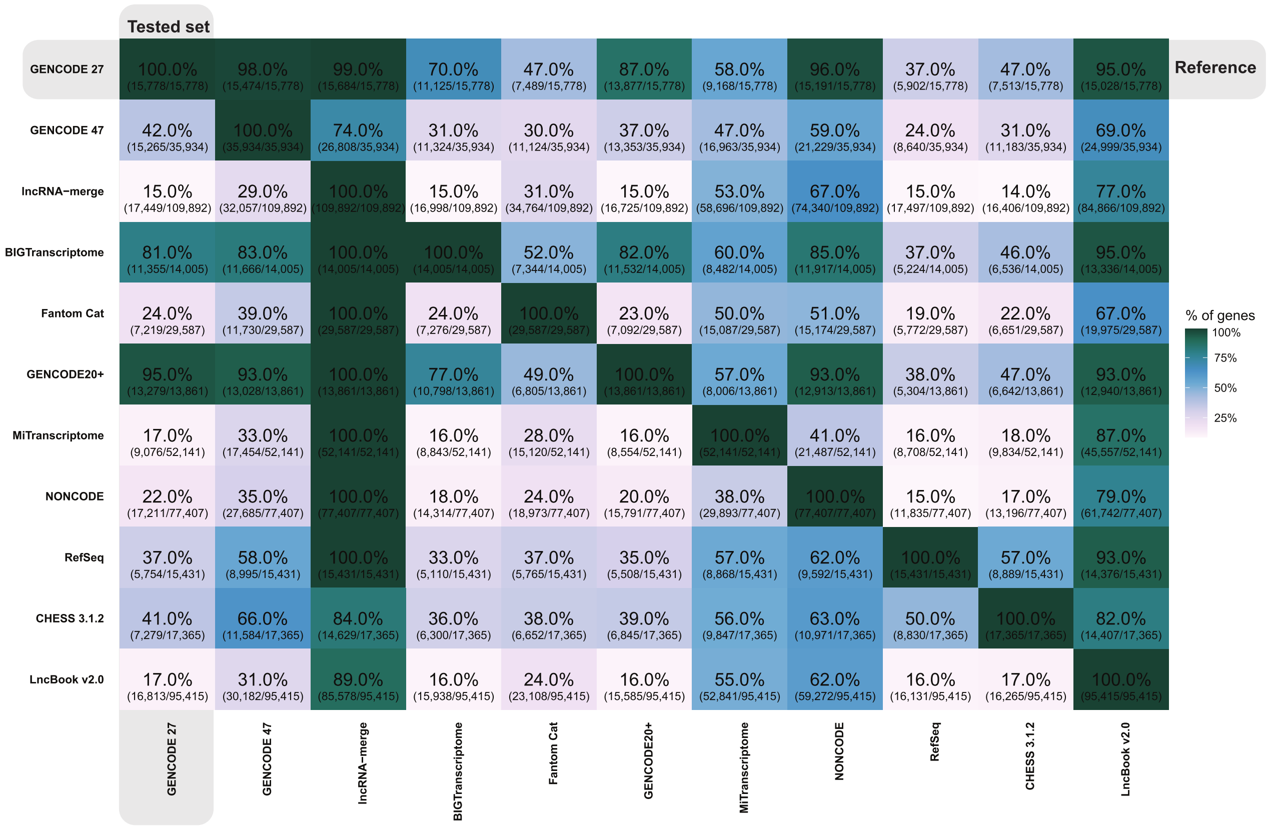
Tested set
Reference
GENCODE 27: 100.0% (15,778/15,778); 98.0% (15,474/15,778); 99.0% (15,684/15,778); 70.0% (11,125/15,778); 47.0% (7,489/15,778); 87.0% (13,877/15,778); 58.0% (9,168/15,778); 96.0% (15,191/15,778); 37.0% (5,902/15,778); 47.0% (7,513/15,778); 95.0% (15,028/15,778)
GENCODE 47: 42.0% (15,265/35,934); 100.0% (35,934/35,934); 74.0% (26,808/35,934); 31.0% (11,324/35,934); 30.0% (11,124/35,934); 37.0% (13,353/35,934); 47.0% (16,963/35,934); 59.0% (21,229/35,934); 24.0% (8,640/35,934); 31.0% (11,183/35,934); 69.0% (24,999/35,934)
lncRNA−merge: 15.0% (17,449/109,892); 29.0% (32,057/109,892); 100.0% (109,892/109,892); 15.0% (16,998/109,892); 31.0% (34,764/109,892); 15.0% (16,725/109,892); 53.0% (58,696/109,892); 67.0% (74,340/109,892); 15.0% (17,497/109,892); 14.0% (16,406/109,892); 77.0% (84,866/109,892)
BIGTranscriptome: 81.0% (11,355/14,005); 83.0% (11,666/14,005); 100.0% (14,005/14,005); 100.0% (14,005/14,005); 52.0% (7,344/14,005); 82.0% (11,532/14,005); 60.0% (8,482/14,005); 85.0% (11,917/14,005); 37.0% (5,224/14,005); 46.0% (6,536/14,005); 95.0% (13,336/14,005)
Fantom Cat: 24.0% (7,219/29,587); 39.0% (11,730/29,587); 100.0% (29,587/29,587); 24.0% (7,276/29,587); 100.0% (29,587/29,587); 23.0% (7,092/29,587); 50.0% (15,087/29,587); 51.0% (15,174/29,587); 19.0% (5,772/29,587); 22.0% (6,651/29,587); 67.0% (19,975/29,587)
GENCODE20+: 95.0% (13,279/13,861); 93.0% (13,028/13,861); 100.0% (13,861/13,861); 77.0% (10,798/13,861); 49.0% (6,805/13,861); 100.0% (13,861/13,861); 57.0% (8,006/13,861); 93.0% (12,913/13,861); 38.0% (5,304/13,861); 47.0% (6,642/13,861); 93.0% (12,940/13,861)
MiTranscriptome: 17.0% (9,076/52,141); 33.0% (17,454/52,141); 100.0% (52,141/52,141); 16.0% (8,843/52,141); 28.0% (15,120/52,141); 16.0% (8,554/52,141); 100.0% (52,141/52,141); 41.0% (21,487/52,141); 16.0% (8,708/52,141); 18.0% (9,834/52,141); 87.0% (45,557/52,141)
NONCODE: 22.0% (17,211/77,407); 35.0% (27,685/77,407); 100.0% (77,407/77,407); 18.0% (14,314/77,407); 24.0% (18,973/77,407); 20.0% (15,791/77,407); 38.0% (29,893/77,407); 100.0% (77,407/77,407); 15.0% (11,835/77,407); 17.0% (13,196/77,407); 79.0% (61,742/77,407)
RefSeq: 37.0% (5,754/15,431); 58.0% (8,995/15,431); 100.0% (15,431/15,431); 33.0% (5,110/15,431); 37.0% (5,765/15,431); 35.0% (5,508/15,431); 57.0% (8,868/15,431); 62.0% (9,592/15,431); 100.0% (15,431/15,431); 57.0% (8,889/15,431); 93.0% (14,376/15,431)
CHESS 3.1.2: 41.0% (7,279/17,365); 66.0% (11,584/17,365); 84.0% (14,629/17,365); 36.0% (6,300/17,365); 38.0% (6,652/17,365); 39.0% (6,845/17,365); 56.0% (9,847/17,365); 63.0% (10,971/17,365); 50.0% (8,830/17,365); 100.0% (17,365/17,365); 82.0% (14,407/17,365)
LncBook v2.0: 17.0% (16,813/95,415); 31.0% (30,182/95,415); 89.0% (85,578/95,415); 16.0% (15,938/95,415); 24.0% (23,108/95,415); 16.0% (15,585/95,415); 55.0% (52,841/95,415); 62.0% (59,272/95,415); 16.0% (16,131/95,415); 17.0% (16,265/95,415); 100.0% (95,415/95,415)
GENCODE 27
GENCODE 47
lncRNA−merge
BIGTranscriptome
Fantom Cat
GENCODE20+
MiTranscriptome
NONCODE
RefSeq
CHESS 3.1.2
LncBook v2.0
% of genes
100%
75%
50%
25%

C

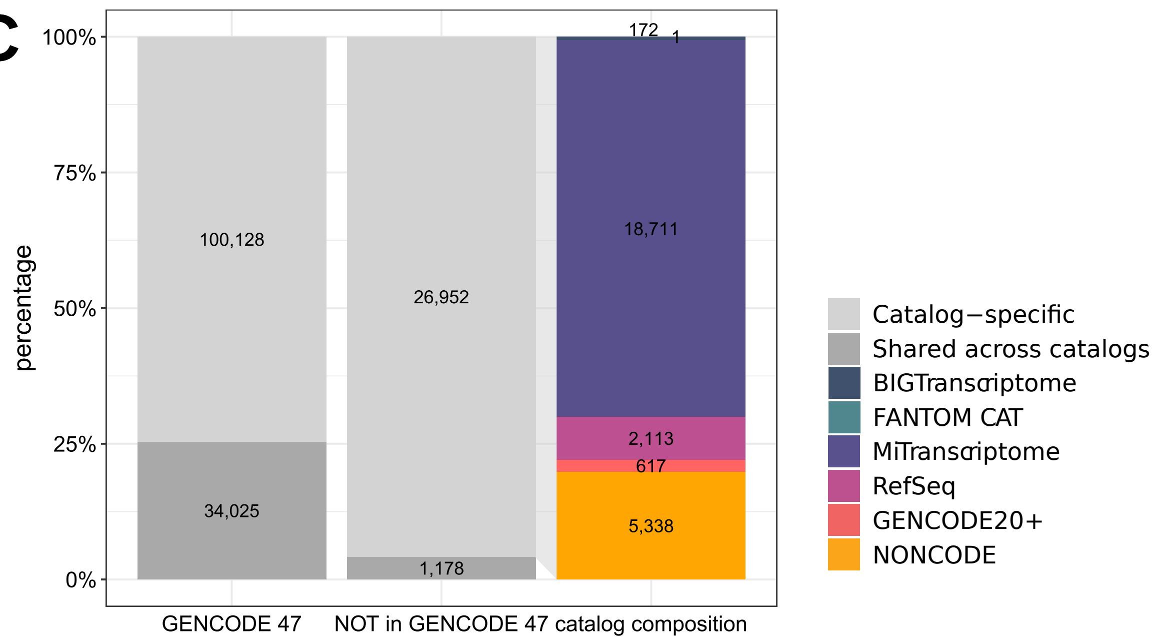
percentage
100%
75%
50%
25%
0%
100,128
34,025
26,952
1,178
172
1
18,711
2,113
617
5,338
GENCODE 47
NOT in GENCODE 47 catalog composition
Catalog−specific
Shared across catalogs
BIGTranscriptome
FANTOM CAT
MiTranscriptome
RefSeq
GENCODE20+
NONCODE

Fig S18

**A**

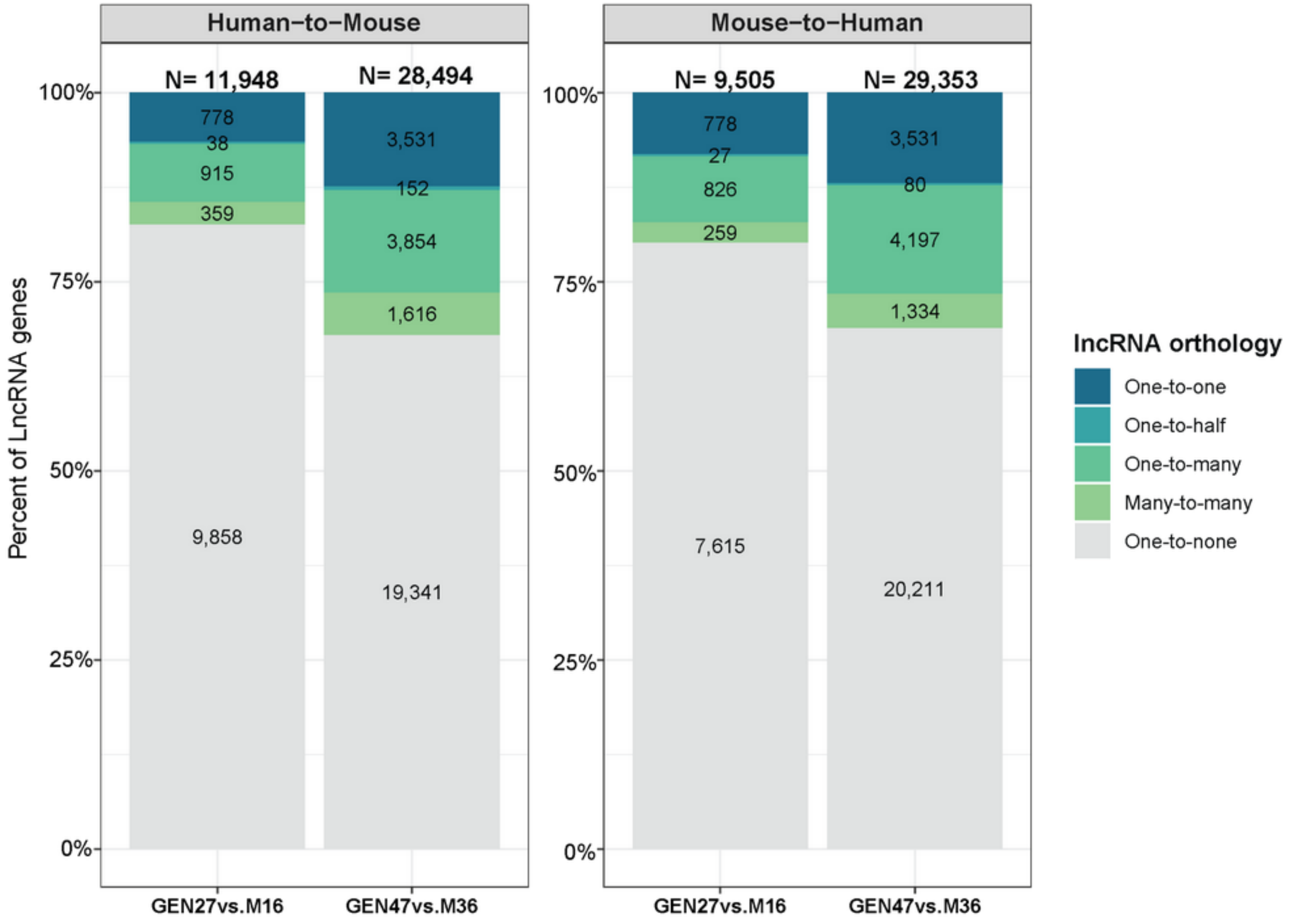


**B**

# Gene othology predictions

(Negative controls - lncRNAs)

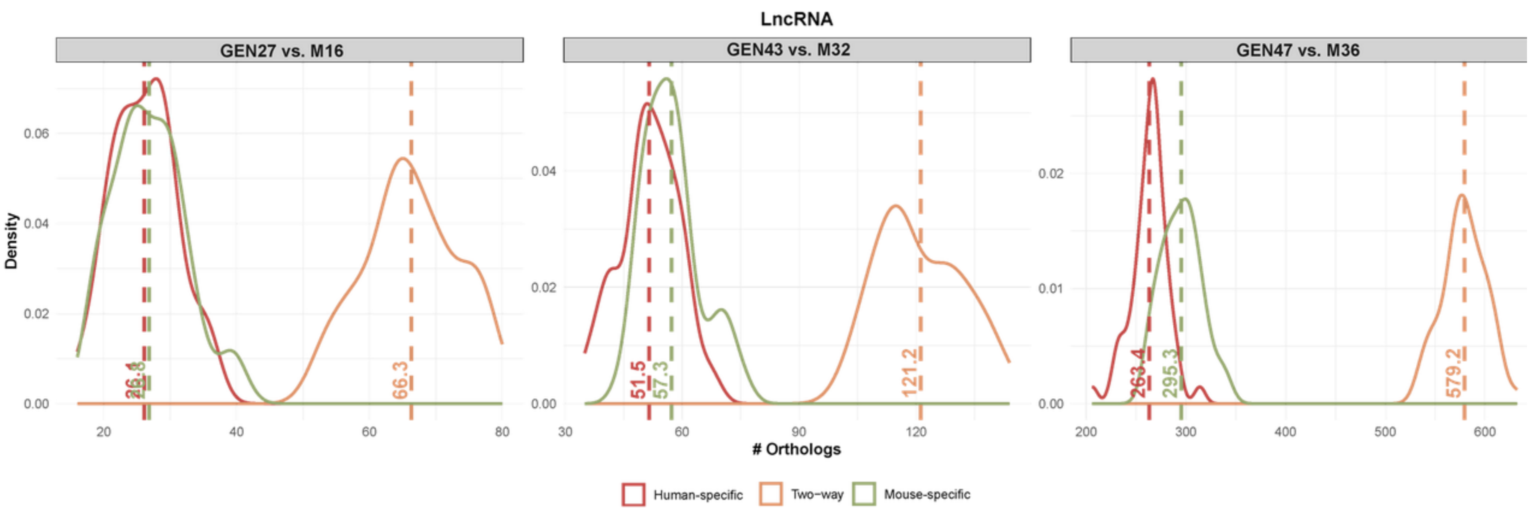

Fig S19

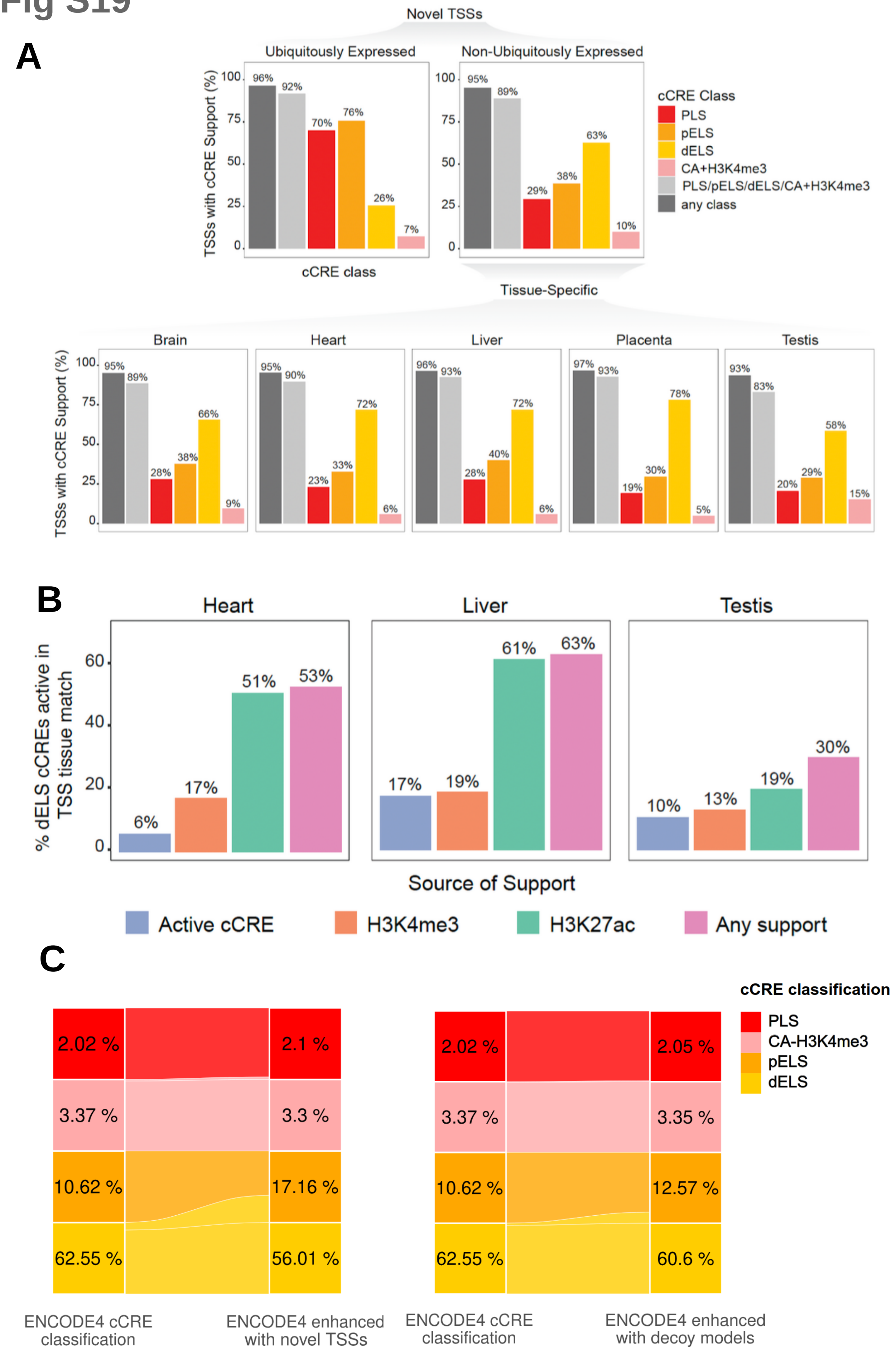
A
Novel TSSs
Ubiquitously Expressed
Non-Ubiquitously Expressed
TSSs with cCRE Support (%)
96%
92%
70%
76%
26%
7%
95%
89%
29%
38%
63%
10%
cCRE class
cCRE Class
PLS
pELS
dELS
CA+H3K4me3
PLS/pELS/dELS/CA+H3K4me3
any class
Tissue-Specific
Brain
Heart
Liver
Placenta
Testis
B
Heart
Liver
Testis
% dELS cCREs active in TSS tissue match
6%
17%
51%
53%
17%
19%
61%
63%
10%
13%
19%
30%
Source of Support
Active cCRE
H3K4me3
H3K27ac
Any support
C
cCRE classification
PLS
CA-H3K4me3
pELS
dELS
2.02 %
3.37 %
10.62 %
62.55 %
2.1 %
3.3 %
17.16 %
56.01 %
2.05 %
3.35 %
12.57 %
60.6 %
ENCODE4 cCRE classification
ENCODE4 enhanced with novel TSSs
ENCODE4 enhanced with decoy models

# Fig S20

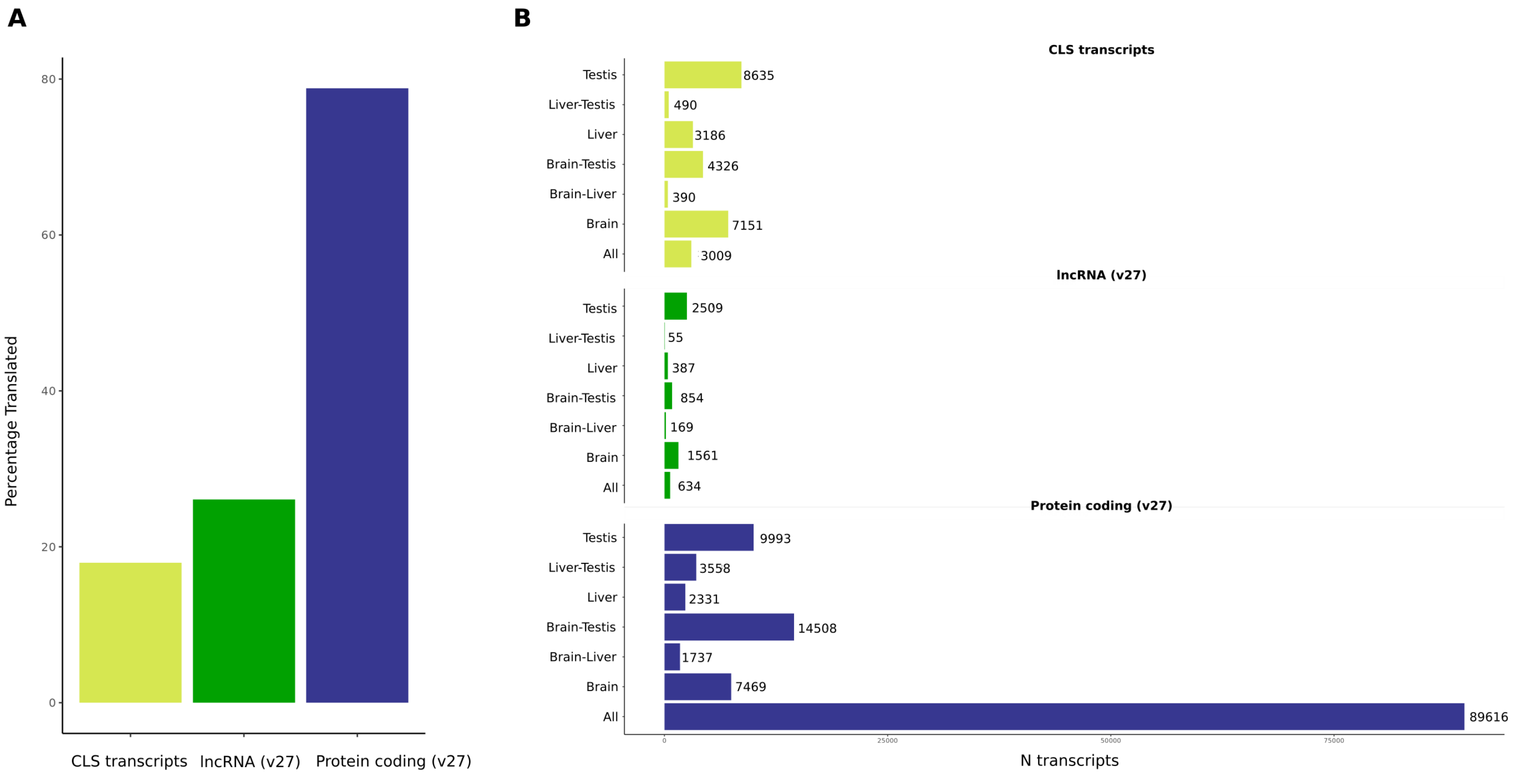
A
Percentage Translated
0
20
40
60
80
CLS transcripts
lncRNA (v27)
Protein coding (v27)
B
CLS transcripts
Testis 8635
Liver-Testis 490
Liver 3186
Brain-Testis 4326
Brain-Liver 390
Brain 7151
All 3009
lncRNA (v27)
Testis 2509
Liver-Testis 55
Liver 387
Brain-Testis 854
Brain-Liver 169
Brain 1561
All 634
Protein coding (v27)
Testis 9993
Liver-Testis 3558
Liver 2331
Brain-Testis 14508
Brain-Liver 1737
Brain 7469
All 89616
0
25000
50000
75000
N transcripts

# Fig S21

## A

| Reference | | |
|---|---|---|
| protein-coding (v27): | 5.54 | 12.78 |
| lncRNAs (v27): | 4.64 | 8.95 |
| inergenic CLS transcripts: | 4.71 | 10.03 |
| decoy models: | 3.41 | 5.21 |

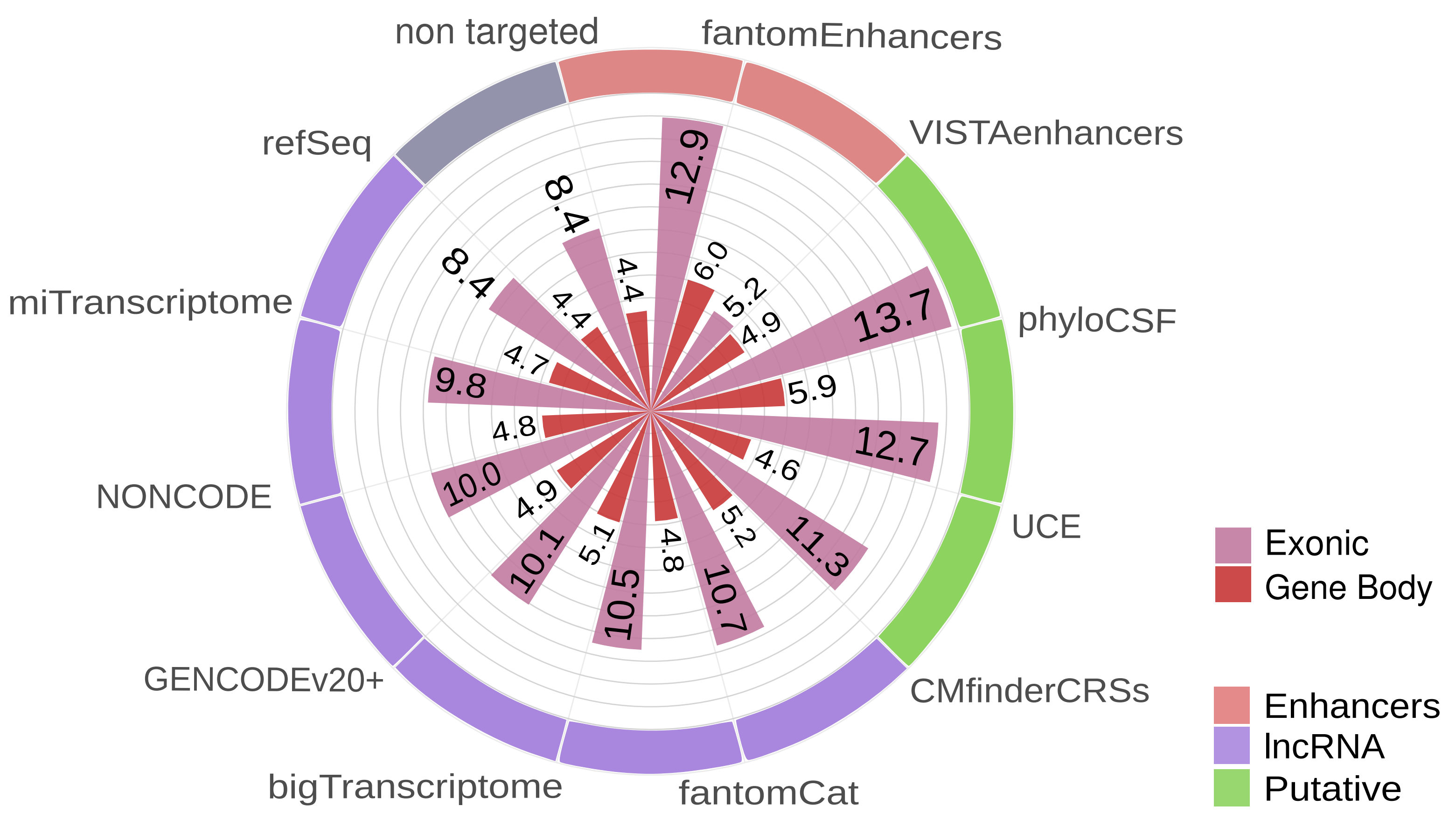


## B

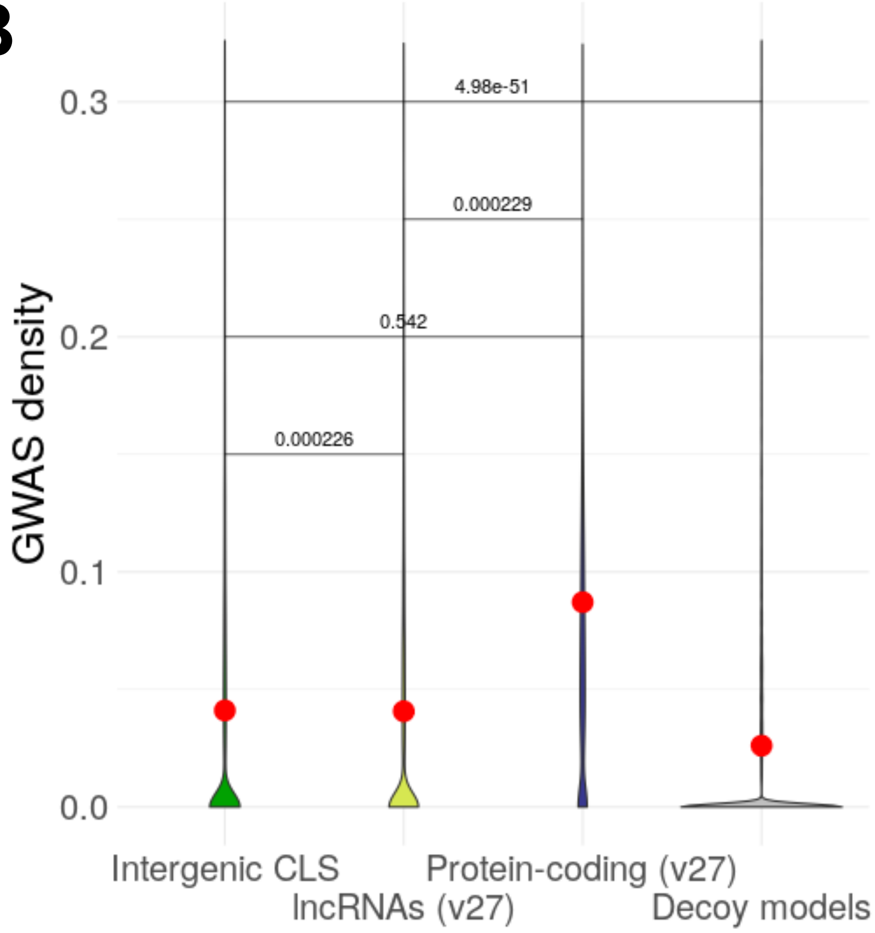

# Fig S22

**A**

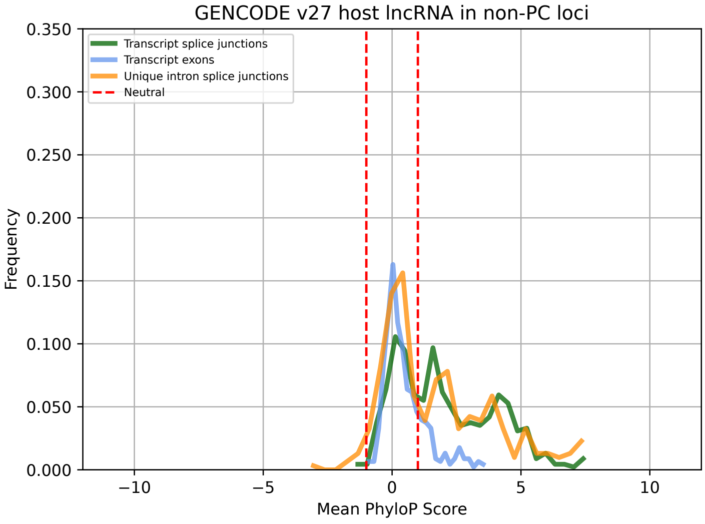

GENCODE v27 host lncRNA in non-PC loci
Transcript splice junctions
Transcript exons
Unique intron splice junctions
Neutral
Frequency
Mean PhyloP Score
0.350
0.300
0.250
0.200
0.150
0.100
0.050
0.000
−10
−5
0
5
10


**B**

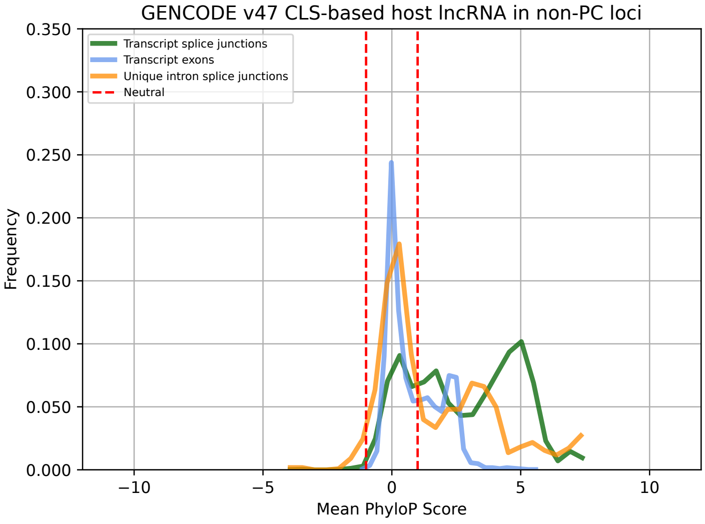

GENCODE v47 CLS-based host lncRNA in non-PC loci
Transcript splice junctions
Transcript exons
Unique intron splice junctions
Neutral
Frequency
Mean PhyloP Score
0.350
0.300
0.250
0.200
0.150
0.100
0.050
0.000
−10
−5
0
5
10
